# Flavonoids against non-physiologic inflammation attributed to cancer initiation, development, and progression—3PM pathways

**DOI:** 10.1007/s13167-021-00257-y

**Published:** 2021-10-06

**Authors:** Peter Kubatka, Alena Mazurakova, Marek Samec, Lenka Koklesova, Kevin Zhai, Raghad AL-Ishaq, Karol Kajo, Kamil Biringer, Desanka Vybohova, Aranka Brockmueller, Martin Pec, Mehdi Shakibaei, Frank A. Giordano, Dietrich Büsselberg, Olga Golubnitschaja

**Affiliations:** 1 Department of Medical Biology, Jessenius Faculty of Medicine, Comenius University in Bratislava, 03601 Martin, Slovakia; 2 Department of Obstetrics and Gynecology, Jessenius Faculty of Medicine, Comenius University in Bratislava, 03601 Martin, Slovakia; 3 Biomedical Centre Martin, Jessenius Faculty of Medicine in Martin, Comenius University in Bratislava, Mala Hora 4D, 036 01 Martin, Slovakia; 4 Weill Cornell Medicine-Qatar, Education City, Qatar Foundation, Doha, 24144 Qatar; 5 Department of Pathology, St. Elizabeth Cancer Institute Hospital, 81250 Bratislava, Slovakia; 6 Department of Anatomy, Jessenius Faculty of Medicine, Comenius University in Bratislava, 03601 Martin, Slovakia; 7 Musculoskeletal Research Group and Tumor Biology, Chair of Vegetative Anatomy, Faculty of Medicine, Institute of Anatomy, Ludwig-Maximilian-University Munich, Munich, Germany; 8 Department of Radiation Oncology, University Hospital Bonn, Rheinische Friedrich-Wilhelms-Universität Bonn, Bonn, Germany; 9 Predictive, Preventive and Personalised (3P) Medicine, Department of Radiation Oncology, University Hospital Bonn, Rheinische Friedrich-Wilhelms-Universität Bonn, Bonn, Germany

**Keywords:** Natural substances, Phytochemicals, Flavonoids, ROS, DNA damage, Carcinogenesis, Inflammation, Low-grade systemic inflammation, Cancer, Cancer initiation, Cancer promotion, Cancer progression, Systemic hypoxic-ishemic effects, Pre-metastatic niches, Impaired healing, Molecular targets, NF-κB signaling, Inflammatory pathway, HIF-1α, TNF-α, Cytokines, IL-1β, IL-6, IL-8, S100, Tissue remodeling, Matrix metalloproteinases, Predictive preventive personalized medicine (PPPM / 3PM)

## Abstract

Inflammation is an essential pillar of the immune defense. On the other hand, chronic inflammation is considered a hallmark of cancer initiation and progression. Chronic inflammation demonstrates a potential to induce complex changes at molecular, cellular, and organ levels including but not restricted to the stagnation and impairment of healing processes, uncontrolled production of aggressive ROS/RNS, triggered DNA mutations and damage, compromised efficacy of the DNA repair machinery, significantly upregulated cytokine/chemokine release and associated patho-physiologic protein synthesis, activated signaling pathways involved in carcinogenesis and tumor progression, abnormal tissue remodeling, and created pre-metastatic niches, among others. The anti-inflammatory activities of flavonoids demonstrate clinically relevant potential as preventive and therapeutic agents to improve individual outcomes in diseases linked to the low-grade systemic and chronic inflammation, including cancers. To this end, flavonoids are potent modulators of pro-inflammatory gene expression being, therefore, of great interest as agents selectively suppressing molecular targets within pro-inflammatory pathways. This paper provides in-depth analysis of anti-inflammatory properties of flavonoids, highlights corresponding mechanisms and targeted molecular pathways, and proposes potential treatment models for multi-level cancer prevention in the framework of predictive, preventive, and personalized medicine (PPPM / 3PM). To this end, individualized profiling and patient stratification are essential for implementing targeted anti-inflammatory approaches. Most prominent examples are presented for the proposed application of flavonoid-conducted anti-inflammatory treatments in overall cancer management.

**Supplementary Information:**

The online version contains supplementary material available at 10.1007/s13167-021-00257-y.

## Introduction

Inflammation is a physiological process necessary for homeostasis. Inflammation plays an elementary role in host defense against various pathogens through the activation and recruitment of immune cells and the consequent actions of innate and adaptive immunity. It is imperative for processes such as tissue regeneration, repair, and remodeling. Furthermore, its subtle manifestations are essential for the modulation of tissue homeostasis [1]. Cancer cells frequently secrete several growth factors that stimulate myelopoiesis and recruit myeloid cells to TME. Therefore, the TMEs of various cancers are characterized by the high infiltration of monocytes, macrophages, granulocytes, and dendritic cells. Most myeloid cells within TMEs are present in an immature form; however, cancer-derived growth factors modify these myeloid cells into cells that support carcinogenesis by enhancing proliferation, migration, and metastasis and enabling cancer cell survival and immune evasion [2].

Acquired data from the oncology research is applicable in the novel clinical trend focused on 3PM strategies considered as the medicine of the future [3, 4]. The presence of inflammatory or immune cells in specific tissues/organs was evaluated as a predictive marker in cancer therapy. Long-term oncology research indicates that chronic inflammation is associated with increased cancer risk and worsened disease prognosis [5]. Controlled inflammatory processes include specific immune responses—mainly linked with the maturation of antigen-presenting cells that promote tissue/organ healing. On the other hand, amplified, uncontrolled, or prolonged inflammation associated with immunosuppression is linked with many pathologies, including carcinogenesis [6]. It is widely accepted that up to 25% of neoplasms in humans are associated with chronic inflammation induced by viral and/or bacterial infections, physical and/or chemical stimuli, or metabolic pathologies [6–8]. Chronic inflammation influences tumor initiation and development by modulating critical signaling pathways associated with cellular transformation, proliferation, angiogenesis, survival, invasion, and metastasis [9]. The inflammation that initiates carcinogenesis exists long before cancer appears. In this regard, conditions such as chronic hepatitis, *Helicobacter*-induced gastritis, inflammatory bowel disease, and schistostoma-induced bladder inflammation increase the risk of several cancers, such as liver cancer, stomach cancer, colorectal cancer, and bladder cancer [8]. Numerous environmental factors must be considered, as they initiate and/or promote carcinogenesis by inducing organ-specific or systemic chronic inflammation. For instance, asbestos and tobacco smoke can induce lung and airway inflammation and thus initiate mesothelioma and lung carcinoma [10].

Similarly, low-grade systemic inflammation induced by hyperglycemia, hyperlipidemia, and obesity is a risk factor for several cancers, such as liver, pancreatic, colon, and breast cancers [11]. Abundant scientific evidence points out a significant role of systemic inflammation in cancer initiation, development, and progression. Obesity-, tobacco smoke–, and bacterial product–induced inflammation stimulates neutrophils and causes extracellular trap formation; these processes initiate cancer invasion [12]. The evaluation and modulation of the biological balance between controlled inflammation and uncontrolled chronic inflammation are essential for cancer prediction, prognostication, and prevention.

In the search for novel therapeutic agents that can precisely modulate cancer-associated inflammation, plant-derived natural products hold great promise due to their multi-targeting modes of action. Plant-derived compounds potently modulate inflammation. In the past several decades, many studies reported the anti-inflammatory effects of various phytochemicals in vitro and in vivo. In this regard, flavonoids could directly modulate the immune system [13]. Flavonoids exert anti-inflammatory actions through the regulation of immune cells, suppression of pro-inflammatory transcription factors, chemokines, cytokines, and COX-2, and inhibition of PI3K/AKT and IKK/JNK [14, 15].

This article summarizes the most recent evidence regarding the role of flavonoids as suppressors of pro-inflammatory cell signaling pathways that can promote carcinogenesis. The evidence supports the preventive and therapeutic potential of flavonoids in cancer.

## The role of chronic inflammation in different stages of carcinogenesis

### Cancer initiation

Two main cellular events are necessary for cancer initiation: first, the accumulation of genetic and/or epigenetic changes in genes that control tumor suppression/oncogenic signaling pathways; and second, the formation of transformed and/or malignant clones. A qualitative change into a tumor follows; the pro-inflammatory microenvironment can significantly support this process. A prolonged pro-inflammatory microenvironment contributes to genomic instability and cancer initiation. Therefore, pathogenic infections lead to DNA damage and/or cellular metabolic deregulation and thus change genomic integrity. The TME could trigger the recruitment of pro-inflammatory cells, making the environment rich in chemokines, growth factors, cytokines, and DNA-damaging agents (ROS, RNS) that activate DNA damage response pathways. Long-term deregulation of these factors may cause abnormalities and related pathologies, including cancer [16]. Moreover, the adaptive nature of carcinogenesis is highlighted because cell clones with equal genetic changes have different propensities for development and survival. These processes are strongly affected by the TMEs of these clones [17].

Chronic inflammatory processes are associated with oxidative/nitrosative stress, and consequent electron “storms” in cells/tissues induce progressive DNA damage. ROS/RNS originate from the immune system via specific NADPH oxidases that are crucial for pathogen clearance [18]. These DNA changes are linked with the deregulation of cellular homeostasis and result in genetic aberrations that act as initiation factors in chronic inflammation–induced pathogenesis, including carcinogenesis [19]. For example, chronic inflammation and ROS generation in colon epithelial cells can affect the Wnt/*β*-catenin and/or base excision repair pathways and thus increase the risk of polyp formation. The combination of DNA damage caused by oxidative stress and errors in DNA polymerase activity can support C>T transitions in various tissues, leading to a hypermutated cellular phenotype [20]. In another study using an inflammatory colorectal cancer model, the EMT factor ZEB1 promoted inflammation and progression towards inflammation-driven carcinoma by suppressing the DNA repair glycosylase MPG in epithelial cells [21]. Gobert et al. (2017) reported that polyamine- and NADPH-dependent ROS generation during *Helicobacter pylori* infections might lead to the initiation of inflammation and consequent carcinogenesis [22].

During chronic inflammation, pro-inflammatory mediators are continuously produced. Cytokines, growth factors, and acute-phase proteins, all part of the TME modulate the inflammatory responses and the crosstalk between signaling pathways involved in cancer initiation. Several papers describe the key role of the pro-inflammatory microenvironment in tumor initiation. Following infection or injury, SAA represents a critical acute phase protein secreted by hepatocytes during the APR. Autophagy is reported to be critical in tumors—both pro- and anti-carcinogenic. High levels of SAA in the TME contribute to carcinogenesis. SAA affects cell-signaling pathways, such as those associated with PI3K and MAPK, which modulate autophagy. Dysregulation of autophagy can initiate the transformation of normal cells to premalignant via metabolic stress, DNA damage, oxidative stress, and endoplasmic reticulum stress. In addition, autophagy can support the survival of transformed cells [23]. For instance, the type A receptor of IL-17 directly triggers pro-carcinogenic signaling in transformed enterocytes.

Moreover, the type A receptor of IL-17 stimulates ERK, p38 MAPK, and NF-κB signaling and induces the proliferation of transformed enterocytes that lack APC tumor suppressor functions [24]. Beneforti et al. (2020) hypothesized that infections and inflammation are the most important triggers of mutation accumulation in and the malignant transformation of ETV6-RUNX1 fusion gene (E/R^+^) pre-leukemic cells in childhood acute lymphoblastic leukemia. The authors showed that the pro-inflammatory cytokines IL-6/TNF-α/IL-1β coact with BM-MSC in promoting the emergence of E/R^+^ Ba/F3 cells (a murine IL-3-dependent pro-B cell line) and modulating their survival and proliferation. In addition, BM-MSCs attracted E/R^+^ Ba/F3 cells in a chemokine type 2 receptor–dependent manner; E/R^+^ human CD34^+^ IL7R^+^ progenitors (a supposed population of cells for acute lymphoblastic leukemia) were protected in the presence of BM-MSC and IL-6/TNF-α/IL-1β. The authors concluded that DNA damage accumulation depends on the extent of inflammation in both control and E/R^+^ Ba/F3 cells and might lead to the transformation of the apoptosis-resistant pre-leukemic clones [25]. Furthermore, Huang et al. (2020) found that IL-1β, secreted by macrophages stimulated by multi-walled carbon nanotubes, increased the release of pro-inflammatory cytokines such as TNF-α, IL-8, and IL-6 from mesothelial cells. The authors concluded that inflammation-induced NF-κB (p65)/IL-6/STAT3 signaling plays a crucial role in the malignant transformation of pleural mesothelial cells [26].

Different cytokines and chemokines may modulate the conversion of normal fibroblasts into CAFs. CAFs synthesize pro-inflammatory cytokines such as IL-6 and LIF, which in turn modulate their epigenetic status. This activity promotes the pro-tumorigenic function of CAFs by enhancing actomyosin contractility and ECM remodeling [27]. IL-1β is another pro-inflammatory cytokine that initiates carcinogenesis and promotes immunosuppression. These functions of IL-1β are mainly observed in the early stages of carcinogenesis; moreover, its expression significantly impacts the process of malignant transformation [28].

### Cancer promotion

Like cancer initiation, a pro-inflammatory TME and consequent signaling can support numerous growth factors in promoting carcinogenesis. Cancer promotion can be regulated by several pathways associated with inflammation, such as those involving NF-κB, STAT3, mTOR, and MAPKs, which are triggered by pro-inflammatory cytokines like IL-6, TNF-α, and IL-1β [29]. Ju et al. (2020) revealed that infiltrated macrophages released TNF-α and IL-6, which induced the expression of PD-L1 in cancer cells. Consequently, TNF-α and IL-6 triggered the activation of the NF-κB and STAT3 signaling pathways to modulate PD-L1 expression. The authors concluded that macrophage-induced increases in PD-L1 expression could allow cancer cells to escape from cytotoxic T cell surveillance and proliferate [30]. PIM2 is an oncomarker highly expressed in HCC which correlates with poor prognosis. Functional studies demonstrated that PIM2 is an enhancer of cell proliferation, cell motility, angiogenesis, and chemoresistance in cancer. In this regard, HCC cells treated with TNF-α showed increased PIM2 expression; this consequently increased the expression of TNF-α. The same study revealed that PIM2 promotes HCC carcinogenesis by activating the NF-κB signaling pathway through PIM2 receptor phosphorylation [31].

S100A7/RAGE signaling in adjacent endothelial cells may behave as a crucial angiocrine effector. Muoio et al. (2021) found that IGF-1/IGF-1R signaling increases STAT3 activation in breast cancer cells. Consequently, activating the S100A7/RAGE pathway via STAT3 signaling sustains angiogenesis and thus stimulates cancer promotion in breast cells [32]. The upregulation of cancer-promoting inflammatory cytokines (host and tumor-secreted), such as NF-κB, COX-2, IL-1, IL-6, TNF-α, and IFN-γ, is strongly associated with STAT3 and AKT signaling activation in oral squamous cell carcinoma in vitro. The suppression of these cytokines may decrease tissue inflammation and thus decrease the proliferation of oral squamous cell carcinoma cells [33]. TAMs play a substantial role in modulating the interactions between cancer cells and the immune system. In this regard, the TAM microenvironment promoted the growth of triple-negative breast cancer cells in a study by Deng et al. (2021). The authors described that TAMs promoted the growth of MDA-MB-231 and MDA-MB-468 cells by upregulating the IL-10/STAT3/PD-L1 immunosuppressive signaling pathway [34]. Zhang et al. (2017) revealed that TAMs might trigger PD-L1 expression by producing IFN-γ via the JAK/STAT3 and PI3K/AKT signaling cascades in A549 cells [35].

Recent data revealed that MAPK upregulation is crucial in modulating inflammation-associated cancer development. MAPKs include p38 MAPK, JNK, and ERK. These enzymes are serine-threonine protein kinases that affect essential cellular activities, such as proliferation, differentiation, apoptosis, survival, inflammation, and innate immunity. The described enzymes are upregulated by various types of cellular stress and pro-inflammatory cytokines such as TNF-α and IL-1β [36]. In addition, increased p38 MAPK signaling is linked with increased proliferation and decreased apoptosis of colon and liver cancer cells. Deregulation of the mTOR signaling pathway, including the loss of *PTEN* function, amplification/mutation of *PI3K*, and overexpression of S6K1, 4EBP1, eIF4E, and AKT, was described in multiple cancer types but mainly in melanoma [37]. In a preclinical study, lung cancer–induced osteoclastogenesis in vitro was associated with the upregulation of IL-6 and TNF-α and consequent activation of the AMPK/mTOR signaling pathway [38].

### Cancer progression

Cancer metastasis is a highly inefficient process. Most cancer cells released from the primary tumor site die before creating a distant metastatic site. To further improve the ratio of dead cancer cells with invasive phenotypes, it is possible to modulate specific “key steps” within the complex process of tumor progression that may decrease the “success” of metastatic invasion and improve patient survival. Metastasis begins with the invasion of tumor cells from the epithelium into the surrounding tissues and the concurrent EMT. The post-EMT phenotype of cells (often only partial) allows them to cross the basal epithelial membrane and reach lymphatic and/or blood capillaries [8, 39]. A recent study described a novel lymphatic pattern in the hypoxic TME, wherein TAMs encapsulate lymphatic vessels to form an interconnected network. These aggregates are advantageous for and actively involved in early lymph node metastasis [40].

Moreover, research data revealed the importance of the pro-inflammatory TME within the ECM in modulating tumor-associated processes such as invasion, metastasis, angiogenesis, immune cell modulation, and therapeutic resistance [41]. Calon et al. (2015) reported that all colorectal cancer subtypes with poor prognosis are characterized by stromal cancer cells with a TGF-β-triggered transcription program associated with a pro-inflammatory TME. The application of TGF-β signaling inhibitors suppressed the crosstalk between the TME and cells and thus suppressed cancer progression in patient-derived tumor organoids and xenografts [42].

CSCs are more effective in metastasis than other (bulk) tumor cells. CSCs are essential for cancer metastasis and therapeutic resistance. Numerous stimuli such as chronic inflammatory signaling, including the activation of the STAT3 and NF-κB transcription factors, may drive the stemness of CSCs in cancer tissue and enlarge their proportion within the cell population, thereby increasing the potential of metastasis. Importantly, CSCs are functionally and transcriptionally more related to mesenchymal cells than bulk tumor or normal epithelial cells [43]. Cytokines and chemokines, both their receptors and signaling pathways, are essential factors in the complex interplay and crosstalk between different cell types and secreted factors in the events that lead to cancer metastasis. Numerous cytokines and chemokines, such as TNF, IL-6, IL-8, CXCL12, TGF-β, CXCL8, VEGF, RANKL, CCL2, CX3CL1, IL-1, IL-7, CXCL1, and CXCL16, contribute to the regulation of cancer metastasis [44, 45].

Moreover, comprehensive data support the role of a pro-inflammatory TME in the promotion of CSCs. Cytokines and chemokines may benefit cancer cells and/or CSCs in processes such as cell proliferation, survival, and migration [46]. In this regard, there exists a close linkage between CSCs and inflammatory components, i.e., inflammatory cells such as TAMs and MDSCs and inflammatory cytokines (TNF, IL-6, IL-17, IFNs) [47].

Metastatic spread typically occurs through blood and lymphatic vessels. Therefore, intravasation and extravasation are essential processes in cancer metastasis. These processes are modulated by specific adhesion molecules and integrins facilitating cell-cell interactions/adhesion and cell movement. In this regard, inflammatory cytokines are important inducers of integrins, selectins, and adhesion molecules, such as VCAM-1 and ICAM-1. In addition, TAMs produce pro-angiogenic growth factors and MMPs. Therefore, they support vasculogenesis and consequently the supply of oxygen and nutrients to solid tumors [48]. A preclinical study by Horiguchi et al. (2020) demonstrated that local inflammation, characterized by elevated IL-6, TNF-α, and HGF in the bronchoalveolar lavage fluid, increased the metastatic activity of NL-17 cells in the lung. A pro-inflammatory TME significantly upregulated the expression of α2 integrin, VCAM-1, and ICAM-1. An anti-ICAM-1 antibody suppressed the invasive activity of NL-17 cells; therefore, adhesion molecules have a potential role in lung metastasis enhanced by local inflammation [49].

Comprehensive research has described specific cellular and molecular events that may cause tumor cells to escape immune surveillance, including those involving tumor-induced myeloid cell–mediated immunosuppression [50]. Multiple studies indicate that tumor-infiltrating myeloid cells accelerate cancer growth and support angiogenesis, metastasis, and therapeutic resistance after their conversion into potent immunosuppressive cells. Host and tumor cells in the TME secrete pro-inflammatory molecules that stimulate MDSCs and trigger their accumulation and suppressive activities [51]. Several mechanisms driven by MDSCs suppress T cells and activate immunosuppressive cell populations. Such inflammatory modulation in the TME causes the immune system to tolerate cancer cells and enhance their growth.

In conclusion, the interruption of the interplay between pro-inflammatory cytokines and pro-inflammatory/pro-carcinogenic cell signaling pathways affects all stages of carcinogenesis and might therefore represent a promising strategy in oncology research and practice.

## Anti-inflammatory activities of flavonoids: implications in carcinogenesis

Flavonoids constitute a group of natural substances with different phenolic structures. They are found in fruits, vegetables, grains, flowers, tea, bark, roots, and stems. These natural molecules have well-described beneficial health effects in humans. Due to their anti-oxidative, anti-inflammatory, anti-mutagenic, anti-carcinogenic, and cellular enzyme modulating activities, flavonoids have numerous medicinal, pharmaceutical, and cosmetic applications. A substantial proportion of non-infectious diseases develops or is worsened by the chronic inflammatory process. Flavonoids are reported to combat most inflammatory processes underlying chronic conditions such as carcinogenesis [52–61].

### Cancer initiation

Pro-inflammatory cytokine production and consequent intracellular ROS and RNS accumulation lead to DNA damage and initiate carcinogenesis. Flavonoids, as antioxidants, inhibit regulatory enzymes and transcription factors important for controlling inflammatory mediators. Moreover, they modulate cellular oxidative stress by interacting with DNA and enhancing genomic stability [62]. Flavonoids can inhibit DNA adduct formation, enhance DNA repair by interfering with genotype damage caused by the upregulation of phase II enzymes, and modify relevant signaling pathways [63]. It is well documented that specific flavonoids can reduce cytokine production; therefore, they may have preventive and therapeutic potential in inflammation-related diseases (such as cancer) [64]. TNF-α triggers the release of chemotactic proteins, e.g., MCP-1/CCL2. These regulatory molecules direct the infiltration and migration of TAMs, MDSCs, Tregs, TANs, Th17s, MAMs, and CAFs. Specific flavonoids can attenuate the TNF-α-induced release of MCP-1/CCL2 and various recruiting cytokines from cancer cells [65, 66]. In addition, by targeting TGF-β2, flavonoids suppress the cancer cell–mediated differentiation of naive fibroblasts into cancer-associated fibroblasts [67].

### Cancer promotion

The inhibitory effect of flavonoids on cell proliferation and thus cancer promotion is associated with decreased phosphorylation of STAT-induced signaling pathway components and related transcriptional activators. Hou et al. (2019) revealed that flavonoid treatment inhibits recurring colitis and colitis-associated tumorigenesis; these flavonoids downregulate IL-1β and TNF-α and consequently inhibit inflammation-induced colorectal cancer in vitro [68]. NF-κB signaling plays a crucial role in inflammation and cancer growth. Flavonoids negatively regulate the NF-κB signaling pathway by suppressing kinase phosphorylation, inhibiting NF-κB translocation into the nucleus, and blocking interactions between DNA and NF-κB. Through these mechanisms, flavonoids inhibit inflammatory cascades associated with decreased cell proliferation, apoptotic induction, and the suppression of vasculogenesis [69]. In addition, flavonoids are promising anti-cancer agents that target the inflammatory PI3K/AKT/mTOR/p70S6K and ERK/MAPK signaling pathways [70, 71].

### Cancer progression

Flavonoids exert chemopreventive effects by protecting against cancer progression, inhibiting CSC formation, and alleviating lung metastasis in a preclinical model [72]. The reduction in cancer metastasis was associated with regulating the PI3K/AKT, MAPK/ERK, and STAT3 pathways—central CSC-associated inflammatory signaling cascades. In another study, natural mixtures of flavonoids significantly downregulated the pro-inflammatory cytokines COX-2, iNOS, and TNF-α and the pro-angiogenic factors VEGF and eNOS, and induced apoptosis by increasing the Bax/Bcl-2 ratio in vitro [73]. In this regard, flavonoids are anti-inflammatory agents that downregulate crucial modulators of advanced stages of cancer such as IL-1β, IL-6, IL-10, TNF-α, NF-κβ, NOS2, PTGS2, PTGER2, ACAN, COL2A1, MMP1, MMP13, ADAMTS4, ADAMTS5, and TIMP1. These changes correlated with the reduction of serum levels of PGE2, CTX-II, TNF-α, MMP1, MMP13, PIINP, OPG, RANKL, OC, and BALP in a rat model [74]. Another study found that flavonoids exert their anti-metastatic activities by reducing CXCR4 expression and can therefore support the blockade of cancer promotion [75]. Furthermore, flavonoids target the integrin-modulated ILK/YAP pathway and block the EMT and metastasis [76]. Moreover, flavonoids block angiogenesis and metastasis by suppressing VEGF-induced oxidative stress and NF-κB signaling and downregulating adhesion molecules such as VCAM-1, ICAM-1, and E-selectin; these activities decrease the formation of new blood capillaries in cancer tissue [77]. Finally, MDSC activation is involved in chronic inflammation-related immunosuppression and CD4^+^/CD8^+^ T cell activation through the ERK/IL-6/STAT3 and Arg-1/iNOS/Nox2/NF-κB/STAT3 signaling pathways. In this regard, flavonoids attenuate MDSC-mediated immunosuppression that is crucial for cancer growth and metastasis [78, 79].

## Flavonoids prevent tumor initiation by modulating inflammatory processes

The concept of cancer chemoprevention by flavonoids was investigated in numerous in vivo and in vitro studies [80–84]. These natural phenolic compounds can modulate different steps of carcinogenesis [85]. As mentioned above, chronic inflammation accelerates genetic/epigenetic aberrations that lead to cancer initiation, promotion, and progression [86]. Prolonged chronic inflammation associated with the overexpression of inflammatory mediators in the cell microenvironment is a critical step that promotes cancer initiation [87]. The anti-inflammatory properties of flavonoids could prevent, suppress, and reverse cancer initiation [88, 89]. In the following section of the manuscript, we provide an overview of recent studies that analyze the role of flavonoids in tumor initiation through the modulation of inflammatory responses in vitro and in vivo.

### Preclinical research

Baicalin, a flavonoid isolated from the roots of *Scutellaria baicalensis*, exerts different oncostatic effects [90, 91]. ROS generation due to constant UV-A exposure causes the formation of inflammatory products and the accumulation of DNA mutations [92]. The protective role of baicalin was investigated in an animal study that analyzed the association between UV-A irradiation, ROS production, and inflammation. The acquired data revealed that baicalin protects against UV-A-induced inflammation and oxidative damage by increasing IL-12 and IL-23. These effects are likely mediated by suppressing the TLR4 pathway, which has a significant role in inflammation [93]. Additionally, baicalin can be transformed into baicalein by intestinal microbiota. The anti-inflammatory and anti-cancer effects of baicalein were evaluated in an animal model of colorectal cancer. The authors used a gut-specific C57BL/6J Apc^Min/+^/J mouse model (APC gene mutants) to evaluate parameters such as life span, tumor multiplicity, and organ index. Moreover, the expression of inflammatory cytokines was measured. Baicalein administration (30 mg/kg/day) decreased the number of tumors in the small intestine and colon (after 10 weeks of supplementation by baicalein) compared to controls. In addition, baicalein administration suppressed the expression of pro-inflammatory cytokines (IL-1β, IL-2, IL-6, IL-10, GM-CSF, and G-CSF) [94]. Ulcerative colitis is classified as a chronic idiopathic IBD [95]. Patients with IBDs are at increased risk for the development of extra-intestinal malignancies [96]. Rutin is a naturally occurring flavonoid with profound effects on different cellular processes associated with a pathological phenotype [97]. As demonstrated in a DSS-induced experimental colitis model in vivo, rutin modulated the expression of pro-inflammatory genes. Rutin administration significantly suppressed the protein level of IL-1β and the expression of *IL-1β* and *IL-6* mRNA in the colonic mucosa of mice. Moreover, rutin attenuated DSS-induced colitis symptoms, including weight loss and colorectal shortening, and improved the histological score of colitis in the tested animals [98]. Another flavonoid, myricetin, downregulates inflammatory factors including TNF-α, IL-6, IL-1β, NF-κB, p-NF-κB, PCNA, COX-2, and cyclin D1 in AOM/DSS-induced colitis in mice. Myricetin significantly reduced the number of colorectal tumors and decreased the size of polyps in the colon [99]. Furthermore, naringin prevented AOM/DSS-induced colitis and carcinogenesis in mice by suppressing MDSCs. A deeper analysis identified the downregulation of GM-CSF/M-CSF, IL-6, and TNF-α and the inhibition of the NF-κB/IL-6/STAT3 pathway as further contributors to colitis-associated cancer [100].

Chronic arsenic exposure is associated with inflammation that could initiate carcinogenesis [101]. EGCG, a natural compound isolated from green tea, is a potent anti-inflammatory molecule [102]. EGCG exhibited an anti-inflammatory effect against arsenic (NaAsO_2_)-induced inflammation in mice. The acquired data revealed that EGCG administration downregulated the pro-inflammatory cytokines IL-1β, IL-6, and TNF-α. Additionally, EGCG attenuated oxidative stress by upregulating markers such as CAT, SOD, and GSH and decreasing MDA content in mice [103]. Furthermore, the administration of GTPs influenced UV-B-induced tumor development in mice. Likewise, UV-B is strongly associated with chronic inflammation and, as mentioned above, there is a strong relationship between UV radiation–induced chronic inflammation and skin carcinogenesis. GTPs suppressed the pro-inflammatory markers COX-2, PGE2, PCNA, and cyclin D1. Furthermore, levels of the pro-inflammatory cytokines TNF-α, IL-6, and IL-1β were reduced after GTP administration [104]. Chronic gastritis and peptic ulceration—chronic inflammatory disorders caused by *Helicobacter pylori* infection—are strongly associated with an increased risk of cancer development [105, 106]. The isoflavonoid genistein exerted anti-inflammatory effects in rats infected by *Helicobacter pylori*. Its anti-inflammatory properties were mediated by suppressing pro-inflammatory mediators such as TNF-α and CINC-1, NF-κB, and bacterial infection–induced gastric epithelial cell apoptosis [107]. Hesperidin, a flavone extracted from citrus fruits, has many biological effects [108]. Its impact on ulcerative colitis was documented in a study that evaluated the anti-inflammatory effect of hesperidin methyl chalcone in an animal model of acetic acid–induced colitis*.* As demonstrated above, chronic inflammation promotes colitis-associated cancer; thus, the attenuation of pro-inflammatory cytokines is a promising way to suppress cancer initiation [109]. Hesperidin significantly catalyzed colon antioxidant processes and suppressed pro-inflammatory cytokines (IL-33, IL-1β, IL-6, and TNF-α) in vivo [110]*.* In addition, quercetin exhibited anti-inflammatory effects in another model of DSS-induced experimental colitis in vivo. Comalada et al. (2005) evaluated the effect of quercitrin, a glycoside of quercetin that is cleaved by gut microbiota to generate quercetin. Quercetin significantly inhibited cytokine (IL-1β and TNF-α) production and induced the expression of iNOS by downregulating the NF-κB pathway [111]. Moreover, the flavonoid luteolin possesses various beneficial properties for human health [112–114]. Luteolin significantly reduced malignant transformation induced by hexavalent chromium [Cr(VI)] in human bronchial epithelial cells (BEAS-2B). Recurrent exposure to [Cr(VI)] is connected to persistent inflammation and subsequent carcinogenesis [115]. Noteworthy, luteolin is known to downregulate AP-1, HIF-1α, COX-2, and iNOS. In addition, luteolin prevented the transformation of BEAS-2B into malignant phenotypes. The obtained data revealed decreases in IL-1β, IL-6, IL-8, and TNF-α levels. Western blotting uncovered the inhibition of products associated with inflammation, including MAPK, NF-κB, COX-2, STAT-3, and iNOS in chronic [Cr(VI)]-exposed cells. In addition, luteolin reduced tumor incidence in mice injected with [Cr(VI)]-exposed BEAS-2B cells [116]*.* Furthermore, cigarette smoke modulates inflammation and induces chronic inflammation. Therefore, Pace et al. (2019) evaluated the impact of CSE on normal (16HBE) and cancerous (A549) epithelial cells. The study authors applied the naturally occurring flavone apigenin, which reduced miR-21 and IL-8 gene expression in both cell lines [117].

As we described in this chapter, flavonoids possess pleiotropic abilities in preventing cancer initiation through the suppression of chronic inflammation. The health benefits of flavonoids targeting inflammatory pathways identified in experimental studies could be translated into the clinical area. The investigation of novel therapeutic approaches to reverse and prevent cancer initiation through the modulation of chronic inflammation falls within the concept of 3PM. Table 1 summarizes the effects of flavonoids on molecular cascades associated with inflammation and subsequent carcinogenesis.

**Table 1:** Flavonoids targeting inflammatory pathways associated with cancer initiation

Flavonoid	Study design	Mechanisms	Dosage of the tested flavonoid	References
Baicalin	Female C3H/HeN mice	Baicalin treatment inhibited ROS production via the downregulation of p47p^hox^, a key component of NADPH oxidase. Furthermore, baicalin inhibited inflammatory cascades through TLR4 suppression	4 mg baicalin per mouse	[93]
Baicalein	C57BL/6J ApcMin/+/J mouse model	Baicalein reduced the number of tumors in the small intestine (*P*<0.01) and colon (*P*<0.05) in the baicalein-treated group compared to the non-treated group. Additionally, ELISA analysis of small intestine and colon tissue revealed the downregulation of pro-inflammatory cytokines, including IL-1β, IL-2, IL-6, IL-10, GM-CSF, and G-CSF	30 mg/kg/day	[94]
Rutin	Female ICR mice	ELISA analyses revealed the downregulation of IL-1β in the colonic mucosa. Moreover, the mRNA levels of *IL-1β* and *IL-6* were decreased after rutin administration. Symptoms of DSS-induced colitis were attenuated by rutin	6 mg/day, 0.6 mg/day and 60 mg/day	[98]
Myricetin	Male BALB/c mice	Myricetin administration reduced tumorigenesis and inflammation in vivo. Western blot and qPCR analyses revealed decreases in the levels of pro-inflammatory markers (TNF-α, IL-6, IL-1β, NF-κB, p-NF-κB, PCNA, COX-2, and cyclin D1) in mice	40 mg/kg and 100 mg/kg	[99]
Naringin	Male C57BL/6 mice	Oral administration of naringin prevented colitis and carcinogenesis induced by AOM/DSS through the reduction of GM-CSF/M-CSF, IL-6, and TNF and inhibition of the NF-κB/IL-6/STAT3 pathway	50 and 100 mg/kg	[100]
EGCG	Male BALB/c mice	EGCG decreased oxidative stress (increased SOD, CAT, and GSH activity, and decreased MDA and nitric oxide). EGCG decreased the levels of pro-inflammatory cytokines, including IL-1β, IL-6, and TNF-α	10 mg/kg	[103]
GTPs	Female C3H/HeN mice, IL-12p40KO mice on a C3H/HeN background	GTPs inhibited UV-B-induced skin carcinogenesis by downregulating pro-inflammatory markers (COX-2, PGE2, PCNA, TNF-α, IL-6, and IL-1β) in wild-type mice. GTP administration in their counterparts, IL-12p40 knockout mice, was less effective than in WT mice.	Water containing GTPs (0.2%, w/v)	[104]
Genistein	Sprague-Dawley rats	Genistein inhibited *Helicobacter p*.-induced gastropathy by suppressing pro-inflammatory cytokine (TNF-α and CINC-1) production, NF-κB activity, and gastric cell apoptosis.	16 mg/kg	[107]
Hesperidin	Male Swiss and LysM-eGFP mice	Hesperidin demonstrated anti-inflammatory effects by inhibiting ROS generation and IL-33, IL-1β, IL-6, and TNF-α cytokine production through the inhibition of NF-κB. In addition, hesperidin attenuated colitis symptoms, including bowel edema, colon shortening, and macroscopic lesions.	10, 30, or 100 mg/kg in saline	[110]
Quercetin	Female Wistar rats	Administration with Quecetrin (1mg/kg/day) suppressed IL-1β, TNF-α, and iNOS expression through the inhibition of the NF-κB pathway in a rat model of dextran sulfate sodium–induced colitis.	1 mg/kg/day	[111]
Luteolin	BEAS-2B cells; [Cr(VI)]-induced BEAS-2B cells injected into mice	Luteolin treatment suppressed the promoter activity of AP-1, HIF-1α, COX-2, and iNOS and the production of IL-1β, IL-6, IL-8, and TNF-α in BEAS-2B cells. Western blot analysis revealed decreases in MAPK, NF-κB, COX-2, STAT-3, iNOS, and TNF-α protein levels in vitro. Reduction of tumor frequency in mice injected with [Cr(VI)]-exposed BEAS-2B cells	1 and 2 μM	[116]
Apigenin	16HBE and A549 cells	Apigenin reduced miR-21 and IL-8 mRNA expression in normal and cancerous cells exposed to CSE	20 μM	[117]

## Flavonoids as anti-inflammatory agents against tumor promotion in carcinogenesis

### Isolated flavonoids in preclinical studies

Chronic inflammation predisposes to cancer development and subsequently promotes all stages of tumorigenesis [8]. The anti-inflammatory and other anti-cancer effects of flavonoids were evaluated in cancer research since the last century. GTPs, silymarin, and apigenin inhibited skin tumor promotion by decreasing skin papilloma formation in DMBA-induced and TPA-promoted SENCAR mice [118–120].

As discussed above, various signaling pathways are associated with chronic inflammation, including the MAPK, AKT, mTOR, STAT3, and/or NF-κB pathways, and can potentially lead to tumor promotion. Therefore, in the twenty-first century, studies emphasize the precise molecular mechanisms and signaling pathways leveraged by flavonoids against inflammation in various stages of carcinogenesis.

Quercetin inhibited the PI3K/AKT/mTOR, Wnt/β-catenin, and STAT3 signaling pathways in BC3, BCBL1, and BC1 primary effusion lymphoma cells. Further, quercetin inhibited tumor promotion by downregulating the pro-inflammatory cytokines IL-6 and IL-10; pro-survival molecules downstream of PI3K/AKT/mTOR, Wnt/β-catenin, and STAT3 such as c-FLIP_L_; and molecules linked to cell proliferation, including cyclin D1 and cMyc [121].

Moreover, icaritin from a Chinese herbal medicine (*Epimedium* species) inhibited proliferation and tumor growth and induced apoptosis of K562 and primary chronic myeloid leukemia cells. A mouse model also revealed that icaritin modulates signaling pathways associated with chronic inflammation such as MAPK/ERK/JNK and JAK2/STAT3/AKT by upregulating p-JNK and p-C-JUN and downregulating p-ERK, p-P38, JAK-2, p-JNK, p-STAT3, and p-AKT in a dose- and time-dependent manner [122].

Furthermore, fisetin suppressed the expression of inflammatory mediators and ICAM-1 in IL-1β-promoted inflammation in A549 lung adenocarcinoma cells. Further, administrating fisetin downregulates COX-2, PGE2, IL-8, CCL5, MCP-1, TNF-α, and IL-6. Moreover, fisetin exerted anti-inflammatory effects through the suppression of the NF-κB and ERK1/2 signaling pathways in IL-1β-stimulated A549 cells, suggesting to prevent tumor promotion [123]. In another study, fisetin induced apoptosis by increasing caspase-3 expression and regulating the inflammatory PI3K/AKT/NF-κB signaling pathway in TU212 head and neck squamous cell carcinoma cells. Further, the suppression of TU212 cell proliferation and consequent changes in the tumor volume and weight of nude mice after fisetin treatment were connected to decreased Ki67 levels and the inactivation of ERK1/2- and PI3K/AKT-regulated mTOR [124]. Additionally, the combination of fisetin with carnosic acid enhanced their anti-inflammatory effects against tumor promotion of HCC827 and H358 human lung cancer cell lines and their murine xenografts. Co-treatment of fisetin and carnosic acid induced apoptosis by upregulating caspase-3, Bax and Bad, and death receptor of TRAIL, and downregulating the anti-apoptotic Bcl-2 and Bcl-xl proteins [125].

Interestingly, luteolin inhibited U251 and LN229 glioma cell proliferation through the induction of apoptosis. The apoptosis of U251 and LN229 cells was mediated by the anti-inflammatory effects of luteolin and associated activation of MAPKs (JNK, ERK, and p38) and death receptors (FADD) that regulated apoptotic proteins (caspase-8, caspase-3, and PARP). Further, luteolin promoted cell autophagy by upregulating LC3B II/I and downregulating p62 [126]. Moreover, luteolin reduced the expression of the STAT3 signaling pathway target gene Mcl-1, Survivin, and Bcl-xl—downregulation of which reduced inflammation in SGC7901, SGC7901/DDP, HGC27, MGC803, BGC803, and BGC823 gastric cancer cell lines. Inhibition of the STAT3 pathway after luteolin administration was mediated by the disruption of HSP90-STAT3 binding, which promoted its interaction with SHP-1. The anti-cancer efficacy of luteolin was confirmed in SGC7901, SGC7901/DDP, and HGC27 murine xenograft models [127]. Furthermore, luteolin and its derivative apigenin had a synergistic effect against H358 murine xenografts and Lewis lung carcinoma in vivo. Luteolin and apigenin inhibited lung cancer cell growth, induced apoptosis, and reduced IFN-γ-induced PD-L1 expression at inflammatory sites by suppressing STAT3 phosphorylation [128]. Due to its anti-inflammatory and antioxidant properties, apigenin also inhibited tumor promotion of HepG2 HCC through inhibited cell proliferation and induced apoptosis and autophagy via PI3K/AKT/mTOR pathway inhibition [129]. Moreover, apigenin inhibited the phosphorylation of the signaling molecules Lyn, Syk, phospholipase Cγ1, ERK, and JNK and the expression of the cytokines TNF-α, IL-4, IL-5, IL-6, IL-13, and COX-2—all of which induce inflammation and promote carcinogenesis—in RBL-2H3 rat leukemia cells [130]. Another study indicated that apigenin inhibited IL-6 expression and cell proliferation and promoted apoptosis by activating PARP and caspase-8 in Eca-109 and Kyse-30 human esophageal cancer cells [131].

### Plant extracts rich in flavonoids

Beyond isolated flavonoids, various plant extracts and flavonoids in whole plant foods exert beneficial effects against inflammation and associated tumor promotion. Co-administration of bilberry extracts and enzymatically modified isoquercitrin suppressed the promotion of HCC in PBO-promoted rats. This combination inhibited proliferation by reducing Ki67 and microsomal ROS levels. Bilberry extracts and isoquercitrin decreased p-PTEN, p-AKT, and Smad4 signaling—the downregulation of which was also connected with the inhibition of inflammation in PBO-promoted cases [132, 133]. Furthermore, Id1 is overexpressed in non-small-cell lung carcinoma and exerts pro-inflammatory and tumor-promoting effects. *Scutellaria* flavonoids, especially the three prominent representatives (baicalin, baicalein, and wogonin), inhibited Id1 through the activation of Rap1-GTP binding and the dephosphorylation of AKT and Src in A549 cells, H1299 non-small-cell lung carcinoma cells, and murine A549 xenografts [134]. In another study, baicalein and baicalin inhibited tumor promotion by downregulating PD-L1 and pro-inflammatory cytokine IFN-γ. These results were associated with STAT3 suppression in the SMMC-7721 and HepG2 human liver cancer cell lines [135]. Moreover, flavonoid-rich ethanol extracts of whole dried sugarcane reduced NF-κB phosphorylation and IL-8 secretion in SW480 colon cancer cells [136].

### Clinical studies

Only limited clinical studies are based on the consistent molecular mechanisms of flavonoids against inflammation in different tumor stages. A botanical drug called APG-157 containing multiple polyphenols suppressed tumor cells due to its antioxidant and anti-inflammatory properties, as demonstrated by reduced IL-1β, IL-6, and IL-8 concentrations in the salivary supernatant fluid of oral cancer patients [137]. Moreover, fisetin improved the inflammatory status of colorectal cancer patients by reducing IL-8 and hs-CRP levels [138]. Furthermore, consumption of green tea reduced NF-κB-associated inflammation in the radical prostatectomy tissue of men compared with black tea and water controls, suggesting that GTPs play a beneficial role in prostate cancer prevention and treatment [139]. Despite the known anti-inflammatory effects of soy in men with prostate cancer [140], in women with early-stage breast cancer with the potential of tumor promotion and progression, soy supplements rich in genistein and daidzein led to the overexpression of cell cycle transcripts, including those that promote cell proliferation such as FGFR2, E2F5, BUB1, CCNB2, MYBL2, CDK1, and CDC20 [141].

**Fig. 1:**
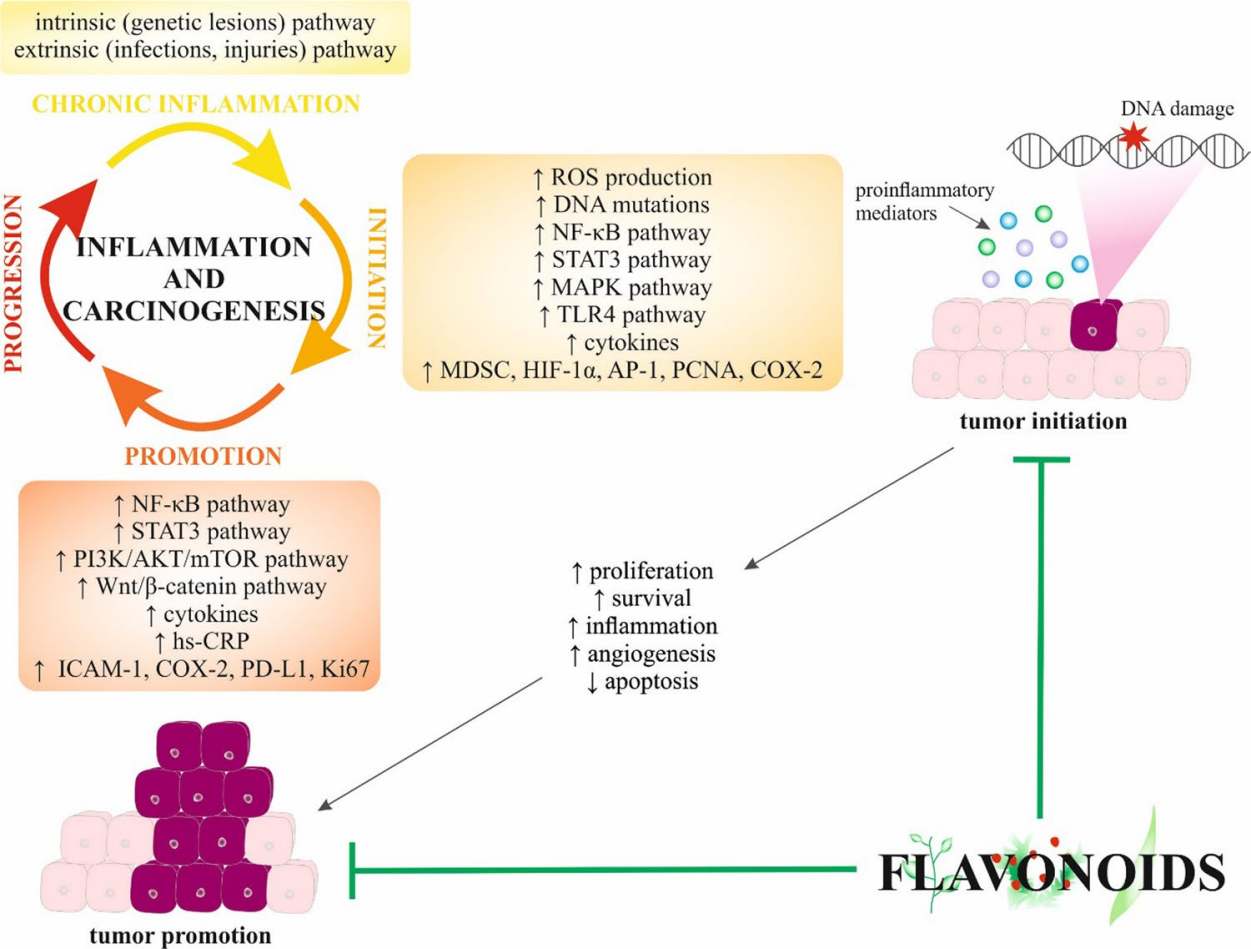
Suppression of tumor initiation and promotion by flavonoids. Abbreviations: ↑ increased/activated; ↓ decreased/inhibited

Flavonoids can suppress tumor promotion of various cancer types through diverse anti-inflammatory mechanisms. Therefore, the evaluation of molecular and cellular mechanisms of tumor-promoting inflammation is essential for developing preventive and anti-cancer therapies in the preclinical and clinical spheres. Discussed anti-inflammatory effects of flavonoids against tumor promotion are summarized in Table 2. Moreover, Fig. 1 depicts the efficacy of flavonoids in tumor initiation and promotion, and the affected signaling molecules and pathways.

**Table 2:** Suppression of tumor promotion by anti-inflammatory effects of flavonoids

Flavonoid	Cancer	Study design	Anti-inflammatory effects and/or mechanisms of tumor suppression	Ref
**Preclinical studies**				
Green tea polyphenols	Skin tumor	Six-week-old female SENCAR mice	↓ Stage I and stage II skin tumor promotion, ↓ skin papilloma formation, ↓ tumor multiplicity, ↓ tumor incidence, ↓ tumor growth	[118]
Silymarin			↓ Stage I and stage II skin tumor promotion, ↓ skin papilloma formation, ↓ tumor multiplicity, ↓ tumor incidence, ↓ tumor growth	[119]
Apigenin			↓ Skin papilloma formation, ↓ incidence of carcinomas/papillomas	[120]
Quercetin	Primary effusion lymphoma	BC3, BCBL1, and BC1 primary effusion lymphoma cells	↓ PI3K/AKT/mTOR, ↓ Wnt/β-catenin, ↓ STAT3, ↓ IL-6, ↓ IL-10, ↓ c-FLIP_L_, ↓ cyclin D1, ↓ cMyc	[121]
Icaritin	Chronic myeloid leukemia	K562 and primary chronic myeloid leukemia cells; 6–8-week-old female NOD-SCID nude mice	↓ Proliferation, ↓ tumor growth, ↑ apoptosis, regulation of MAPK/ERK/JNK, regulation of JAK2/STAT3/AKT, ↑ p-JNK, ↑ p-C-JUN, ↓ p-ERK, ↓ p-P38, ↓ JAK-2, ↓ p-JNK, ↓ p-STAT3, ↓ p-AKT	[122]
Fisetin	Lung cancer	IL-1β-promoted inflammatory responses of A549 lung adenocarcinoma cells	↓ ICAM-1, ↓ COX-2, ↓ PGE2, ↓ IL-8, ↓ CCL5, ↓ monocyte chemotactic protein 1, ↓ TNF- α, ↓ IL-6, ↓ NF-κB, ↓ ERK1/2	[123]
	Laryngeal carcinoma	TU212 head and neck squamous cell carcinoma cells; 6–8-week-old SPF male BALB/c nude mice	↑ Apoptosis, ↑ caspase-3, regulation of PI3K/AKT/NF-κB signaling, ↓ proliferation, ↓ tumor volume and weight, ↓ KI67, ↓ ERK1/2, ↓ PI3K/AKT-regulated mTOR	[124]
Fisetin + carnosic acid	Lung cancer	HCC827 and H358 human lung cancer cell lines; 8-week-old athymic nude mice injected with HCC827 and H358 cells	↑ Anti-inflammatory effects, ↑ apoptosis, ↑ caspase-3, ↑ Bax, ↑ Bad, ↓ Bcl-2, ↓ Bcl-xl, ↑ death receptor of TRAIL	[125]
Luteolin	Glioma	U251 and LN229 glioma cells	↓ Proliferation, ↑ apoptosis, ↑ MAPK, ↑ JNK, ↑ ERK, ↑ p38, ↑ FADD, ↑ caspase-8, ↑ caspase-3, ↑ PARP, ↑ autophagy, ↑ LC3B II, ↑ LC3B I, ↓ p62	[126]
	Gastric cancer	SGC7901, SGC7901/DDP, HGC27, MGC803, BGC803, and BGC823 gastric cancer cell lines; 6-week-male nude Balb/c SGC7901, SGC7901/DDP, and HGC27 murine xenografts	↓ STAT3, ↓ Mcl-1, ↓ Survivin, ↓ Bcl-xl, disruption of the binding of HSP-90 to STAT3, ↓ tumor growth	[127]
Luteolin + apigenin	Lung cancer	H358 murine xenografts and Lewis lung carcinoma in vivo model	↓ Lung cancer cell growth, ↑ apoptosis, ↓ IFN-γ-induced PD-L1 expression, ↓ STAT3 phosphorylation	[128]
Apigenin	HCC	HepG2 HCC	↓ Cell proliferation, ↑ apoptosis, ↑ autophagy, ↓ PI3K/AKT/mTOR pathway	[129]
	Leukemia	RBL-2H3 rat leukemia cells	↓ Lyn, ↓ Syk, ↓ phospholipase Cγ1, ↓ ERK, ↓ JNK, ↓ TNF-α, ↓ IL-4, ↓ IL-5, ↓ IL-6, ↓ IL-13, ↓ COX-2	[130]
	Esophageal cancer	Eca-109 and Kyse-30 human esophageal cancer cells	↓ IL-6, ↓ cell proliferation, ↑ apoptosis, ↑ PARP, ↑ caspase-8	[131]
Bilberry extracts + isoquercitrin	HCC	PBO-promoted rats	↓ Proliferation, ↓ Ki67, ↓ microsomal ROS, ↓ p-PTEN, ↓ p-AKT, ↓ Smad4,	[132]
*Scutellaria* flavonoids (baicalin, baicalein, and wogonin)	Non-small-cell lung carcinoma	A549 cells and H1299 non-small cell lung carcinoma cells; murine A549 xenografts (6-week-old male Balb/c thymic nude mice)	↓ Id1, ↑ Rap1-GTP binding, dephosphorylation of AKT and Src	[134]
Baicalein and baicalin	Liver cancer	SMMC-7721 and HepG2 human liver cancer cells	↓ PD-L1 expression, ↓ IFN-γ, ↓ STAT3 activity	[135]
Sugarcane	Colon cancer	SW480 colon cancer cells	↓ NF-κB phosphorylation, ↓ IL-8	[136]
**Clinical studies**				
APG-157	Oral cancer	A double-blind, randomized, placebo-controlled trial; normal subjects (*n* = 13) and patients with oral cancer (*n* = 12); two doses of APG-157 (100 mg or 200 mg) were delivered transorally every hour for 3 h	↑ Antioxidant activity, ↑ anti-inflammatory activity, ↓ IL-1β, ↓ IL-6, ↓ IL-8	[137]
Fisetin	Colorectal cancer	A double-blind, randomized placebo-controlled clinical trial, colorectal cancer patients (*n* = 37) undergoing chemotherapy were assigned to receive either 100 mg fisetin (*n* = 18) or placebo (*n* = 19) for 7 consecutive weeks	Improvement of inflammatory status, ↓ IL-8, ↓ hs-CRP	[138]
Green tea	Prostate cancer	Exploratory, open-label, phase II trial; men with prostate cancer (*n* = 113) were randomized to consume six cups daily of brewed green tea, black tea, or water (control) prior to radical prostatectomy	↓ NF-κB inflammatory pathway	[139]
Soy	Breast cancer	Randomized, placebo-controlled study; women with early-stage breast cancer (*n* = 140); soy protein supplementation (*n* = 70) or placebo (*n* = 70) for 7 to 30 days	↑ Cell cycle and proliferation, ↑ FGFR2, ↑ E2F5, ↑ BUB1, ↑ CCNB2, ↑ MYBL2, ↑ CDK1, ↑ CDC20	[141]

↑ increased/activated; ↓, decreased/inhibited

## Flavonoids as anti-inflammatory agents against tumor progression

As discussed above, cancer progression is a complex process related to a broad spectrum of cellular and molecular signals; however, current research, especially preclinical in vitro and in vivo analyses, increasingly supports the efficacy of naturally occurring flavonoids against inflammation-associated migration, invasion, and metastasis, which are significant inducers of cancer progression [53, 55, 78, 142, 143].

### Preclinical research

Biochanin A, an O-methylated isoflavone, inhibited the release of pro-inflammatory cytokines TNF-α and IL-6 from A427 lung cancer cells using the cocultured method. Moreover, biochanin A repressed the coculture-stimulated invasion of A427 cells accompanied by the modulation of EMT markers—downregulation of Snail and induction of E-cadherin. Indeed, due to the association between pro-inflammatory responses and tumor metastasis, the administration of biochanin A might alter the TME and thus hinder cancer migration and invasiveness [144]. Similarly, diosmetin isolated from *Dracocephalum peregrinum* L. repressed the migration and invasion of U251 glioma cells in vitro, as demonstrated by inhibition of the TGF-β signaling pathway and upregulation of E-cadherin [143]. Also, hesperetin suppressed NF-κB activation and reduced the secretion of pro-inflammatory TNF-α and IL-6 in HepG2 hepatic cancer cells; the anti-inflammatory effects of hesperetin are attributed to reduced ROS overproduction by the Nrf2 pathway [145].

Furthermore, EGCG suppressed the invasion, migration, and metastasis of Panc-1 and MIA PaCa-2 pancreatic cancer cells by the inhibition of the AKT pathway and modulation of EMT markers, specifically upregulated E-cadherin and downregulated N-cadherin and the mesenchymal markers TCF8/ZEB1, β-catenin, and vimentin [146]. Moreover, the novel synthetic flavonoid LFG-500 blocked the EMT and metastasis by downregulating YAP activity via ILK in MCF-7 breast cancer and A549 lung cancer cell models of TGF-β-induced EMT [76]. In addition, myricetin inhibited the invasion and migration of radioresistant A549-IR lung cancer cells, as demonstrated by the suppression of MMP-2 and MMP-9 (through FAK-ERK inhibition) and the EMT marker Slug [147]. Similarly, myricetin treatment potently inhibited the cytokine-induced migration and invasion of KKU-100 cholangiocarcinoma cells, mediated partially through STAT3 suppression; myricetin also suppressed downstream target genes of STAT3, including ICAM-1, MMP-9, iNOS, and COX-2 [148]. In addition, the newly synthesized flavonoid derivative GL-V9 suppressed the invasion and migration of HCT116 and SW480 colorectal cancer cells by inhibiting PI3K/AKT and MMP-2/9 signaling [149]. Moreover, baicalein showed anti-metastatic activity in breast cancer in vitro and in vivo demonstrated by the inhibition of STAT3 activity and suppressed IL-6 [150]. Also, chrysin and daidzein decreased CXCL1 and MMP-9, essential molecules released by the TME that facilitate tumor progression in the rat model of colorectal cancer; therefore, chrysin and daidzein have potential in preventing colorectal cancer angiogenesis and metastasis [151].

Moreover, chrysin inhibited the pro-inflammatory cytokine-induced EMT phenotype and CSC-like characteristics in HeLa cervical cancer cells by blocking the NF-κB/Twist axis [142]. Also, the citrus peel–derived flavonoid tangeretin inhibited breast CSC formation by suppressing STAT3 and reducing Sox2 levels [152]. In addition, the capacity of 8-bromo-7-methoxychrysin to reverse the M2 polarization of TAMs by inhibiting NF-κB highlights its potential to disrupt interactions between LCSLC and TAMs; indeed, M2 polarization of TAMs in the TME promotes the LCSLC capability of self-renewal [153].

Due to the association between inflammation and angiogenesis in tumor cells, Gong et al. (2018) evaluated the effects of flavonoids in the extract of *Scutellariae* Radix on inflammation-induced angiogenic responses. Eventually, *Scutellariae* Radix extract at various concentrations (0.03, 0.1, 0.3, 1.0 mg/mL) applied for 48 h decreased pro-inflammatory cytokines IL-1β, IL-6, and TNF-α, and suppressed the expression of the angiogenic biomarkers NF-κB, Cox-2, iNOS, and VEGF in LPS pre-treated cultured macrophage RAW 264.7 cells [154]. In addition, luteolin showed a potent capacity to target HIF-1α/VEGF signaling–mediated EMT and angiogenesis, as demonstrated by EMT suppression (increased E-cadherin and decreased N-cadherin and vimentin) and the downregulation of p-AKT, HIF-1α, VEGF-A, p-VEGFR-2, MMP-2, and MMP-9 in A375 and B16-F10 melanoma cells [155]. Moreover, EGCG and silibinin suppressed the migration of endothelial and lung tumor cells and downregulated VEGF, VEGFR2, and pro-angiogenic members of the miR-17-92 cluster [156].

**Fig. 2:**
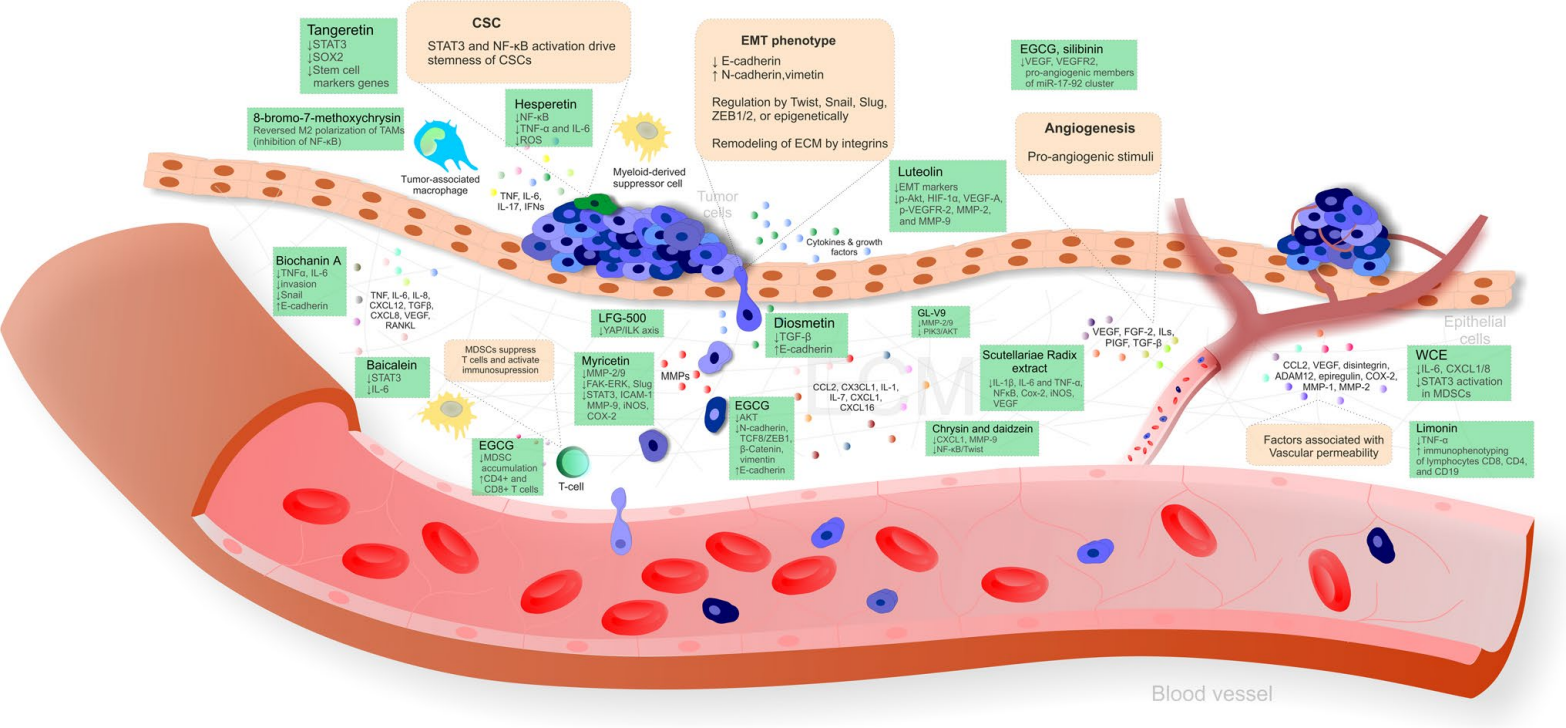
The effectiveness of flavonoids as anti-inflammatory agents against tumor progression. Abbreviations: ↑ increased/activated; ↓ decreased/inhibited

Also, WCE rich in flavonoids suppressed IL-6, CXCL1, and CXCL8 thus reducing tumor-elicited infiltration MDSCs, TAMs, and endothelial cells accompanied by reduced STAT3 activation, in MDSCs in PC-3 and DU145 prostate cancer xenografts. At the same time, these effects resulted in the inhibition of angiogenesis and metastasis [157]. Furthermore, EGCG attenuated immunosuppression in a 4T1 murine model of breast cancer by decreasing the accumulation of MDSCs and increasing CD4^+^ and CD8^+^ T cell numbers, suggesting that EGCG could effectively enhance the anti-tumor response [78]. Also, limonin decreased inflammation by reducing TNF-α and enhanced the adaptive immune response by promoting the immunophenotyping of CD8, CD4, and CD19 lymphocytes in a Balb/c murine model of colorectal carcinogenesis; limonin thus demonstrated immune-stimulating effects [158]. Figure 2 depicts the anti-inflammatory effects of naturally occurring flavonoids targeting the progression of cancer.

In conclusion, the results of current preclinical research highlight the effectiveness of flavonoids against inflammation-associated cancer progression (Table 3).

**Table 3:** Pre-clinical studies demonstrating high effectiveness of flavonoids as anti-inflammatory agents against tumor progression

Flavonoid	Study details	Mechanisms	Effect	Reference
Biochanin A	Coculture method—lung cancer A427 cell lines cocultured with monocytic leukemic AML-193 cells	Inhibited TNF-α and IL-6; repressed invasion; altered EMT markers (reduced Snail and increased E-cadherin)	Represses pro-inflammatory responses, invasion, migration, and metastasis	[144]
Diosmetin	Glioma U251 cells	Inhibited TGF-β; increased E-cadherin	Inhibited migration and invasion	[143]
Hesperetin	Hepatic cancer HepG2 cells	Suppressed NF-κB activation; reduced TNF-α and IL-6; reduced ROS overproduction by the Nrf2 pathway	Reduced inflammatory cytokine secretion	[145]
EGCG	Pancreatic cancer Panc-1 and MIA PaCa-2 cells	Inhibited AKT, modulated EMT markers (increased E-cadherin and decreased N-cadherin), decreased mesenchymal markers (TCF8/ZEB1, β-catenin, and vimentin)	Suppressed invasion, migration, and metastasis	[146]
LFG-500	TGF-β-induced model of EMT (breast cancer MCF-7 and lung cancer A549 cells)	Downregulation of YAP/ILK axis	Suppressed EMT and metastasis	[76]
Myricetin	Radioresistant lung cancer A549-IR cells	Suppressed MMP-2 and MMP-9 through the inhibition of FAK-ERK; decreased Slug	Inhibited invasion and migration	[147]
Myricetin	Cholangiocarcinoma KKU-100 cells	Suppressed STAT3 and its target genes ICAM-1, MMP-9, iNOS, and COX-2	Inhibited cytokine-induced migration and invasion	[148]
GL-V9	Colorectal cancer HCT116 and SW480 cells	Reduced MMP-2 and MMP-9; inhibited PI3K/AKT	Suppressed invasion and migration	[149]
Baicalein	Breast cancer model in vitro and in vivo	Inhibited STAT3 and IL-6	Reduced metastatic potential	[150]
Chrysin and daidzein	Colorectal cancer induced by subcutaneous injection of DMH in male albino rats	Decreased CXCL1 and MMP-9	Suppressed metastasis and angiogenesis	[151]
Chrysin	Cervical cancer HeLa cells co-administered with TNF-α and TGF-β	Blocked the NF-κB/Twist axis	Inhibited pro-inflammatory cytokine-induced EMT phenotype and CSC-like features	[142]
Tangeretin	Breast cancer MDA-MB-231 and MCF-7 cells	Suppressed STAT3 and reduced the level of Sox2; reduced transcript levels of stem cell marker genes (Oct3/4, Sox2, Nanog)	Inhibited breast CSCs	[152]
8-Bromo-7-methoxychrysin	LCSLC in vitro	Reversed M2 polarization of TAMs (inhibition of NF-κB)	Disrupted the interaction of LCSLCs and TAMs	[153]
*Scutellariae* Radix extract	LPS pre-treated cultured RAW 264.7 macrophages	Decreased IL-1β, IL-6, TNF-α, NF-κB, Cox-2, iNOS, and VEGF	Suppressed inflammation-induced angiogenesis	[154]
Luteolin	Melanoma A375 and B16-F10 cells	Modulated EMT markers (increased E-cadherin and decreased N-cadherin and vimentin); decreased p-AKT, HIF-1α, VEGF-A, p-VEGFR-2, MMP-2, and MMP-9	Suppressed HIF-1α/VEGF signaling–mediated EMT and angiogenesis (anti-metastatic effects)	[155]
EGCG, silibinin	HUVECs co-cultured with lung cancer A549 cells	Suppressed migration of endothelial and lung tumor cells; downregulated VEGF, VEGFR2, and pro-angiogenic members of the miR-17-92 cluster	Anti-angiogenic efficacy	[156]
WCE	Prostate cancer PC-3 and DU145 xenografts	Suppressed IL-6, CXCL1, and CXCL8 → reduced tumor-elicited infiltration of MDSCs, TAMs, and endothelial cells; inhibited STAT3 activation in MDSCs	Inhibited angiogenesis and metastasis	[157]
EGCG	Murine 4T1 model of breast cancer	Decreased accumulation of MDSCs; increased CD4^+^ and CD8^+^ T cells	Attenuated immunosuppression	[78]
Limonin	Balb/c model of colorectal carcinogenesis	Reduced TNF-α; promoted immunophenotyping of CD8, CD4, and CD19 lymphocytes	Decreased inflammation; enhanced immune responses	[158]

### Clinical studies

The above-discussed results of preclinical investigations support the need for further clinical evaluation of flavonoids to target the inflammation-induced progression of cancer. Indeed, Polyphenon E, which contains mainly EGCG and epicatechin, epigallocatechin, and epicatechin-3-gallate in lesser amounts, significantly reduced VEGF and HGF levels in a study of 26 men with positive prostate biopsies scheduled for radical prostatectomy [159]. Moreover, a randomized, phase II trial (conducted on 32 patients with prostate cancer) revealed that consuming bread enriched in soy isoflavones is associated with reduced pro-inflammatory cytokine levels and reduced ratios of T regulatory cells to CD8+ cells and MDSCs in peripheral blood. These results support the potential efficacy of flavonoids in promoting immune surveillance during cancer progression [140]. In addition, the positive effects of lifestyle modifications, including physical activity and diets rich in fruits and vegetables, were demonstrated by a clinical trial that revealed decreased TNF-α levels in BRCA1/2+ breast cancer survivors following a yearlong lifestyle modification program [160].

In conclusion, pre-clinical and clinical investigations point to the potential of flavonoids modulating all stages of the complex process of carcinogenesis associated with inflammation. Obtaining data to enable the implementation of agents precisely targeting specific stages of carcinogenesis will allow the utilization of predictive, preventive, and personalized approaches that increase efficiency and decrease cancer management costs.

## Exemplified 3PM pathways

As detailed above, non-physiologic inflammation may initiate carcinogenesis and evidently contribute to tumor development and progression as well as it deteriorates individual outcomes in the cohort of cancer patients [161]. The good news is that, due to their evident anti-inflammatory properties, flavonoids are clinically relevant candidates as preventive and therapeutic agents to improve individual outcomes in diseases linked to the non-physiologic inflammation. The challenge is, however, to diagnose well in time an individual predisposition to non-physiologic inflammatory processes such as low-grade chronic inflammation that is definitely the task for predictive diagnostics tools and targeted individualized prevention. Clinical translation is a process of application of preclinical observations and results after clinical validation into medical practice. All described flavonoids targeting specific signaling cascades associated with inflammation are components of different plants, and their synergic/additive effects are associated with supporting human health. The implication of 3PM as a concept of medicine of the twenty-first century into routine clinical practice is, among other things, based on increasing the amount of flavonoid intake from food due to their beneficial aspect in the prevention of various pathologies. Table 4 links the previously referred preclinical research on some flavonoids to the practical amount of particular food in the daily diet.

**Table 4:** Content of flavonoids in foods

Flavonoids	Food source	Amounts of flavonoids in food mg/100g	References
(-)-Epicatechin	Apples (raw, with skin)	15.12	[162]
	Peaches (raw)	12.24	[162]
	Cranberries (raw)	25.93	[162]
	Cocoa (dry powder)	183.49	[162]
	Red wine (table)	20.49	[162]
Myricetin	Blueberries	1.26	[162]
	Garlic	1.61	[162]
	Red wine	0.83	[163]
Biochanin A	Peanut	0.06	[164]
Baicalein	Welsh onion	1.80e-03	[165]
Quercetin-3-O-glucoside	Onions	21.40	[162]
	Kale	22.58	[162]
Daidzein	Tempeh	22.66	[166]
Tangeretin	Orange juice	0.3	[167]
	Soybean	61.33	[166]
Genistein	Tofu	12.99	[166]
	Tempeh	36.15	[166]
	Soy milk	4.94	[168]
Diosmetin 7-O-rutinoside	Lemon—pure juice	2.92	[169]
	Peppermint	95.50	[169]
Hesperetin	Lemon juice	14.47	[162]
	Orange juice	20.39	[162]
Apigenin	Spices, celery seed	83.70	[162]
	Peppermint	8.71	[162]
Luteolin	Green peppers	4.71	[162]
	Olive oil	0.36	[170]
	Pistachio	0.10	[170]

To this end, individualized profiling based on specialized survey and specific multi-diagnostic patterns has been demonstrated as instrumental for primary, secondary, and tertiary healthcare exemplified below.

A. Primary healthcare: prediction of relevant suboptimal health conditions such as family (genetic) predisposition, abnormal BMI (both overweight and underweight), abnormal stress reactions, disturbed microcirculation, and delayed healing, among others [171–175]; based on anti-inflammatory properties of flavonoids, corresponding individualized mitigating measures may be considered as follows. Healthy and balanced diet rich in naturally occurring phytochemicals including flavonoids is essential to prevent non-communicable diseases associated with non-physiologic inflammation [55, 176, 177]. In particular, flavonoids show significant efficacy to maintain optimal weight, including less likeliness to be obese [176, 178, 179]. Preclinical research elaborate the effectiveness of flavonoids to prevent inflammation associated with obesity [180, 181]; moreover, these results are supported by clinical studies conducted on specific parts of the population evaluating individual health conditions. Indeed, a randomized clinical trial by Lee et al. (2016) demonstrated the efficacy of anthocyanin-rich black soybeans to reduce abdominal fat and lipid profiles in overweight or obese paticipants [182]. Moreover, anthocyanin exerted beneficial metabolic effects demonstrated through the prevention of insuline resistance in subjects with type 2 diabetes [183]; besides, current evidence highlights the association between cancer and insulin resistance, which is common in obese individuals and type 2 diabetes [184]. Also, flavonoids are efficient agents mitigating chronic stress and improving overall brain health [185, 186]. Cocoa products were found to induce anti-inflammatory effects demonstrated through decreased IL-10 and IL-1β in healthy and hypercholesterolaeic individuals [187]. Similarly, results of a double-blinded randomized trial showed the association of high-polyphenol chocolate and increased ICAM-1 in type 2 diabetes when compared with control [188]. The potent capacity of flavonoids in the primary healthcare mediated through multiple mechanisms of action including the anti-inflammatory capacity is demonstrated also by the maintenance of cardiovascular health [189, 190] or wound healing [191, 192]. Clinical evidence demonstrate honey, a rich source of flavonoids [193], to exert efficacy during healing of wounds and ulcers that had failed to heal using conventional approaches [194]; thus, honey possesses the ability to resolve the inflammatory state of chronic wounds [195]. Also, clinical and epidemiological data support the notion of effectiveness of flavonoids to prevent conditions associated with increased risk of malignant diseases [89, 196–198]. Indeed, soy isoflavone was found to increase serum IL-6 in postmenopausal women and thus enhancing the immune surveillance associated with lower incidence of cancer in parts of the world characterized by higher soy intake [199]. Furthermore, mediterranean diet exerted potential beneficiary effects in primary breast cancer prevention [200] preventing all breast cancer subtypes [201], presumably through various anti-cancer mechanisms including anti-inflammatory activity. Therefore, due to their anti-inflammatory capacity, naturally occurring flavonoids and flavonoid-rich plant food represent potent agents for primary healthcare of suboptimal health conditions and/or specific disease predisposition associated with the risk of cancer .
B. Secondary healthcare: prediction of tumor progression and increased risk of pre-metastatic niches/metastatic disease [4, 202, 203]; as mentioned above, flavonoids were comprehensively documented to prevent the onset of the cancer invasiveness in preclinical research via the modulation of numerous signaling pathways involved in critical steps of metastatic spread. In addition, flavonoids were described to be effective oncostatic substances in highly aggressive cancer models including various in vivo approaches. Regarding oncology practice, flavonoids demonstrated promising results applied in the combination with conventional chemotherapeutics in metastatic cancer disease. However, in-depth analyses of re-sensitizing cancer cells by flavonoids towards conventional chemotherapy and assessing the activities of flavonoids on cancer stem cells survival, affecting the relapse and multidrug resistance, are needed [55].
  There are only limited clinical data evaluating the effectiveness of flavonoids against advanced cancer disease. Curcumin has been documented to suppress cancer cells due to its anti-inflammatory and antioxidant effects. On the other hand, its effectiveness is limited by poor absorption after oral administration. A botanical drug APG-157 containing multiple polyphenols, including flavonoids, demonstrated improved bioavailability and clinical activity in patients with oral cancer. APG-157 was well absorbed, reduced parameters of inflammation, and upregulated expression of genes linked with differentiation and T cell recruitment to the TME. These data shows the potential using of APG-157 in combination with anti-cancer therapies including advanced disease [137]. In another clinical study, fisetin reduced the plasma levels of IL-8, hs-CRP, and MMP-7 levels (*p* < 0.02) [138]. Green tea and its constituents, mainly EGCG, show anti-inflammatory activities associated with reduced VEGF and HGF levels in prostate cancer patients [139, 159]. Oral administration of soy isoflavone–enriched bread significantly suppressed proinflammatory cytokines and immunosuppressive cells in men with prostate cancer [140]. Finally, lifestyle modifications in BRCA1/2+ breast cancer survivors, including physical activity and diets rich in flavonoids (in fruits and vegetables), revealed decreased TNF-α levels [160]. All these mentioned clinical data suggest anti-metastatic potential of fisetin, green tea, soy, and fruit and vegetrables rich in flavonoids in cancer patients. Based on preclinical research, there are numerous studies demonstrating high effectiveness of flavonoids and flavonoid-rich extracts as anti-inflammatory agents against tumor progression. These include biochanin A, diosmetin, hesperetin, EGCG, synthetic flavonoids LFG-500 and GL-V9, myricetin, baicalein, chrysin, daidzein, tangeretin, 8-bromo-7-methoxychrysin, *Scutellariae* Radix extract, luteolin, silibinin, WCE, and limonin (see Table 3).
C. Tertiary healthcare: prediction in palliative care [204]; in this regard, flavonoids were described to block an activation of numerous cellular regulatory proteins such as cytokines and transcription factors involved in cellular inflammatory responses and pain. From the clinical point of view, it could be very beneficial to develop protective delivery formulations containing flavonoids to treat inflammation and pain. Flavonoids suppress the expression of wide spectrum of inflammatory molecules such as NO, TNF-α, IL-1β, and COX-2; downregulate ICAM-1 and VEGF synthesis; and, moreover, activate STAT3, NF-kB, NLRP3 inflammasome, and finally MAPK cellular pathways. Based on mentioned multi-target activities of flavonoids, they have great potential in clinical sphere, including oncology practice, due to their anti-inflammatory and analgesic properties [205].
  There are several examples of specific flavonoids that should be beneficial in palliative care in cancer patients. In the case report, the breast cancer patient showed progressive liver failure despite several chemotherapy treatments, including paclitaxel, capecitabine, and vinorelbine. Silibinin application improved hepatic failure due to extensive liver infiltration in this patient. After the initiation of therapy, the patient showed clinical and liver improvements that permitted the continuation of palliative chemotherapy [206]. Harati et al. (2017) documented that EGCG and silibinin represent potential candidate molecules as mild therapeutic options for patients with solid sarcomas that require palliative treatment and are not suitable for doxorubicin-based chemotherapy [207]. In another preclinical study, baicalelin and 6-gingerol reduced 5-fluorouracil-induced overexpression of CXCL1 in the colon and prevented the development of neutrophil recruitment and weakened diarrhea development by the suppression of NF-κB activity [208]. Data from another preclinical study point out to protective role of quercetin coadministered with vitamin E in the prevention of doxorubicin-induced toxicity in uterine and ovarian tissues in rats [209]. The administration of casticin in male rats demonstrated a palliative effects against cisplatin-induced renal damage and recovered all renal parameters to normal levels [210].
  Besides cancer, there are also other life-threatening disease/condition  in which flavonoids show positive palliative effects. Using mouse model, EGCG significantly reduced osteoarthritis disease progression and exerts palliative effects [211]. Kaswan et al. (2021) described that the serotonergic pathway (via the 5-HT1A receptor subtype in the central nervous system) is essential for cardamonin to suppress neuropathic pain in chronic constriction injury–induced neuropathic pain animal model [212]. EGCG administration modulating the Wnt/β-catenin signaling pathways reduced postoperative pain related to inflammatory and neurochemical alterations [213]. Most of the above data are from preclinical research; therefore, well-controlled clinical studies are needed to validate the positive effects of flavonoids in palliative care in patients with cancer as well as other diseases.

## Strengths and limitations

Phytochemicals represent naturally occuring anti-cancer agents affecting numerous cellular pathways. However, the utilization of flavonoids in clinical practice to prevent or treat cancer still faces difficulties associated with limitations of studies performed. The efficacy of flavonoids as anti-inflammatory agents in vivo is  strongly dependent on its bioavailability affected by various factors on the side of the individual recipient or properties of the flavonoid itself including low absorption, an extensive metabolization, rapid elimination, or structural complexity of flavonoids within subclasses [55]. For example, catechins appear to be absorbed in amount smaller than intake due to their rapid metabolization [214]. Therefore, an increase in the bioavailablity of flavonoids envisaged by current research [215]. To this end, complexity and chronicity as basic characteristcs of plenty of human diseases as well as sex, age, comorbidities, genetic similarity, and environmental factors should be carefully considered and appropriately modeled in research and individualised application of flavonoids [216].

The abovementioned limitations associated with the evaluation of anti-cancer effects of phytochemicals in vitro and in vivo are, further, exemplified below. Preclinical in vitro studies demonstrate potent capacity of soy isoflavones to suppress prostate carcinogenesis [217]. However, as stated by Miltyk et al. (2003), despite the capacity of isoflavone to induce genetic damage of prostate cancer cells in vitro, similar effects were not observed in human subjects [218]. Similarly, isoflavones exerted no effects on markers of inflammation [219] and had no effect on prostate-specific antigen or hormone levels in prostate cancer patients [220]. However, the evaluation of other doses and duration of the administration in further research of anti-cancer efficacy of isoflavones in prostate cancer might be beneficial for the data interpretation [219].

The preventive efficacy of EGCG was supported by the detection of methylated and nonmethylated forms of EGCG in prostate tissue after short-term green tea intervention [221]. Further, potent capacity of a mixture of natural agents to protect human lymphocytes in vitro in comparison with single agents [222] was further emphasized by a proof-of-concept study in humans in vivo [223]. Phytochemicals, for example tannins, chelate metal ions that generate ROS and thus stabilize potential pro-oxidant activity [224]. , In conclusion, to overcome persisting limitations in the field, intensified  preclinical [225] and clinical research [159, 225]  on phytochemicals is essential to be performed in the framework of predictive, preventive and personalized medicine. 

## Supplementary Information

ESM 1 (DOC 16077 kb)

## Data Availability

Not applicable.

## Abbreviations

4EBP1: Translation repressor protein
ACAN: Aggrecan
ADAM: Metalloproteinase domain-containing protein 12
ADAMTS: Disintegrin and metalloproteinase with thrombospondin motifs
AKT: Serine/threonine-*protein* kinase/*protein* kinase B
AMPK: 5′ AMP-activated protein kinase
AOM: Azoxymethane
AP-1: Activator protein 1
APR: Acute phase response
Arg-1: Arginase 1
Bad: Bcl-2-associated agonist of cell death
BALP: Bone alkaline phosphatase
Bax: Bcl-2-associated X protein
Bcl-2: B cell lymphoma 2
BMI: Body mass index
BM-MSC: Bone marrow mesenchymal stem cells
BUB1: Mitotic checkpoint serine/threonine kinase
CAFs: Cancer-associated fibroblasts
CAT: Catalase
CCL: C-C motif chemokine ligand
CCNB2: Cyclin B2
CD: Cluster of differentiation
CDC20: Cell division cycle 20
CDK1: *Cyclin-dependent kinase 1*
c-FLIP_L_: FADD-like interleukin-1 beta-converting enzyme inhibitory protein
CINC-1: Cytokine-induced neutrophil chemoattractant-1
cMYC: Regulator gene and proto-oncogene
COL2A1: Collagen type II alpha 1 chain
COX-2: Cyclooxygenase 2
CSCs: Cancer stem cells
CSE: Cigarette smoke extracts
CTX-II: C-terminal cross-linked telopeptide of type *II* collagen
CX3CL1: C-X3-C motif chemokine ligand 1
CXCL: C-X-C motif chemokine ligand
CXCR4: C-X-C motif chemokine receptor 4
DMBA: 7,12-Dimethylbenz(a)anthracene
DMH: 1,2-Dimethylhydrazine dihydrochloride
DSS: Dextran sulfate sodium
E2F5: E2F transcription factor 5
ECM: Extracellular matrix
EGCG: Epigallocatechin-3-gallate
eIF4E: Eukaryotic translation initiation factor 4E
EMT: Epithelial–mesenchymal transition
eNOS: Endothelial *nitric oxide synthase*
ERK: Extracellular signal-regulated kinase
ETV6-RUNX1: ETS variant transcription factor 6-runt-related transcription factor 1
FAK: Focal adhesion kinase
FGF2: Fibroblast growth factor 2
FGFR2: *Fibroblast growth factor receptors*
G-CSF: Granulocyte colony–stimulating factor
GM-CSF: Granulocyte-macrophage colony–stimulating factor
GSH: Glutathione
GTPs: Green tea polyphenols
HCC: Hepatocellular carcinoma
HGF: Hepatocyte growth factor
HIF-1α: Hypoxia-inducible factor 1α
Hs-CRP: High-sensitivity C-reactive protein
HSP90: Heat shock protein 90
IBD: Inflammatory bowel disease
ICAM-1: Intercellular adhesion molecule 1
ID1: Inhibitor of differentiation 1
IFN-γ: *Interferon gamma*
IGF-1/IGF-1R: Insulin-like growth factor *1/*insulin-like growth factor type *1 receptor*
IKK: Kappa kinase
IL: Interleukin
ILK: Integrin-linked protein kinase
iNOS: *Inducible nitric oxide synthase*
JAK: Janus kinase
JNK: c-Jun amino-terminal kinases
Ki67: Proliferative index
LC3B: Autophagy marker light chain
LCSLC: Liver cancer stem–like cells
LIF: Leukemia inhibitory factor
LPS: Lipopolysaccharide
Lyn: *LYN* proto-oncogene, Src family tyrosine kinase
MAMs: Metastasis-associated macrophages
MAPK: Mitogen-activated protein kinase
Mcl-1: *MCL1* apoptosis regulator, Bcl-2 family member
MCP-1: *Monocyte chemoattractant protein-1*
M-CSF: Macrophage colony–stimulating factor
MDA: Malondialdehyde
MDSCs: Myeloid-derived suppressor cells
MMPs: Matrix metalloproteinases
MPG: N-Methylpurine DNA *glycosylase*
mTOR: *Mechanistic target of rapamycin*
MYBL2: MYB proto-oncogene like 2
NADPH: Nicotinamide adenine dinucleotide phosphate
NF-κB: Nuclear factor kappa-light-chain-enhancer of activated B cells
NLRP3: NOD-, LRR-, and pyrin domain–containing protein 3
NOX2: NADPH oxidase 2
Nrf2: Nuclear factor erythroid 2–related factor 2
OC: Osteocalcin
OPG: Osteoprotegerin
PARP: Poly (ADP-ribose) polymerase
PBO: Piperonyl butoxide
PCNA: Proliferating cell nuclear antigen
PD-L1: Programmed death-ligand 1
PGE2: Prostaglandin E2
PI3K: Phosphoinositide 3-kinase
PIGF: Placental growth factor
PIINP: Human procollagen II N-terminal propeptide
PIM2: Pim-2 proto-oncogene, serine/threonine kinase
PPPM / 3PM: Predictive, preventive, and personalized medicine
PTEN: Phosphatase and tensin homolog
PTGER2: Prostaglandin E receptor 2
PTGS2: Prostaglandin-endoperoxide synthase 2
RANKL: Receptor activator of nuclear factor-κB ligand
Rap1: Ras-related protein 1
ROS/RNS: Reactive oxygen species/reactive nitrogen species
S100A7/RAGE: Receptor for advanced glycation end products
S6K1: Ribosomal protein S6 kinase beta-1
SAA: Serum amyloid A
SHP-1: Src homology region 2 domain–containing phosphatase 1
Slug: SNAI2 *snail family transcriptional repressor 2*
Smad4: SMAD family member 4
Snail: Zinc finger protein SNAI1
SOD: Superoxide dismutase
Sox2: SRY-box transcription factor 2
Src: Proto-oncogene, tyrosine-protein kinase
STAT3: Signal transducer and activator of transcription 3
Syk: Spleen-associated tyrosine kinase
TAMs: Tumor-associated macrophages
TANs: Tumor-associated neutrophils
TGF-β: *Transforming growth factor* beta
Th17s: T helper IL-17-producing cells
TIMP1: TIMP metallopeptidase inhibitor 1
TLR4: Toll-like receptor 4
TME: Tumor microenvironment
TNF-α: Tumor necrosis factor alpha
TPA: 12-O-Tetradecanoylphorbol-13-acetate
TRAIL: TNF-related apoptosis-inducing ligand
Tregs: T-regulatory cells
Twist: *Twist* family BHLH transcription factor 1
UV: Ultraviolet
VCAM-1: Vascular cell adhesion molecule 1
VEGF: *Vascular endothelial growth factor*
VEGFR2: Vascular endothelial growth factor receptor 2
WCE: *Wedelia chinensis* herbal extract
YAP: Yes1-associated transcriptional regulator
ZEB1: Zinc finger E-box binding homeobox 1
