# Supplementary material for: Flavonoids against non-physiologic inflammation attributed to cancer initiation, development, and progression—3PM pathways
Source: EPMA J. 2021 Oct 6;12(4):559–87. doi: 10.1007/s13167-021-00257-y (PMC8648878; doi:10.1007/s13167-021-00257-y)
Supplement: Supplementary file 1 — (DOC 16077 kb) [file 13167_2021_257_MOESM1_ESM.doc]

**Flavonoids against non-physiologic inflammation attributed to cancer initiation, development and progression – 3PM pathways**

Peter Kubatka^1^*, Alena Mazurakova^2^, Marek Samec^3^, Lenka Koklesova^2^, Kevin Zhai^4^, Raghad AL-Ishaq^4^, Karol Kajo^5^, Kamil Biringer^2^, Desanka Vybohova^6^, Aranka Brockmueller^7^, Martin Pec^1^, Mehdi Shakibaei^7^, Frank A. Giordano^8^, Dietrich Büsselberg^4^,

Olga Golubnitschaja^9^*

^1^ Department of Medical Biology, Jessenius Faculty of Medicine, Comenius University in Bratislava, 03601 Martin, Slovakia. [peter.kubatka@uniba.sk](mailto:peter.kubatka@uniba.sk), [martin.pec@uniba.sk](mailto:martin.pec@uniba.sk)

^2^ Department of Obstetrics and Gynecology, Jessenius Faculty of Medicine, Comenius University in Bratislava, 03601 Martin, Slovakia. [liskova80@uniba.sk](mailto:liskova80@uniba.sk), [marek.samec@uniba.sk](mailto:marek.samec@uniba.sk), [koklesova5@uniba.sk](mailto:koklesova5@uniba.sk), [kamil.biringer@uniba.sk](mailto:kamil.biringe@uniba.sk)

^3^ Biomedical Centre Martin, Jessenius Faculty of Medicine in Martin, Comenius University in Bratislava, Mala Hora 4D, 036 01 Martin, Slovakia

^4^ Weill Cornell Medicine-Qatar, Education City, Qatar Foundation, Doha, 24144 Qatar. [kez4003@qatar.med.cornell.edu](mailto:kez4003@qatar.med.cornell.edu), [rkmalishaq@hotmail.com](mailto:rkmalishaq@hotmail.com), [dib2015@qatar-med.cornell.edu](mailto:dib2015@qatar-med.cornell.edu)

^5^ Department of Pathology, St. Elizabeth Cancer Institute Hospital, 81250 Bratislava, Slovakia. [kkajo@ousa.sk](mailto:kkajo@ousa.sk)

^6^ Department of Anatomy, Jessenius Faculty of Medicine, Comenius University in Bratislava, 03601 Martin, Slovakia. [desanka.vybohova@uniba.sk](mailto:desanka.vybohova@uniba.sk)

^7^ Musculoskeletal Research Group and Tumor Biology, Chair of Vegetative Anatomy, Faculty of Medicine, Institute of Anatomy, Ludwig-Maximilian-University Munich, Munich, Germany. [Aranka.Brockmueller@med.uni-muenchen.de](mailto:Aranka.Brockmueller@med.uni-muenchen.de), [mehdi.shakibaei@med.uni-muenchen.de](mailto:mehdi.shakibaei@med.uni-muenchen.de)

^8^ Department of Radiation Oncology, University Hospital Bonn, Rheinische Friedrich-Wilhelms-Universität Bonn, Germany. frank.giordano@[ukbonn.de](http://ukbonn.de/)

^9^ Predictive, Preventive and Personalised (3P) Medicine, Department of Radiation Oncology, University Hospital Bonn, Rheinische Friedrich-Wilhelms-Universität Bonn, Bonn, Germany. [olga.golubnitschaja@ukbonn.de](mailto:olga.golubnitschaja@ukbonn.de)

**Corresponding authors*:** Peter Kubatka, [peter.kubatka@uniba.sk](mailto:peter.kubatka@uniba.sk); Olga Golubnitschaja, [olga.golubnitschaja@ukbonn.de](mailto:olga.golubnitschaja@ukbonn.de)

**Keywords:** natural substances; phytochemicals; flavonoids; ROS; DNA damage; carcinogenesis; inflammation; low-grade systemic inflammation; cancer; cancer initiation; cancer promotion; cancer progression; systemic hypoxic-ishemic effects; pre-metastatic niches; impaired healing; molecular targets; NF-κB signaling; inflammatory pathway; HIF-1α; TNF-α; cytokines; IL-1β; IL-6; IL-8; S100; tissue remodelling; matrix metalloproteinases; predictive preventive personalized medicine (PPPM/3PM)

**Abstract**

Inflammation is an essential pillar of the immune defense. On the other hand, chronic inflammation is considered a hallmark of cancer initiation and progression. Chronic inflammation demonstrates a potential to induce complex changes at molecular, cellular, and organ levels including but not restricted to the stagnation and impairment of healing processes, uncontrolled production of aggressive ROS/RNS, triggered DNA mutations and damage, compromised efficacy of the DNA repair machinery, significantly up-regulated cytokine/chemokine release and associated patho-physiologic protein synthesis, activated signaling pathways involved in carcinogenesis and tumor progression, abnormal tissue remodeling, and created pre-metastatic niches, among others.

The anti-inflammatory activities of flavonoids demonstrate clinically relevant potential as preventive and therapeutic agents to improve individual outcomes in diseases linked to the low-grade and chronic inflammation, including cancers. To this end, flavonoids are potent modulators of pro-inflammatory gene expression being therefore, of great interest as agents selectively suppressing molecular targets within pro/inflammatory pathways.

This paper provide in-depth analysis of anti-inflammatory properties of flavonoids, highlights corresponding mechanisms and targeted molecular pathways, and proposes potential treatment models for multi-level cancer prevention in the framework of predictive, preventive and personalized medicine (PPPM / 3PM). To this end, individualized profiling and patient stratification are essential for implementing targeted anti-inflammatory approaches. Most prominent examples are presented for the proposed application of flavonoids-conducted anti-inflammatory treatment in overall cancer management.

**Abbreviations:**

4EBP1 translation repressor protein

ACAN aggrecan

ADAM metalloproteinase domain-containing protein 12

ADAMTS disintegrin and metalloproteinase with thrombospondin motifs

AKT serine/threonine-protein kinase/protein kinase B

AMPK 5' AMP-activated protein kinase

AOM azoxymethane

AP-1 activator protein 1

APR acute phase response

Arg-1 arginase 1

Bad Bcl-2 associated agonist of cell death

BALP bone alkaline phosphatase

Bax Bcl-2-associated X protein

Bcl-2 B-cell lymphoma 2

BMI body mass index

BM-MSC bone marrow mesenchymal stem cells

BUB1 mitotic checkpoint serine/threonine kinase

CAFs cancer-associated fibroblasts

CAT catalase

CCL C-C motif chemokine ligand

CCNB2 cyclin B2

CD cluster of differentiation

CDC20 cell division cycle 20

CDK1 cyclin-dependent kinase 1

c-FLIP_L_ FADD-like interleukin-1 beta-converting enzyme inhibitory protein

CINC-1 cytokine-induced neutrophil chemoattractant-1

cMYC regulator gene and proto-oncogene

COL2A1 collagen type II alpha 1 chain

COX-2 cyclooxygenase 2

CSCs cancer stem cells

CSE cigarette smoke extracts

CTX-II C-terminal cross-linked telopeptide of type II collagen

CX3CL1 C-X3-C motif chemokine ligand 1

CXCL C-X-C motif chemokine ligand

CXCR4 C-X-C motif chemokine receptor 4

DMBA 7,12-dimethylbenz(a)anthracene

DMH 1, 2-dimethylhydrazine dihydrochloride

DSS dextran sulfate sodium

E2F5 E2F transcription factor 5

ECM extracellular matrix

EGCG epigallocatechin-3-gallate

eIF4E eukaryotic translation initiation factor 4E

EMT epithelial–mesenchymal transition

eNOS endothelial nitric oxide synthase

ERK extracellular signal-regulated kinase

ETV6-RUNX1 ETS variant transcription factor 6-runt-related transcription factor 1

FAK focal adhesion kinase

FGF2 fibroblast growth factor 2

FGFR2 fibroblast growth factor receptors

G-CSF granulocyte colony stimulating factor

GM-CSF granulocyte-macrophage colony-stimulating factor

GSH glutathione

GTPs green tea polyphenols

HCC hepatocellular carcinoma

HGF hepatocyte growth factor

HIF-1α hypoxia-inducible factor 1α

Hs-CRP high-sensitivity C-reactive protein

HSP90 heat shock protein 90

IBD inflammatory bowel disease

ICAM-1 intercellular adhesion molecule 1

ID1 inhibitor of differentiation 1

IFN-γ interferon gamma

IGF-1/IGF-1R insulin-like growth factor 1/insulin-like growth factor type 1 receptor

IKK kappa kinase

IL interleukin

ILK integrin-linked protein kinase

iNOS inducible nitric oxide synthase

JAK Janus kinase

JNK c-Jun amino-terminal kinases

Ki67 proliferative index

LC3B autophagy marker light chain

LCSLC liver cancer stem-like cells

LIF leukemia inhibitory factor

LPS lipopolysaccharide

Lyn LYN proto-oncogene, Src family tyrosine kinase

MAMs metastasis-associated macrophages

MAPK mitogen-activated protein kinase

Mcl-1 MCL1 apoptosis regulator, Bcl-2 family member

MCP-1 monocyte chemoattractant protein*-*1

M-CSF macrophage colony-stimulating factor

MDA malondialdehyde

MDSCs myeloid-derived suppressor cells

MMPs matrix metalloproteinases

MPG N-methylpurine DNA glycosylase

mTOR mechanistic target of rapamycin

MYBL2 MYB proto-oncogene like 2

NADPH nicotinamide adenine dinucleotide phosphate

NF-κB nuclear factor kappa-light-chain-enhancer of activated B cells

NLRP3 NOD-, LRR- and pyrin domain-containing protein 3

NOX2 NADPH oxidase 2

Nrf2 nuclear factor erythroid 2–related factor 2

OC osteocalcin

OPG osteoprotegerin

PARP poly (ADP-ribose) polymerase

PBO piperonyl butoxide

PCNA proliferating cell nuclear antigen

PD-L1 programmed death-ligand 1

PGE2 prostaglandin E2

PI3K phosphoinositide 3-kinase

PIGF placental growth factor

PIINP human procollagen II N-terminal propeptide

PIM2 Pim-2 proto-oncogene, serine/threonine kinase

PPPM / 3PM predictive, preventive and personalized medicine

PTEN phosphatase and tensin homolog

PTGER2 prostaglandin E receptor 2

PTGS2 prostaglandin-endoperoxide synthase 2

RANKL receptor activator of nuclear factor-κB ligand

Rap1 Ras-related protein 1

ROS/RNS reactive oxygen species/reactive nitrogen species

S100A7/RAGE receptor for advanced glycation end products

S6K1 ribosomal protein S6 kinase beta-1

SAA serum amyloid A

SHP-1 Src homology region 2 domain-containing phosphatase 1

Slug SNAI2 snail family transcriptional repressor 2

Smad4 SMAD family member 4

Snail zinc finger protein SNAI1

SOD superoxide dismutase

Sox2 SRY-box transcription factor 2

Src proto-oncogene, tyrosine-protein kinase

STAT3 signal transducer and activator of transcription 3

Syk spleen associated tyrosine kinase

TAMs tumor-associated macrophages

TANs tumor-associated neutrophils

TGF-β transforming growth factor beta

Th17s T helper IL-17-producing cells

TIMP1 TIMP metallopeptidase inhibitor 1

TLR4 toll-like receptor 4

TME tumor microenvironment

TNF-α tumor necrosis factor alpha

TPA 12-O-tetradecanoylphorbol-13-acetate

TRAIL TNF-related apoptosis-inducing ligand

Tregs T-regulatory cells

Twist Twist family BHLH transcription factor 1

UV ultraviolet

VCAM-1 vascular cell adhesion molecule 1

VEGF vascular endothelial growth factor

VEGFR2 vascular endothelial growth factor receptor 2

WCE *Wedelia chinensis* herbal extract

YAP Yes1 associated transcriptional regulator

ZEB1 zinc finger E-box binding homeobox 1

**Introduction**

Inflammation is a physiological process necessary for homeostasis. Inflammation plays an elementary role in host defense against various pathogens through the activation and recruitment of immune cells and the consequent actions of innate and adaptive immunity. It is imperative for processes such as tissue regeneration, repair, and remodelling. Furthermore, its subtle manifestations are essential for the modulation of tissue homeostasis [1]. Cancer cells frequently secrete several growth factors that stimulate myelopoiesis and recruit myeloid cells to TME. Therefore, the TMEs of various cancers are characterized by the high infiltration of monocytes, macrophages, granulocytes, and dendritic cells. Most myeloid cells within TMEs are present in an immature form; however, cancer-derived growth factors modify these myeloid cells into cells that support carcinogenesis by enhancing proliferation, migration, and metastasis and enabling cancer cell survival and immune evasion [2].

Acquired data from the oncology research is applicable in the novel clinical trend focused on 3PM strategies considered as the medicine of the future [3,4]. The presence of inflammatory or immune cells in specific tissues/organs was evaluated as a predictive marker in cancer therapy. Long-term oncology research indicates that chronic inflammation is associated with increased cancer risk and worsened disease prognosis [5]. Controlled inflammatory processes include specific immune responses - mainly linked with the maturation of antigen-presenting cells that promote tissue/organ healing. On the other hand, amplified, uncontrolled, or prolonged inflammation associated with immunosuppression is linked with many pathologies, including carcinogenesis [6]. It is widely accepted that up to 25% of neoplasms in humans are associated with chronic inflammation induced by viral and/or bacterial infections, physical and/or chemical stimuli, or metabolic pathologies [6–8]. Chronic inflammation influences tumor initiation and development by modulating critical signaling pathways associated with cellular transformation, proliferation, angiogenesis, survival, invasion, and metastasis [9]. The inflammation that initiates carcinogenesis exists long before cancer appears. In this regard, conditions such as chronic hepatitis, Helicobacter-induced gastritis, inflammatory bowel disease, and schistostoma-induced bladder inflammation increase the risk of several cancers, such as liver cancer, stomach cancer, colorectal cancer, and bladder cancer [8]. Numerous environmental factors must be considered, as they initiate and/or promote carcinogenesis by inducing organ-specific or systemic chronic inflammation. For instance, asbestos and tobacco smoke can induce lung and airway inflammation and thus initiate mesothelioma and lung carcinoma [10].

Similarly, low-grade systemic inflammation induced by hyperglycemia, hyperlipidemia, and obesity is a risk factor for several cancers, such as liver, pancreatic, colon, and breast cancers [11]. Abundant scientific evidence points out a significant role of systemic inflammation in cancer initiation, development, and progression. Obesity-, tobacco smoke-, and bacterial product-induced inflammation stimulates neutrophils and causes extracellular trap formation; these processes initiate cancer invasion [12]. The evaluation and modulation of the biological balance between controlled inflammation and uncontrolled chronic inflammation are essential for cancer prediction, prognostication, and prevention.

In the search for novel therapeutic agents that can precisely modulate cancer-associated inflammation, plant-derived natural products hold great promise due to their multi-targeting modes of action. Plant-derived compounds potently modulate inflammation. In the past several decades, many studies reported the anti-inflammatory effects of various phytochemicals *in vitro* and *in vivo*. In this regard, flavonoids could directly modulate the immune system [13]. Flavonoids exert anti-inflammatory actions through the regulation of immune cells, suppression of pro-inflammatory transcription factors, chemokines, cytokines, and COX-2, and inhibition of PI3K/AKT and IKK/JNK [14,15].

This article summarizes the most recent evidence regarding the role of flavonoids as suppressors of pro-inflammatory cell signaling pathways that can promote carcinogenesis. The evidence supports the preventive and therapeutic potential of flavonoids in cancer.

**The role of chronic inflammation in different stages of carcinogenesis**

*Cancer initiation*

Two main cellular events are necessary for cancer initiation: first, the accumulation of genetic and/or epigenetic changes in genes that control tumor suppression/oncogenic signaling pathways; and second, the formation of transformed and/or malignant clones. A qualitative change into a tumor follows; the pro-inflammatory microenvironment can significantly support this process. A prolonged pro-inflammatory microenvironment contributes to genomic instability and cancer initiation. Therefore, pathogenic infections lead to DNA damage and/or cellular metabolic deregulation and thus change genomic integrity. The TME could trigger the recruitment of pro-inflammatory cells, making the environment rich in chemokines, growth factors, cytokines, and DNA-damaging agents (ROS, RNS) that activate DNA damage response pathways. Long-term deregulation of these factors may cause abnormalities and related pathologies, including cancer [16]. Moreover, the adaptive nature of carcinogenesis is highlighted because cell clones with equal genetic changes have different propensities for development and survival. These processes are strongly affected by the TMEs of these clones [17].

Chronic inflammatory processes are associated with oxidative/nitrosative stress, and consequent electron “storms“ in cells/tissues induce progressive DNA damage. ROS/RNS originate from the immune system via specific NADPH oxidases that are crucial for pathogen clearance [18]. These DNA changes are linked with the deregulation of cellular homeostasis and result in genetic aberrations that act as initiation factors in chronic inflammation-induced pathogenesis, including carcinogenesis [19]. For example, chronic inflammation and ROS generation in colon epithelial cells can affect the Wnt/*β*-catenin and/or base excision repair pathways and thus increase the risk of polyp formation. The combination of DNA damage caused by oxidative stress and errors in DNA polymerase activity can support C>T transitions in various tissues, leading to a hypermutated cellular phenotype [20]. In another study using an inflammatory colorectal cancer model, the EMT factor ZEB1 promoted inflammation and progression towards inflammation-driven carcinoma by suppressing the DNA repair glycosylase MPG in epithelial cells [21]. Gobert et al. (2017) reported that polyamine- and NADPH-dependent ROS generation during *Helicobacter pylori* infections might lead to the initiation of inflammation and consequent carcinogenesis [22].

During chronic inflammation, pro-inflammatory mediators are continuously produced. Cytokines, growth factors, and acute-phase proteins, allpart of the TME modulate, the inflammatory responses and the crosstalk between signaling pathways involved in cancer initiation. Several papers describe the key role of the pro-inflammatory microenvironment in tumor initiation. Following infection or injury, SAA represents a critical acute phase protein secreted by hepatocytes during the APR. Autophagy is reported to be critical in tumors - both pro-and anti-carcinogenic. High levels of SAA in the TME contribute to carcinogenesis. SAA affects cell-signaling pathways, such as those associated with PI3K and MAPK, which modulate autophagy. Dysregulation of autophagy can initiate the transformation of normal cells to premalignant via metabolic stress, DNA damage, oxidative stress, and endoplasmic reticulum stress. In addition, autophagy can support the survival of transformed cells [23]. For instance, the type A receptor of IL-17 directly triggers pro-carcinogenic signaling in transformed enterocytes.

Moreover, the type A receptor of IL-17 stimulates ERK, p38 MAPK, and NF-κB signaling and induces the proliferation of transformed enterocytes that lack APC tumor suppressor functions [24]. Beneforti et al. (2020) hypothesized that infections and inflammation are the most important triggers of mutation accumulation in and the malignant transformation of ETV6-RUNX1 fusion gene (E/R^+^) pre-leukemic cells in childhood acute lymphoblastic leukemia. The authors showed that the pro-inflammatory cytokines IL-6/TNF-α/IL-1β coact with BM-MSC in promoting the emergence of E/R^+^ Ba/F3 cells (a murine IL-3 dependent pro-B cell line) and modulating their survival and proliferation. In addition, BM-MSCs attracted E/R^+^ Ba/F3 cells in a chemokine type 2 receptor-dependent manner; E/R^+^ human CD34^+^ IL7R^+^ progenitors (a supposed population of cells for acute lymphoblastic leukemia) were protected in the presence of BM-MSC and IL-6/TNF-α/IL-1β. The authors concluded that DNA damage accumulation depends on the extent of inflammation in both control and E/R^+^ Ba/F3 cells and might lead to the transformation of the apoptosis-resistant pre-leukemic clones [25]. Furthermore, Huang et al. (2020) found that IL-1β, secreted by macrophages stimulated by multi-walled carbon nanotubes, increased the release of pro-inflammatory cytokines such as TNF-α, IL-8, and IL-6 from mesothelial cells. The authors concluded that inflammation-induced NF-κB (p65)/IL-6/STAT3 signaling plays a crucial role in the malignant transformation of pleural mesothelial cells [26].

Different cytokines and chemokines may modulate the conversion of normal fibroblasts into CAFs. CAFs synthesize pro-inflammatory cytokines such as IL-6 and LIF, which in turn modulate their epigenetic status. This activity promotes the pro-tumorigenic function of CAFs by enhancing actomyosin contractility and ECM remodelling [27]. IL-1β is another pro-inflammatory cytokine that initiates carcinogenesis and promotes immunosuppression. These functions of IL-1β are mainly observed in the early stages of carcinogenesis; moreover, its expression significantly impacts the process of malignant transformation [28].

*Cancer promotion*

Like cancer initiation, a pro-inflammatory TME and consequent signaling can support numerous growth factors in promoting carcinogenesis. Cancer promotion can be regulated by several pathways associated with inflammation, such as those involving NF-κB, STAT3, mTOR, and MAPKs, which are triggered by pro-inflammatory cytokines like IL-6, TNF-α, and IL-1β [29]. Ju et al. (2020) revealed that infiltrated macrophages released TNF-ɑ and IL-6, which induced the expression of PD-L1 in cancer cells. Consequently, TNF-ɑ and IL-6 triggered the activation of the NF-κB and STAT3 signaling pathways to modulate PD-L1 expression. The authors concluded that macrophage-induced increases in PD-L1 expression could allow cancer cells to escape from cytotoxic T cell surveillance and proliferate [30]. PIM2 is an oncomarker highly expressed in HCC which correlates with poor prognosis. Functional studies demonstrated that PIM2 is an enhancer of cell proliferation, cell motility, angiogenesis, and chemoresistance in cancer. In this regard, HCC cells treated with TNF-α showed increased PIM2 expression; this consequently increased the expression of TNF-α. The same study revealed that PIM2 promotes HCC carcinogenesis by activating the NF-κB signaling pathway through PIM2 receptor phosphorylation [31].

S100A7/RAGE signaling in adjacent endothelial cells may behave as a crucial angiocrine effector. Muoio et al. (2021) found that IGF-1/IGF-1R signaling increases STAT3 activation in breast cancer cells. Consequently, activating the S100A7/RAGE pathway via STAT3 signaling sustains angiogenesis and thus stimulates cancer promotion in breast cells [32]. The upregulation of cancer-promoting inflammatory cytokines (host and tumor-secreted), such as NF-κB, COX2, IL-1, IL-6, TNF-α, and IFN-γ, is strongly associated with STAT3 and AKT signaling activation in oral squamous cell carcinoma *in vitro*. The suppression of these cytokines may decrease tissue inflammation and thus decrease the proliferation of oral squamous cell carcinoma cells [33]. TAMs play a substantial role in modulating the interactions between cancer cells and the immune system. In this regard, the TAM microenvironment promoted the growth of triple-negative breast cancer cells in a study by Deng et al. (2021). The authors described that TAMs promoted the growth of MDA-MB-231 and MDA-MB-468 cells by upregulating the IL-10/STAT3/PD-L1 immunosuppressive signaling pathway [34]. Zhang et al. (2017) revealed that TAMs might trigger PD-L1 expression by producing IFN-γ via the JAK/STAT3 and PI3K/AKT signaling cascades in A549 cells [35].

Recent data revealed that MAPK upregulation is crucial in modulating inflammation-associated cancer development. MAPKs include p38 MAPK, JNK, and ERK. These enzymes are serine-threonine protein kinases that affect essential cellular activities, such as proliferation, differentiation, apoptosis, survival, inflammation, and innate immunity. The described enzymes are upregulated by various types of cellular stress and pro-inflammatory cytokines such as TNF-α and IL-1β [36]. In addition, increased p38 MAPK signaling is linked with increased proliferation and decreased apoptosis of colon and liver cancer cells. Deregulation of the mTOR signaling pathway, including the loss of *PTEN* function, amplification/mutation of PI3K, and overexpression of S6K1, 4EBP1, eIF4E, and AKT, was described in multiple cancer types but mainly in melanoma [37]. In a preclinical study, lung cancer-induced osteoclastogenesis *in vitro* was associated with the upregulation of IL-6 and TNF-α and consequent activation of the AMPK/mTOR signaling pathway [38].

*Cancer progression*

Cancer metastasis is a highly inefficient process. Most cancer cells released from the primary tumor site die before creating a distant metastatic site. To further improve the ratio of dead cancer cells with invasive phenotypes, it is possible to modulate specific “key steps” within the complex process of tumor progression that may decrease the “success” of metastatic invasion and improve patient survival. Metastasis begins with the invasion of tumor cells from the epithelium into the surrounding tissues and the concurrent EMT. The post-EMT phenotype of cells (often only partial) allows them to cross the basal epithelial membrane and reach lymphatic and/or blood capillaries [8,39]. A recent study described a novel lymphatic pattern in the hypoxic TME, wherein TAMs encapsulate lymphatic vessels to form an interconnected network. These aggregates are advantageous for and actively involved in early lymph node metastasis [40].

Moreover, research data revealed the importance of the pro-inflammatory TME within the ECM in modulating tumor-associated processes such as invasion, metastasis, angiogenesis, immune cell modulation, and therapeutic resistance [41]. Calon et al. (2015) reported that all colorectal cancer subtypes with poor prognosis are characterized by stromal cancer cells with a TGF-β-triggered transcription program associated with a pro-inflammatory TME. The application of TGF-β signaling inhibitors suppressed the cross-talk between the TME and cells and thus suppressed cancer progression in patient-derived tumor organoids and xenografts [42].

CSCs are more effective in metastasis than other (bulk) tumor cells. CSCs are essential for cancer metastasis and therapeutic resistance. Numerous stimuli such as chronic inflammatory signaling, including the activation of the STAT3 and NF-κB transcription factors, may drive the stemness of CSCs in cancer tissue and enlarge their proportion within the cell population, thereby increasing the potential of metastasis. Importantly, CSCs are functionally and transcriptionally more related to mesenchymal cells than bulk tumor or normal epithelial cells [43]. Cytokines and chemokines, both their receptors and signaling pathways, are essential factors in the complex interplay and crosstalk between different cell types and secreted factors in the events that lead to cancer metastasis. Numerous cytokines and chemokines, such as TNF, IL-6, IL-8, CXCL12, TGF-β, CXCL8, VEGF, RANKL, CCL2, CX3CL1, IL-1, IL-7, CXCL1, and CXCL16, contribute to the regulation of cancer metastasis [44,45].

Moreover, comprehensive data support the role of a pro-inflammatory TME in the promotion of CSCs. Cytokines and chemokines may benefit cancer cells and/or CSCs in processes such as cell proliferation, survival, and migration [46]. In this regard, there exists a close linkage between CSCs and inflammatory components, i.e., inflammatory cells such as TAMs and MDSCs and inflammatory cytokines (TNF, IL‑6, IL‑17, IFNs) [47].

Metastatic spread typically occurs through blood and lymphatic vessels. Therefore, intravasation and extravasation are essential processes in cancer metastasis. These processes are modulated by specific adhesion molecules and integrins facilitating cell-cell interactions/adhesion and cell movement. In this regard, inflammatory cytokines are important inducers of integrins, selectins, and adhesion molecules, such as VCAM-1 and ICAM-1. In addition, TAMs produce pro-angiogenic growth factors and MMPs. Therefore, they support vasculogenesis and consequently the supply of oxygen and nutrients to solid tumors [48]. A preclinical study by Horiguchi et al. (2020) demonstrated that local inflammation, characterized by elevated IL-6, TNF-α, and HGF in the bronchoalveolar lavage fluid, increased the metastatic activity of NL-17 cells in the lung. A pro-inflammatory TME significantly upregulated the expression of α2 integrin, VCAM-1, and ICAM-1. An anti-ICAM-1 antibody suppressed the invasive activity of NL-17 cells; therefore, adhesion molecules have a potential role in lung metastasis enhanced by local inflammation [49].

Comprehensive research has described specific cellular and molecular events that may cause tumor cells to escape immune surveillance, including those involving tumor-induced myeloid cell-mediated immunosuppression [50]. Multiple studies indicate that tumor-infiltrating myeloid cells accelerate cancer growth and support angiogenesis, metastasis, and therapeutic resistance after their conversion into potent immunosuppressive cells. Host and tumor cells in the TME secrete pro-inflammatory molecules that stimulate MDSCs and trigger their accumulation and suppressive activities [51]. Several mechanisms driven by MDSCs suppress T cells and activate immunosuppressive cell populations. Such inflammatory modulation in the TME causes the immune system to tolerate cancer cells and enhance their growth.

In conclusion, the interruption of the interplay between pro-inflammatory cytokines and pro-inflammatory/pro-carcinogenic cell signaling pathways affects all stages of carcinogenesis and might therefore represents a promising strategy in oncology research and practice.

#### **Anti-inflammatory activities of flavonoids: implications in carcinogenesis**

Flavonoids constitute a group of natural substances with different phenolic structures. They are found in fruits, vegetables, grains, flowers, tea, bark, roots, and stems. These natural molecules have well-described beneficial health effects in humans. Due to their anti-oxidative, anti-inflammatory, anti-mutagenic, anti-carcinogenic, and cellular enzyme modulating activities, flavonoids have numerous medicinal, pharmaceutical, and cosmetic applications. A substantial proportion of non-infectious diseases develops or is worsened by the chronic inflammatory process. Flavonoids are reported to combat most inflammatory processes underlying chronic conditions such as carcinogenesis [52–61].

*Cancer initiation*

Pro-inflammatory cytokine production and consequent intracellular ROS and RNS accumulation lead to DNA damage and initiate carcinogenesis. Flavonoids, as antioxidants, inhibit regulatory enzymes and transcription factors important for controlling inflammatory mediators. Moreover, they modulate cellular oxidative stress by interacting with DNA and enhancing genomic stability [62]. Flavonoids can inhibit DNA adduct formation, enhance DNA repair by interfering with genotype damage caused by the up-regulation of Phase II enzymes, and modify relevant signaling pathways [63]. It is well documented that specific flavonoids can reduce cytokine production; therefore, they may have preventive and therapeutic potential in inflammation-related diseases (such as cancer) [64]. TNF-α triggers the release of chemotactic proteins, e.g. MCP-1/CCL2. These regulatory molecules direct the infiltration and migration of TAMs, MDSCs, Tregs, TANs, Th17s, MAMs, and CAFs. Specific flavonoids can attenuate the TNF-α-induced release of MCP-1/CCL2 and various recruiting cytokines from cancer cells [65,66]. In addition, by targeting TGF-β2, flavonoids suppress the cancer cell-mediated differentiation of naive fibroblasts into cancer-associated fibroblasts [67].

*Cancer promotion*

The inhibitory effect of flavonoids on cell proliferation and thus cancer promotion is associated with decreased phosphorylation of STAT-induced signaling pathway components and related transcriptional activators. Hou et al. (2019) revealed that flavonoid treatment inhibits recurring colitis and colitis-associated tumorigenesis; these flavonoids downregulate IL-1β and TNF-α and consequently inhibit inflammation-induced colorectal cancer *in vitro* [68]. NF-κB signaling plays a crucial role in inflammation and cancer growth. Flavonoids negatively regulate the NF-κB signaling pathway by suppressing kinase phosphorylation, inhibiting NF-κB translocation into the nucleus, and blocking interactions between DNA and NF-κB. Through these mechanisms, flavonoids inhibit inflammatory cascades associated with decreased cell proliferation, apoptotic induction, and the suppression of vasculogenesis [69]. In addition, flavonoids are promising anti-cancer agents that target the inflammatory PI3K/AKT/mTOR/p70S6K and ERK/MAPK signaling pathways [70,71].

*Cancer progression*

Flavonoids exert chemopreventive effects by protecting against cancer progression, inhibiting CSC formation, and alleviating lung metastasis in a preclinical model [72]. The reduction in cancer metastasis was associated with regulating the PI3K/AKT, MAPK/ERK, and STAT3 pathways - central CSC-associated inflammatory signaling cascades. In another study, natural mixtures of flavonoids significantly downregulated the pro-inflammatory cytokines COX-2, iNOS, and TNF-α and the pro-angiogenic factors VEGF and eNOS, and induced apoptosis by increasing the Bax/Bcl-2 ratio *in vitro* [73]. In this regard, flavonoids are anti-inflammatory agents that downregulate crucial modulators of advanced stages of cancer such as IL-1β, IL-6, IL-10, TNF-α, NF-κβ, NOS2, PTGS2, PTGER2, ACAN, COL2A1, MMP1, MMP13, ADAMTS4, ADAMTS5, and TIMP1. These changes correlated with the reduction of serum levels of PGE2, CTX-II, TNF-α, MMP1, MMP13, PIINP, OPG, RANKL, OC, and BALP in a rat model [74]. Another study found that flavonoids exert their anti-metastatic activities by reducing CXCR4 expression and can therefore support the blockade of cancer promotion [75]. Furthermore, flavonoids target the integrin modulated ILK/YAP pathway and block the EMT and metastasis [76]. Moreover, flavonoids block angiogenesis and metastasis by suppressing VEGF-induced oxidative stress and NF-κB signaling and downregulating adhesion molecules such as VCAM-1, ICAM-1, and E-selectin; these activities decrease the formation of new blood capillaries in cancer tissue [77]. Finally, MDSC activation is involved in chronic inflammation-related immunosuppression and CD4^+^/CD8^+^ T-cell activation through the ERK/IL-6/STAT3 and Arg-1/iNOS/Nox2/NF-κB/STAT3 signaling pathways. In this regard, flavonoids attenuate MDSC-mediated immunosuppression that is crucial for cancer growth and metastasis [78,79].

**Flavonoids prevent tumor initiation by modulating inflammatory processes**

The concept of cancer chemoprevention by flavonoids was investigated in numerous *in vivo* and *in vitro* studies [80–84]. These natural phenolic compounds can modulate different steps of carcinogenesis [85]. As mentioned above, chronic inflammation accelerates genetic/epigenetic aberrations that lead to cancer initiation, promotion, and progression [86]. Prolonged chronic inflammation associated with the overexpression of inflammatory mediators in the cell microenvironment is a critical step that promotes cancer initiation [87]. The anti-inflammatory properties of flavonoids could prevent, suppress, and reverse cancer initiation [88,89]. In the following section of the manuscript, we provide an overview of recent studies that analyze the role of flavonoids in tumor initiation through the modulation of inflammatory responses *in* *vitro* and *in vivo*.

*Preclinical research*

Baicalin, a flavonoid isolated from the roots of *Scutellaria baicalensis*, exerts different oncostatic effects [90,91]. ROS generation due to constant UV-A exposure causes the formation of inflammatory products and the accumulation of DNA mutations [92]. The protective role of baicalin was investigated in an animal study that analyzed the association between UV-A irradiation, ROS production, and inflammation. The acquired data revealed that baicalin protects against UV-A-induced inflammation and oxidative damage by increasing IL-12 and IL-23. These effects are likely mediated by suppressing the TLR4 pathway, which has a significant role in inflammation [93]. Additionally, baicalin can be transformed into baicalein by intestinal microbiota. The anti-inflammatory and anti-cancer effects of baicalein were evaluated in an animal model of colorectal cancer. The authors used a gut-specific C57BL/6J Apc^Min/+^/J mouse model (APC gene mutants) to evaluate parameters such as life span, tumor multiplicity, and organ index. Moreover, the expression of inflammatory cytokines was measured. Baicalein administration (30 mg/kg/day) decreased the number of tumors in the small intestine and colon (after 10 weeks of supplementation by baicalein) compared to controls. In addition, baicalein administration suppressed the expression of pro-inflammatory cytokines (IL-1β, IL-2, IL-6, IL-10, GM-CSF, and G-CSF) [94]. Ulcerative colitis is classified as a chronic idiopathic IBD [95]. Patients with IBDs are at increased risk for the development of extra-intestinal malignancies [96]. Rutin is a naturally occurring flavonoid with profound effects on different cellular processes associated with a pathological phenotype [97]. As demonstrated in a DSS-induced experimental colitis model *in vivo,* rutin modulated the expression of pro-inflammatory genes. Rutin administration significantly suppressed the protein level of IL-1β and the expression of *IL-1β* and *IL-6* mRNA in the colonic mucosa of mice. Moreover, rutin attenuated DSS-induced colitis symptoms, including weight loss and colorectal shortening, and improved the histological score of colitis in the tested animals [98]. Another flavonoid, myricetin, downregulates inflammatory factors including TNF-α, IL-6, IL-1β, NF-κB, p-NF-κB, PCNA, COX-2, and cyclin D1 in AOM/DSS induced colitis in mice. Myricetin significantly reduced the number of colorectal tumors and decreased the size of polyps in the colon [99]. Furthermore, naringin prevented AOM/DSS-induced colitis and carcinogenesis in mice by suppressing MDSCs. A deeper analysis identified the downregulation of GM-CSF/M-CSF, IL-6, and TNF-α and the inhibition of the NF-κB/IL-6/STAT3 pathway as further contributors to colitis-associated cancer [100].

Chronic arsenic exposure is associated with inflammation that could initiate carcinogenesis [101]. EGCG, a natural compound isolated from green tea, is a potent anti-inflammatory molecule [102]. EGCG exhibited an anti-inflammatory effect against arsenic (NaAsO2)-induced inflammation in mice. The acquired data revealed that EGCG administration downregulated the pro-inflammatory cytokines IL-1β, IL-6, and TNF-α. Additionally, EGCG attenuated oxidative stress by upregulating markers such as CAT, SOD, and GSH and decreasing MDA content in mice [103]. Furthermore, the administration of GTPs influenced UV-B-induced tumor development in mice. Likewise, UV-B is strongly associated with chronic inflammation and, as mentioned above, there is a strong relationship between UV radiation-induced chronic inflammation and skin carcinogenesis. GTPs suppressed the pro-inflammatory markers COX-2, PGE2, PCNA, and cyclin D1. Furthermore, levels of the pro-inflammatory cytokines TNF-α, IL-6, and IL-1β were reduced after GTP administration [104]. Chronic gastritis and peptic ulceration - chronic inflammatory disorders caused by *Helicobacter pylori* infection – are strongly associated with an increased risk of cancer development [105,106]. The isoflavonoid genistein exerted anti-inflammatory effects in rats infected by *Helicobacter pylori*. Its anti-inflammatory properties were mediated by suppressing pro-inflammatory mediators such as TNF-α and CINC-1, NF-κB, and bacterial infection-induced gastric epithelial cell apoptosis [107]. Hesperidin, a flavone extracted from citrus fruits, has many biological effects [108]. Its impact on ulcerative colitis was documented in a study that evaluated the anti-inflammatory effect of hesperidin methyl chalcone in an animal model of acetic acid-induced colitis. As demonstrated above, chronic inflammation promotes colitis-associated cancer; thus, the attenuation of pro-inflammatory cytokines is a promising way to suppress cancer initiation [109]. Hesperidin significantly catalyzed colon antioxidant processes and suppressed pro-inflammatory cytokines (IL-33, IL-1β, IL-6, and TNF-α) *in vivo* [110]*.* In addition, quercetin exhibited anti-inflammatory effects in another model of DSS-induced experimental colitis *in vivo*. Comalada et al. (2005) evaluated the effect of quercitrin, a glycoside of quercetin that is cleaved by gut microbiota to generate quercetin. Quercetin significantly inhibited cytokine (IL-1β and TNF-α) production and induced the expression of iNOS by downregulating the NF-κB pathway [111]. Moreover, the flavonoid luteolin possesses various beneficial properties for human health [112–114]. Luteolin significantly reduced malignant transformation induced by hexavalent chromium [Cr(VI)] in human bronchial epithelial cells (BEAS-2B). Recurrent exposure to [Cr(VI)] is connected to persistent inflammation and subsequent carcinogenesis [115]. Noteworthy, luteolin is known to downregulate AP-1, HIF-1α, COX-2, and iNOS. In addition, luteolin prevented the transformation of BEAS-2B into malignant phenotypes. The obtained data revealed decreases in IL-1β, IL-6, IL-8, and TNF-α levels. Western blotting uncovered the inhibition of products associated with inflammation, including MAPK, NF-κB, COX-2, STAT-3, and iNOS in chronic [Cr(VI)] exposed cells. In addition, luteolin reduced tumor incidence in mice injected with [Cr(VI)]-exposed BEAS-2B cells [116]*.* Furthermore, cigarette smoke modulates inflammation and induces chronic inflammation. Therefore, Pace et al. (2019) evaluated the impact of CSE on normal (16HBE) and cancerous (A549) epithelial cells. The study authors applied the naturally occurring flavone apigenin, which reduced miR-21 and IL-8 gene expression in both cell lines [117].

As we described in this chapter, flavonoids possess pleiotropic abilities in preventing cancer initiation through the suppression of chronic inflammation. The health benefits of flavonoids targeting inflammatory pathways identified in experimental studies could be translated into the clinical area. The investigation of novel therapeutic approaches to reverse and prevent cancer initiation through the modulation of chronic inflammation falls within the concept of 3PM. Table 1 summarizes the effects of flavonoids on molecular cascades associated with inflammation and subsequent carcinogenesis.

**Table 1.** Flavonoids targeting inflammatory pathways associated with cancer initiation

| **Flavonoid** | **Study design** | **Mechanisms** | **Dosage of the Tested Flavonoid** | **References** |
| --- | --- | --- | --- | --- |
| Baicalin | Female C3H/HeN mice | Baicalin treatment inhibited ROS production via the downregulation of p47p^hox^ , a key component of NADPH oxidase. Furthermore, baicalin inhibited inflammatory cascades through TLR4 suppression | 4 mg baicalin per mouse | [93] |
| Baicalein | C57BL/6J ApcMin/+/J mouse model | Baicalein reduced the number of tumors in the small intestine (P<0.01) and colon (P<0.05) in the baicalein-treated group compared to the non-treated group. Additionally, ELISA analysis of small intestine and colon tissue revealed the downregulation of pro-inflammatory cytokines, including IL-1β, IL-2, IL-6, IL-10, GM-CSF, and G-CSF | 30 mg/kg/day | [94] |
| Rutin | Female ICR mice | ELISA analyses revealed the downregulation of IL-1β in the colonic mucosa. Moreover, the mRNA levels of *IL-1β* and *IL-6* were decreased after rutin administration. Symptoms of DSS-induced colitis were attenuated by rutin | 6 mg/day, 0.6 mg/day and 60 mg/day | [98] |
| Myricetin | Male BALB/c mice | Myricetin administration reduced tumorigenesis and inflammation *in vivo*. Western blot and qPCR analyses revealed decreases in the levels of pro-inflammatory markers (TNF-α, IL-6, IL-1β, NF-κB, p-NF-κB, PCNA, COX-2, and cyclin D1) in mice | 40 mg/kg and 100 mg/kg | [99] |
| Naringin | Male C57BL/6 mice | Oral administration of naringin prevented colitis and carcinogenesis induced by AOM/DSS through the reduction of GM-CSF/M-CSF, IL-6, and TNF and inhibition of the NF-κB/IL-6/STAT3 pathway | 50 and 100 mg/kg | [100] |
| EGCG | Male BALB/c mice | EGCG decreased oxidative stress (increased SOD, CAT, and GSH activity, and decreased MDA and nitric oxide). EGCG decreased the levels of pro-inflammatory cytokines, including IL-1β, IL-6, and TNF-α | 10 mg/kg | [103] |
| GTPs | Female C3H/HeN mice, IL-12p40KO mice on a C3H/HeN background | GTPs inhibited UV-B-induced skin carcinogenesis by downregulating pro-inflammatory markers (COX-2, PGE2, PCNA, TNF-α, IL-6 and IL-1β) in wild type mice. GTP administration in their counterparts, IL-12p40 knockout mice, was less effective than in WT mice. | water containing GTPs (0.2%, w/v) | [104] |
| Genistein | Sprague-Dawley rats | Genistein inhibited *Helicobacter p*.-induced gastropathy by suppressing pro-inflammatory cytokine (TNF-α and CINC-1) production, NF-κB activity, and gastric cell apoptosis. | 16 mg/kg | [107] |
| Hesperidin | Male Swiss and LysM-eGFP mice | Hesperidin demonstrated anti-inflammatory effects by inhibiting ROS generation and IL-33, IL-1β, IL-6, and TNF-α cytokine production through the inhibition of NF-κB. In addition, hesperidin attenuated colitis symptoms, including bowel edema, colon shortening, and macroscopic lesions. | 10, 30, or 100 mg/kg in saline | [110] |
| Quercetin | Female Wistar rats | Administration with Quecetrin (1mg/kg/day) suppressed IL-1β, TNF-α, and iNOS expression through the inhibition of the NF-κB pathway in a rat model of dextran sulfate sodium-induced colitis. | 1 mg/kg/day | [111] |
| Luteolin | BEAS-2B cells; [Cr(VI)]-induced BEAS-2B cells injected into mice | Luteolin treatment suppressed the promoter activity of AP-1, HIF-1α, COX-2, and iNOS and the production of IL-1β, IL-6, IL-8, and TNF-α in BEAS-2B cells. Western blot analysis revealed decreases in MAPK, NF-κB, COX-2, STAT-3, iNOS and TNF-α protein levels *in vitro*. Reduction of tumor frequency in mice injected with [Cr(VI)]-exposed BEAS-2B cells | 1 and 2 μM | [116] |
| Apigenin | 16HBE and A549 cells | Apigenin reduced miR-21 and IL-8 mRNA expression in normal and cancerous cells exposed to CSE | 20 μM | [117] |

**Flavonoids as anti-inflammatory agents against tumor promotion in carcinogenesis**

*Isolated flavonoids in preclinical studies*

Chronic inflammation predisposes to cancer development and subsequently promotes all stages of tumorigenesis [8]. The anti-inflammatory and other anti-cancer effects of flavonoids were evaluated in cancer research since the last century. GTPs, silymarin, and apigenin inhibited skin tumor promotion by decreasing skin papilloma formation in DMBA-induced and TPA-promoted SENCAR mice [118–120].

As discussed above, various signaling pathways are associated with chronic inflammation, including the MAPK, AKT, mTOR, STAT3, and/or NF-κB pathways, and can potentially lead to tumor promotion. Therefore, in the 21^st^ century, studies emphasize the precise molecular mechanisms and signaling pathways leveraged by flavonoids against inflammation in various stages of carcinogenesis.

Quercetin inhibited the PI3K/AKT/mTOR, Wnt/β-catenin, and STAT3 signaling pathways in BC3, BCBL1, and BC1 primary effusion lymphoma cells. Further, quercetin inhibited tumor promotion by downregulating the pro-inflammatory cytokines IL-6 and IL-10, pro-survival molecules downstream of PI3K/AKT/mTOR, Wnt/β-catenin, and STAT3 such as c-FLIP_L_, and molecules linked to cell proliferation, including cyclin D1 and cMyc [121].

Moreover, icaritin from a Chinese herbal medicine (*Epimedium* species) inhibited proliferation and tumor growth and induced apoptosis of K562 and primary chronic myeloid leukemia cells. A mouse model also revealed that icaritin modulates signaling pathways associated with chronic inflammation such as MAPK/ERK/JNK and JAK2/STAT3/AKT by upregulating p-JNK and p-C-JUN and downregulating p-ERK, p-P38, JAK-2, p-JNK, p-STAT3, and p-AKT in a dose- and time-dependent manner [122].

Furthermore, fisetin suppressed the expression of inflammatory mediators and ICAM-1 in IL-1β-promoted inflammation in A549 lung adenocarcinoma cells. Further, administrating fisetin downregulates COX-2, PGE2, IL-8, CCL5, MCP-1, TNF-α, and IL-6. Moreover, fisetin exerted anti-inflammatory effects through the suppression of the NF-κB and ERK1/2 signaling pathways in IL-1β-stimulated A549 cells, suggesting to prevent tumor promotion [123]. In another study, fisetin induced apoptosis by increasing caspase-3 expression and regulating the inflammatory PI3K/AKT/NF-κB signaling pathway in TU212 head and neck squamous cell carcinoma cells. Further, the suppression of TU212 cell proliferation and consequent changes in the tumor volume and weight of nude mice after fisetin treatment were connected to decreased Ki67 levels and the inactivation of ERK1/2 and PI3K/AKT-regulated mTOR [124]. Additionally, the combination of fisetin with carnosic acid enhanced their anti-inflammatory effects against tumor promotion of HCC827 and H358 human lung cancer cell lines and their murine xenografts. Co-treatment of fisetin and carnosic acid induced apoptosis by upregulating caspase-3, Bax and Bad, and death receptor of TRAIL, and downregulating the anti-apoptotic Bcl-2 and Bcl-xl proteins [125].

Interestingly, luteolin inhibited U251 and LN229 glioma cell proliferation through the induction of apoptosis. The apoptosis of U251 and LN229 cells was mediated by the anti-inflammatory effects of luteolin and associated activation of MAPKs (JNK, ERK, and p38) and death receptors (FADD) that regulated apoptotic proteins (caspase-8, caspase-3, and PARP). Further, luteolin promoted cell autophagy by upregulating LC3B II/I and downregulating p62 [126]. Moreover, luteolin reduced the expression of the STAT3 signaling pathway target gene Mcl-1, Survivin, and Bcl-xl - downregulation of which reduced inflammation in SGC7901, SGC7901/DDP, HGC27, MGC803, BGC803, and BGC823 gastric cancer cell lines. Inhibition of the STAT3 pathway after luteolin administration was mediated by the disruption of HSP90-STAT3 binding, which promoted its interaction with SHP-1. The anti-cancer efficacy of luteolin was confirmed in SGC7901, SGC7901/DDP, and HGC27 murine xenograft models [127]. Furthermore, luteolin and its derivative apigenin had a synergistic effect against H358 murine xenografts and Lewis lung carcinoma *in vivo*. Luteolin and apigenin inhibited lung cancer cell growth, induced apoptosis, and reduced IFN-γ-induced PD-L1 expression at inflammatory sites by suppressing STAT3 phosphorylation [128]. Due to its anti-inflammatory and antioxidant properties, apigenin also inhibited tumor promotion of by HepG2 HCC through inhibited cell proliferation and induced apoptosis and autophagy via PI3K/AKT/mTOR pathway inhibition [129]. Moreover, apigenin inhibited the phosphorylation of the signaling molecules Lyn, Syk, phospholipase Cγ1, ERK, and JNK and the expression of the cytokines TNF-α, IL-4, IL-5, IL-6, IL-13, and COX-2 – all of which induce inflammation and promote carcinogenesis – in RBL-2H3 rat leukemia cells [130]. Another study indicated that apigenin inhibited IL-6 expression and cell proliferation and promoted apoptosis by activating PARP and caspase-8 in Eca-109 and Kyse-30 human esophageal cancer cells [131].

*Plant extracts rich in flavonoids*

Beyond isolated flavonoids, various plant extracts and flavonoids in whole plant foods exert beneficial effects against inflammation and associated tumor promotion. Co-administration of bilberry extracts and enzymatically modified isoquercitrin suppressed the promotion of HCC in PBO-promoted rats. This combination inhibited proliferation by reducing Ki67 and microsomal ROS levels. Bilberry extracts and isoquercitrin decreased p-PTEN, p-AKT, and Smad4 signaling – the downregulation of which was also connected with the inhibition of inflammation in PBO-promoted cases [132,133]. Furthermore, Id1 is overexpressed in non-small cell lung carcinoma and exerts pro-inflammatory and tumor-promoting effects. *Scutellaria* flavonoids, especially the three prominent representatives (baicalin, baicalein, and wogonin), inhibited Id1 through the activation of Rap1-GTP binding and the dephosphorylation of AKT and Src in A549 cells, H1299 non-small cell lung carcinoma cells, and murine A549 xenografts [134]. In another study, baicalein and baicalin inhibited tumor promotion by downregulating PD-L1 and pro-inflammatory cytokine IFN-γ. These results were associated with STAT3 suppression in the SMMC-7721 and HepG2 human liver cancer cell lines [135]. Moreover, flavonoid-rich ethanol extracts of whole dried sugarcane reduced NF-κB phosphorylation and IL-8 secretion in SW480 colon cancer cells [136].

*Clinical studies*

Only limited clinical studies are based on the consistent molecular mechanisms of flavonoids against inflammation in different tumor stages. A botanical drug called APG-157 containing multiple polyphenols suppressed tumor cells due to its antioxidant and anti-inflammatory properties, as demonstrated by reduced IL-1β, IL-6, and IL-8 concentrations in the salivary supernatant fluid of oral cancer patients [137]. Moreover, fisetin improved the inflammatory status of colorectal cancer patients by reducing IL-8 and hs-CRP levels [138]. Furthermore, consumption of green tea reduced NF-κB-associated inflammation in the radical prostatectomy tissue of men compared with black tea and water controls, suggesting that GTPs play a beneficial role in prostate cancer prevention and treatment [139]. Despite the known anti-inflammatory effects of soy in men with prostate cancer [140], in women with early-stage breast cancer with the potential of tumor promotion and progression, soy supplements rich in genistein and daidzein led to the overexpression of cell cycle transcripts, including those that promote cell proliferation such as FGFR2, E2F5, BUB1, CCNB2, MYBL2, CDK1, and CDC20 [141].

Flavonoids can suppress tumor promotion of various cancer types through diverse anti-inflammatory mechanisms. Therefore, the evaluation of molecular and cellular mechanisms of tumor-promoting inflammation is essential for developing preventive and anti-cancer therapies in the preclinical and clinical spheres. Discussed anti-inflammatory effects of flavonoids against tumor promotion are summarized in Table 2. Moreover, Figure 1 depicts the efficacy of flavonoids in tumor initiation and promotion, and the affected signaling molecules and pathways.

**Table 2. Suppression of tumor promotion by anti-inflammatory effects** of flavonoids

| **Flavonoid** | **Cancer** | **Study design** | **Anti-inflammatory effects and/or mechanisms of tumor suppression** | **Ref** |
| --- | --- | --- | --- | --- |
| **Preclinical studies** | | | | |
| Green tea polyphenols | skin tumor | six-week-old female SENCAR mice | ↓ stage I and stage II skin tumor promotion, ↓ skin papilloma formation, ↓ tumor multiplicity, ↓ tumor incidence, ↓ tumor growth | [118] |
| Silymarin |  |  | ↓ stage I and stage II skin tumor promotion, ↓ skin papilloma formation, ↓ tumor multiplicity, ↓ tumor incidence, ↓ tumor growth | [119] |
| Apigenin |  |  | ↓ skin papilloma formation, ↓ incidence of carcinomas/papillomas | [120] |
| Quercetin | primary effusion lymphoma | BC3, BCBL1, and BC1 primary effusion lymphoma cells | ↓ PI3K/AKT/mTOR, ↓ Wnt/β-catenin, ↓ STAT3, ↓ IL-6, ↓ IL-10, ↓ c-FLIP_L_, ↓ cyclin D1, ↓ cMyc | [121] |
| Icaritin | chronic myeloid leukemia | K562 and primary chronic myeloid leukemia cells; 6-8 week old female NOD-SCID nude mice | ↓ proliferation, ↓ tumor growth, ↑ apoptosis, regulation of MAPK/ERK/JNK, regulation of JAK2/STAT3/AKT, ↑ p-JNK, ↑ p-C-JUN, ↓ p-ERK, ↓ p-P38, ↓ JAK-2, ↓ p-JNK, ↓ p-STAT3, ↓ p-AKT | [122] |
| Fisetin | lung cancer | IL-1β-promoted inflammatory responses of A549 lung adenocarcinoma cells | ↓ ICAM-1, ↓ COX-2, ↓ PGE2, ↓ IL-8, ↓ CCL5, ↓ monocyte chemotactic protein 1, ↓ TNF- α, ↓ IL-6, ↓ NF-κB, ↓ ERK1/2 | [123] |
|  | laryngeal carcinoma | TU212 head and neck squamous cell carcinoma cells; 6–8-week-old SPF male BALB/c nude mice | ↑ apoptosis, ↑ caspase-3, regulation of PI3K/AKT/NF-κB signaling, ↓ proliferation, ↓ tumor volume and weight, ↓ KI67, ↓ ERK1/2, ↓ PI3K/AKT-regulated mTOR | [124] |
| Fisetin + carnosic acid | lung cancer | HCC827 and H358 human lung cancer cell lines; eight-week-old athymic nude mice injected with HCC827 and H358 cells | ↑ anti-inflammatory effects, ↑ apoptosis, ↑ caspase-3, ↑ Bax, ↑ Bad, ↓ Bcl-2, ↓ Bcl-xl, ↑ death receptor of TRAIL | [125] |
| Luteolin | glioma | U251 and LN229 glioma cells | ↓ proliferation, ↑ apoptosis, ↑ MAPK, ↑ JNK, ↑ ERK, ↑ p38, ↑ FADD, ↑ caspase-8, ↑ caspase-3, ↑ PARP, ↑ autophagy, ↑ LC3B II, ↑ LC3B I, ↓ p62 | [126] |
|  | gastric cancer | SGC7901, SGC7901/DDP, HGC27, MGC803, BGC803, and BGC823 gastric cancer cell lines; six-week-male nude Balb/c SGC7901, SGC7901/DDP and HGC27 murine xenografts | ↓ STAT3, ↓ Mcl-1, ↓ Survivin, ↓ Bcl-xl, disruption of the binding of HSP-90 to STAT3, ↓ tumor growth | [127] |
| Luteolin + apigenin | lung cancer | H358 murine xenografts and Lewis lung carcinoma *in vivo* model | ↓ lung cancer cell growth, ↑ apoptosis, ↓ IFN-γ-induced PD-L1 expression, ↓ STAT3 phosphorylation | [128] |
| Apigenin | HCC | HepG2 HCC | ↓ cell proliferation, ↑ apoptosis, ↑ autophagy, ↓ PI3K/AKT/mTOR pathway | [129] |
|  | leukemia | RBL-2H3 rat leukemia cells | ↓ Lyn, ↓ Syk, ↓ phospholipase Cγ1, ↓ ERK, ↓ JNK, ↓ TNF-α, ↓ IL-4, ↓ IL-5, ↓ IL-6, ↓ IL-13, ↓ COX-2 | [130] |
|  | esophageal cancer | Eca-109 and Kyse-30 human esophageal cancer cells | ↓ IL-6, ↓ cell proliferation, ↑ apoptosis, ↑ PARP, ↑ caspase-8 | [131] |
| Bilberry extracts + isoquercitrin | HCC | PBO-promoted rats | ↓ proliferation, ↓ Ki67, ↓ microsomal ROS, ↓ p-PTEN, ↓ p-AKT, ↓ Smad4, | [132] |
| *Scutellaria* flavonoids (baicalin, baicalein, and wogonin) | non-small cell lung carcinoma | A549 cells and H1299 non-small cell lung carcinoma cells; murine A549 xenografts (six-week-old male Balb/c thymic nude mice) | ↓ Id1, ↑ Rap1-GTP binding, dephosphorylation of AKT and Src | [134] |
| Baicalein and baicalin | liver cancer | SMMC-7721 and HepG2 human liver cancer cells | ↓ PD-L1 expression, ↓ IFN-γ, ↓ STAT3 activity | [135] |
| Sugarcane | colon cancer | SW480 colon cancer cells | ↓ NF-κB phosphorylation, ↓ IL-8 | [136] |
| **Clinical studies** | | | | |
| APG-157 | oral cancer | a double-blind, randomized, placebo-controlled trial; normal subjects (n = 13) and patients with oral cancer (n = 12); two doses of APG-157 (100 mg or 200 mg) were delivered transorally every hour for 3 hours | ↑ antioxidant activity, ↑ anti-inflammatory activity, ↓ IL-1β, ↓ IL-6, ↓ IL-8 | [137] |
| Fisetin | colorectal cancer | a double-blind, randomized placebo-controlled clinical trial, colorectal cancer patients (n = 37) undergoing chemotherapy were assigned to receive either 100 mg fisetin (n = 18) or placebo (n = 19) for seven consecutive weeks | improvement of inflammatory status, ↓ IL-8, ↓ hs-CRP | [138] |
| Green tea | prostate cancer | exploratory, open label, phase II trial; men with prostate cancer (n = 113) were randomized to consume six cups daily of brewed green tea, black tea, or water (control) prior to radical prostatectomy | ↓ NF-κB inflammatory pathway | [139] |
| Soy | breast cancer | randomized, placebo-controlled study; women with early-stage breast cancer (n = 140); soy protein supplementation (n = 70) or placebo (n = 70) for 7 to 30 days | ↑ cell cycle and proliferation, ↑ FGFR2, ↑ E2F5, ↑ BUB1, ↑ CCNB2, ↑ MYBL2, ↑ CDK1, ↑ CDC20 | [141] |

**Abbreviations:** ↑ increased/activated; ↓, decreased/inhibited

,

**
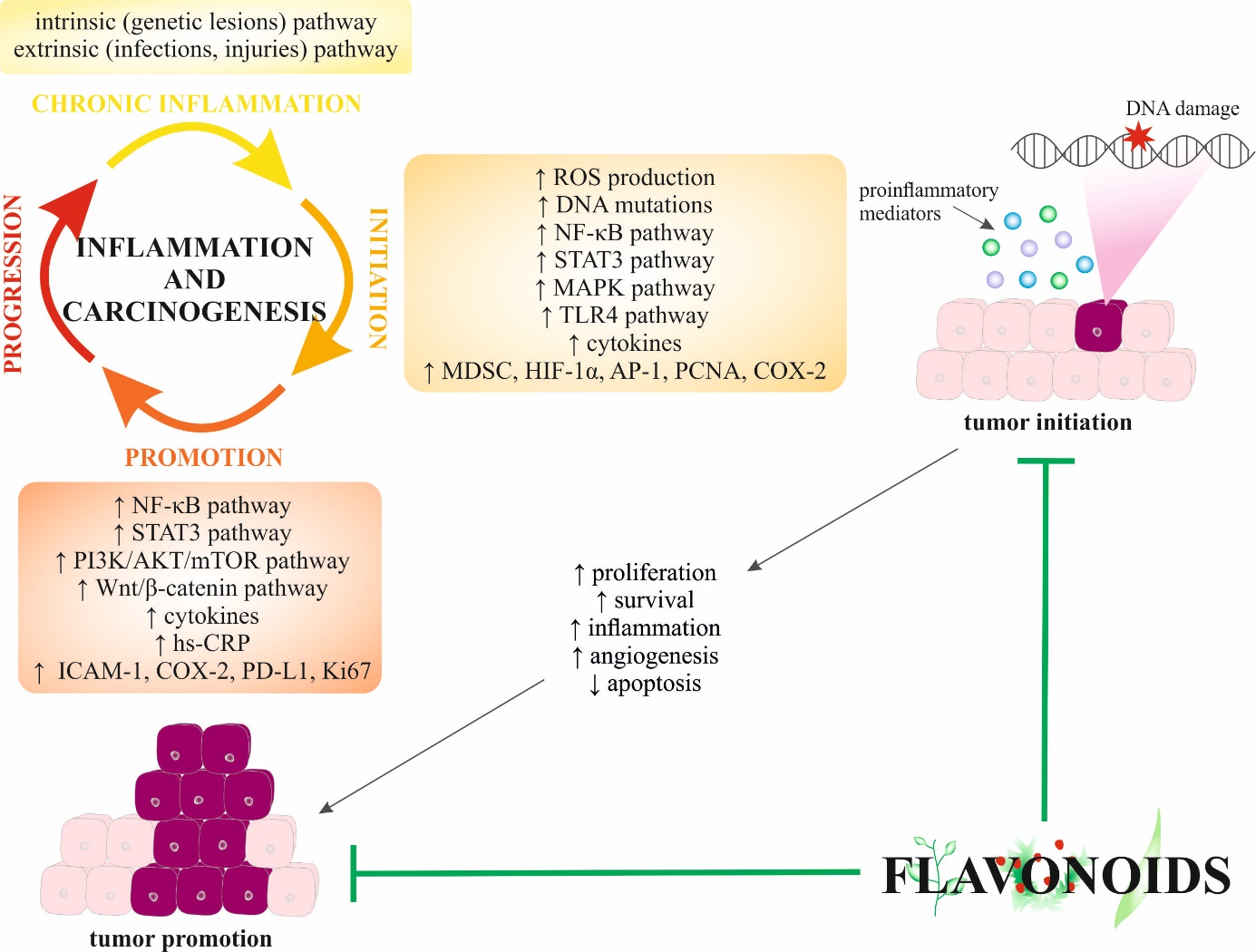
**

**Figure 1. Suppression of tumor initiation and promotion by flavonoids.**

Abbreviations: ↑ increased/activated; ↓, decreased/inhibited

**Flavonoids as anti-inflammatory agents against tumor progression**

As discussed above, cancer progression is a complex process related to a broad spectrum of cellular and molecular signals; however, current research, especially preclinical *in vitro* and *in vivo* analyses, increasingly supports the efficacy of naturally occurring flavonoids against inflammation-associated migration, invasion, and metastasis, which are significant inducers of cancer progression [53,55,78,142,143].

*Preclinical research*

Biochanin A, an O-methylated isoflavone, inhibited the release of pro-inflammatory cytokines TNF-α and IL-6 from A427 lung cancer cells using the cocultured method. Moreover, biochanin A repressed the coculture-stimulated invasion of A427 cells accompanied by the modulation of EMT markers – downregulation of Snail and induction of E-cadherin. Indeed, due to the association between pro-inflammatory responses and tumor metastasis, the administration of biochanin A might alter the TME and thus hinder cancer migration and invasiveness [144]. Similarly, diosmetin isolated from *Dracocephalum peregrinum* L. repressed the migration and invasion of U251 glioma cells *in vitro*, as demonstrated by inhibition of the TGF-β signaling pathway and upregulation of E-cadherin [143]. Also, hesperetin suppressed NF-κB activation and reduced the secretion of pro-inflammatory TNF-α and IL-6 in HepG2 hepatic cancer cells; the anti-inflammatory effects of hesperetin are attributed to reduced ROS overproduction by the Nrf2 pathway [145].

Furthermore, EGCG suppressed the invasion, migration, and metastasis of Panc-1 and MIA PaCa-2 pancreatic cancer cells by the inhibition of the AKT pathway and modulation of EMT markers, specifically upregulated E-cadherin and downregulated N-cadherin and the mesenchymal markers TCF8/ZEB1, β-Catenin, and Vimentin [146]. Moreover, the novel synthetic flavonoid LFG-500 blocked the EMT and metastasis by downregulating YAP activity via ILK in MCF-7 breast cancer and A549 lung cancer cell models of TGF-β-induced EMT [76]. In addition, myricetin inhibited the invasion and migration of radioresistant A549-IR lung cancer cells, as demonstrated by the suppression of MMP-2 and MMP-9 (through FAK-ERK inhibition) and the EMT marker Slug [147]. Similarly, myricetin treatment potently inhibited the cytokine-induced migration and invasion of KKU-100 cholangiocarcinoma cells, mediated partially through STAT3 suppression; myricetin also suppressed downstream target genes of STAT3, including ICAM-1, MMP-9, iNOS, and COX-2 [148]. In addition, the newly synthesized flavonoid derivative GL-V9 suppressed the invasion and migration of HCT116 and SW480 colorectal cancer cells by inhibiting PI3K/AKT and MMP-2/9 signaling [149]. Moreover, baicalein showed anti-metastatic activity in breast cancer *in vitro* and *in vivo* demonstrated by the inhibition of STAT3 activity and suppressed IL-6 [150]. Also, chrysin and daidzein decreased CXCL1 and MMP-9, essential molecules released by the TME that facilitate tumor progression in the rat model of colorectal cancer; therefore, chrysin and daidzein have potential in preventing colorectal cancer angiogenesis and metastasis [151].

Moreover, chrysin inhibited the pro-inflammatory cytokine-induced EMT phenotype and CSC-like characteristics in HeLa cervical cancer cells by blocking the NF-κB/Twist axis [142]. Also, the citrus peel-derived flavonoid tangeretin inhibited breast CSCs formation by suppressing STAT3 and reducing Sox2 levels [152]. In addition, the capacity of 8-bromo-7-methoxychrysin to reverse the M2 polarization of TAMs by inhibiting NF-κB highlights its potential to disrupt interactions between LCSLC and TAMs; indeed, M2 polarization of TAMs in the TME promotes the LCSLC capability of self-renewal [153].

Due to the association between inflammation and angiogenesis in tumor cells, Gong et al. (2018) evaluated the effects of flavonoids in the extract of *Scutellariae* Radix on inflammation-induced angiogenic responses. Eventually, *Scutellariae* Radix extract at various concentrations (0.03, 0.1, 0.3, 1.0 mg/mL) applied for 48 hours decreased pro-inflammatory cytokines IL-1β, IL-6, and TNF-α, and suppressed the expression of the angiogenic biomarkers NF-κB, Cox-2, iNOS, and VEGF in LPS pre-treated cultured macrophage RAW 264.7 cells [154]. In addition, luteolin showed a potent capacity to target HIF-1α/VEGF signaling-mediated EMT and angiogenesis, as demonstrated by EMT suppression (increased E-cadherin and decreased N-cadherin and vimentin) and the downregulation of p-AKT, HIF-1α, VEGF-A, p-VEGFR-2, MMP-2, and MMP-9 in A375 and B16-F10 melanoma cells [155]. Moreover, EGCG and silibinin suppressed the migration of endothelial and lung tumor cells and downregulated VEGF, VEGFR2, and pro-angiogenic members of the miR-17-92 cluster [156].

Also, WCE rich in flavonoids suppressed IL-6, CXCL1, and CXCL8 thus reducing tumor-elicited infiltration MDSCs, TAMs, and endothelial cells accompanied by reduced STAT3 activation, in MDSCs in PC-3 and DU145 prostate cancer xenografts. At the same time, these effects resulted in the inhibition of angiogenesis and metastasis [157]. Furthermore, EGCG attenuated immunosuppression in a 4T1 murine model of breast cancer by decreasing the accumulation of MDSCs and increasing CD4^+^ and CD8^+^ T cell numbers, suggesting that EGCG could effectively enhance the anti-tumor response [78]. Also, limonin decreased inflammation by reducing TNF-α and enhanced the adaptive immune response by promoting the immunophenotyping of CD8, CD4, and CD19 lymphocytes in a Balb/c murine model of colorectal carcinogenesis; limonin thus demonstrated immune-stimulating effects [158]. Figure 2 depicts the anti-inflammatory effects of naturally occurring flavonoids targeting the progression of cancer.





**Figure 2.** **The effectiveness of flavonoids as anti-inflammatory agents against tumor progression**

**Abbreviations:** ↑ increased/activated; ↓, decreased/inhibited

In conclusion, the results of current preclinical research highlight the effectiveness of flavonoids against inflammation-associated cancer progression (Table 3).

**Table 3. Pre-clinical studies demonstrating high effectiveness of flavonoids as anti-inflammatory a**gents against tumor progression

| **Flavonoid** | **Study details** | **Mechanisms** | **Effect** | **Reference** |
| --- | --- | --- | --- | --- |
| Biochanin A | Coculture method - lung cancer A427 cell lines cocultured with monocytic leukemic AML-193 cells | Inhibited TNF-α and IL-6; repressed invasion; altered EMT markers (reduced Snail and increased E-cadherin) | Represses pro-inflammatory responses, invasion, migration, and metastasis | [144] |
| Diosmetin | Glioma U251 cells | Inhibited TGF-β; increased E-cadherin | Inhibited migration and invasion | [143] |
| Hesperetin | Hepatic cancer HepG2 cells | Suppressed NF-κB activation; reduced TNF-α and IL-6; reduced ROS overproduction by the Nrf2 pathway | Reduced inflammatory cytokine secretion | [145] |
| EGCG | Pancreatic cancer Panc-1 and MIA PaCa-2 cells | Inhibited AKT, modulated EMT markers (increased E-cadherin and decreased N-cadherin), decreased mesenchymal markers (TCF8/ZEB1, β-Catenin, and Vimentin) | Suppressed invasion, migration, and metastasis | [146] |
| LFG-500 | TGF-β-induced model of EMT (breast cancer MCF-7 and lung cancer A549 cells) | Downregulation of YAP/ILK axis | Suppressed EMT and metastasis | [76] |
| Myricetin | Radioresistant lung cancer A549-IR cells | Suppressed MMP-2 and MMP-9 through the inhibition of FAK-ERK; decreased Slug | Inhibited invasion and migration | [147] |
| Myricetin | Cholangiocarcinoma KKU-100 cells | Suppressed STAT3 and its target genes ICAM-1, MMP-9, iNOS, and COX-2 | Inhibited cytokine-induced migration and invasion | [148] |
| GL-V9 | Colorectal cancer HCT116 and SW480 cells | Reduced MMP-2 and MMP-9; inhibited PI3K/AKT | Suppressed invasion and migration | [149] |
| Baicalein | Breast cancer model *in vitro* and *in vivo* | Inhibited STAT3 and IL-6 | Reduced metastatic potential | [150] |
| Chrysin and daidzein | Colorectal cancer induced by subcutaneous injection of DMH in male albino rats | Decreased CXCL1 and MMP-9 | Suppressed metastasis and angiogenesis | [151] |
| Chrysin | Cervical cancer HeLa cells co-administered with TNF-α and TGF-β | Blocked the NF-κB/Twist axis | Inhibited pro-inflammatory cytokine-induced EMT phenotype and CSC-like features | [142] |
| Tangeretin | Breast cancer MDA-MB-231 and MCF-7 cells | Suppressed STAT3 and reduced the level of Sox2; reduced transcript levels of stem cell marker genes (Oct3/4, Sox2, Nanog) | Inhibited breast CSCs | [152] |
| 8-bromo-7-methoxychrysin | LCSLC in vitro | Reversed M2 polarization of TAMs (inhibition of NF-κB) | Disrupted the interaction of LCSLCs and TAMs | [153] |
| *Scutellariae* Radix extract | LPS pre-treated cultured RAW 264.7 macrophages | Decreased IL-1β, IL-6, TNF-α, NF-κB, Cox-2, iNOS, and VEGF | Suppressed inflammation-induced angiogenesis | [154] |
| Luteolin | Melanoma A375 and B16-F10 cells | Modulated EMT markers (increased E-cadherin and decreased N-cadherin and vimentin); decreased p-AKT, HIF-1α, VEGF-A, p-VEGFR-2, MMP-2, and MMP-9 | Suppressed HIF-1α/VEGF signaling-mediated EMT and angiogenesis (anti-metastatic effects) | [155] |
| EGCG, silibinin | HUVECs co-cultured with lung cancer A549 cells | Suppressed migration of endothelial and lung tumor cells; downregulated VEGF, VEGFR2, and pro-angiogenic members of the miR-17-92 cluster | Anti-angiogenic efficacy | [156] |
| WCE | Prostate cancer PC-3 and DU145 xenografts | Suppressed IL-6, CXCL1, and CXCL8 → reduced tumor-elicited infiltration of MDSCs, TAMs, and endothelial cells; inhibited STAT3 activation in MDSCs | Inhibited angiogenesis and metastasis | [157] |
| EGCG | Murine 4T1 model of breast cancer | Decreased accumulation of MDSCs; increased CD4^+^ and CD8^+^ T cells | Attenuated immunosuppression | [78] |
| Limonin | Balb/c model of colorectal carcinogenesis | Reduced TNF-α; promoted immunophenotyping of CD8, CD4, and CD19 lymphocytes | Decreased inflammation; enhanced immune responses | [158] |

*Clinical studies*

The above-discussed results of preclinical investigations support the need for further clinical evaluation of flavonoids to target the inflammation-induced progression of cancer. Indeed, Polyphenon E, which contains mainly EGCG and epicatechin, epigallocatechin, and epicatechin-3-gallate in lesser amounts, significantly reduced VEGF and HGF levels in a study of 26 men with positive prostate biopsies scheduled for radical prostatectomy [159]. Moreover, a randomized, phase II trial (conducted on 32 patients with prostate cancer) revealed that consuming bread enriched in soy isoflavones is associated with reduced pro-inflammatory cytokine levels and reduced ratios of T regulatory cells to CD8+ cells and MDSCs in peripheral blood. These results support the potential efficacy of flavonoids in promoting immune surveillance during cancer progression [140]. In addition, the positive effects of lifestyle modifications, including physical activity and diets rich in fruits and vegetables, were demonstrated by a clinical trial that revealed decreased TNF-α levels in BRCA1/2+ breast cancer survivors following a yearlong lifestyle modification program [160].

In conclusion, pre-clinical and clinical investigations point to the potential of flavonoids modulating all stages of the complex process of carcinogenesis associated with inflammation. Obtaining data to enable the implementation of agents precisely targeting specific stages of carcinogenesis will allow the utilization of predictive, preventive, and personalized approaches that increase efficiency and decrease cancer management costs.

**Exemplified 3PM pathways**

As detailed above, non-physiologic inflammation may initiate carcinogenesis and evidently contribute to tumor development and progression as well as it deteriorates individual outcomes in the cohort of cancer patients [161]. The good news is that, due to their evident anti-inflammatory properties, flavonoids are clinically relevant candidates as preventive and therapeutic agents to improve individual outcomes in diseases linked to the non-physiologic inflammation. The challenge is, however, to diagnose well in time an individual predisposition to non-physiologic inflammatory processes such as low-grade chronic inflammation that is definitely the task for predictive diagnostics tools and targeted individualized prevention. Clinical translation is a process of application of preclinical observations and results after clinical validation into medical practice. All described flavonoids targeting specific signaling cascades associated with inflammation are components of different plants, and their synergic/additive effects are associated with supporting human health. The implication of 3PM as a concept of medicine of the 21st century into routine clinical practice is, among other things, based on increasing the amount of flavonoids intake from food due to their beneficial aspect in the prevention of various pathologies. Table 4 links the previously referred preclinical research on some flavonoids to the practical amount of particular food in the daily diet.

**Table 4.** Content of flavonoids in foods

| **Flavonoids** | **Food Source** | **Amounts of flavonoids in food *mg/100g*** | **References** |
| --- | --- | --- | --- |
| (-)-Epicatechin | Apples (raw, with skin) | 15,12 | [162] |
|  | Peaches (raw) | 12,24 | [162] |
|  | Cranberries (raw) | 25,93 | [162] |
|  | Cocoa (dry powder) | 183,49 | [162] |
|  | Red wine (table) | 20,49 | [162] |
| Myricetin | Blueberries | 1,26 | [162] |
|  | Garlic | 1,61 | [162] |
|  | Red wine | 0,83 | [163] |
| Biochanin A | Peanut | 0,06 | [164] |
| Baicalein | Welsh onion | 1,80e-03 | [165] |
| Quercetin-3-O-glucoside | Onions | 21,40 | [162] |
|  | Kale | 22,58 | [162] |
| Daidzein | Tempeh | 22,66 | [166] |
| Tangeretin | Orange juice | 0,3 | [167] |
|  | Soybean | 61,33 | [166] |
| Genistein | Tofu | 12,99 | [166] |
|  | Tempeh | 36,15 | [166] |
|  | Soy milk | 4,94 | [168] |
| Diosmetin 7-O-rutinoside | Lemon – pure juice | 2,92 | [169] |
|  | Peppermint | 95,50 | [169] |
| Hesperetin | Lemon juice | 14,47 | [162] |
|  | Orange juice | 20,39 | [162] |
| Apigenin | Spices, celery seed | 83,70 | [162] |
|  | Peppermint | 8,71 | [162] |
| Luteolin | Green peppers | 4,71 | [162] |
|  | Olive oil | 0,36 | [170] |
|  | Pistachio | 0,10 | [170] |

To this end, individualized profiling based on specialized survey and specific multi-diagnostic patterns, has been demonstrated as instrumental for primary, secondary, and tertiary healthcare exemplified below.

1. **Primary healthcare:** prediction of relevant suboptimal health conditions such as family (genetic) predisposition, abnormal BMI (both overweight and underweight), abnormal stress reactions, disturbed microcirculation, and delayed healing, among others [171–175]; based on anti-inflammatory properties of flavonoids, corresponding individualized mitigating measures may be considered as follows. Healthy and balanced diet rich in naturally occurring phytochemicals including flavonoids is essential to prevent non-communicable diseases associated with non-physiologic inflammation [55,176,177]. In particular, flavonoids show significant efficacy to maintain optimal weight, including less likeliness to be obese [176,178,179]. Preclinical research elaborate the effectiveness of flavonoids to prevent inflammation associated with obesity [180,181]; moreover, these results are supported by clinical studies conducted on specific parts of the population evaluating individual health conditions. Indeed, a randomized clinical trial by Lee et al. (2016) demonstrated the efficacy of anthocyanin-rich black soybeans to reduce abdominal fat and lipid profiles in overweight or obese paticipants [182]. Moreover, anthocyanin exerted beneficial metabolic effects demonstrated through the prevention of insuline resistance in subjects with type 2 diabetes [183]; besides, current evidence highlights the association between cancer and insulin resistance, which is common in obese individuals and type 2 diabetes [184]. Also, flavonoids are efficient agents mitigating chronic stress and improving overall brain health [185,186]. Cocoa products were found to induce anti-inflammatory effects demonstrated through decreased IL-10 and IL-1β in healthy and hypercholesterolaeic individuals [187]. Similarly, results of a double-blinded randomized trial showed the association of high-polyphenol chocolate and increased ICAM-1 in type 2 diabetes when compared with control [188]. The potent capacity of flavonoids in the primary healthcare mediated through multiple mechanisms of action including the anti-inflammatory capacity is demonstrated also by the maintenance of cardiovascular health [189,190] or wound healing [191,192]. Clinical evidence demonstrate honey, a rich source of flavonoids [193], to exert efficacy during healing of wounds and ulcers that had failed to heal using conventional approaches [194]; thus, honey possesses to ability to resolve the inflammatory state of chronic wounds [195]. Also, clinical and epidemiological data support the notion of effectiveness of flavonoids to prevent conditions associated with increased risk of malignant diseases [89,196–198]. Indeed, soy isoflavone was found to increase serum IL-6 in postmenopausal women and thus enhancing the immune surveillance associated with lower incidence of cancer in parts of the world characterized by higher soy intake [199]. Furthermore, mediterranean diet exerted potential beneficiary effects in primary breast cancer prevention [200] preventing all breast cancer subtypes [201], presumably through various anti-cancer mechanisms including anti-inflammatory activity. Therefore, naturally occurring flavonoids and flavonoid-rich plant food represent potent agents in primary healthcare concerned with individual health conditions or specific disease predisposition associated with the risk of cancer mediated through their anti-inflammatory capacity supported by cost-effectiveness and high efficacy.
2. **Secondary healthcare:** prediction of tumor progression and increased risk of pre-metastatic niches/metastatic disease [4,202,203]; as mentioned above, flavonoids were comprehensively documented to prevent the onset of the cancer invasiveness in preclinical research via the modulation of numerous signaling pathways involved in critical steps of metastatic spread. In addition, flavonoids were described to be effective oncostatic substances in highly aggressive cancer models including various *in vivo* approaches. Regarding oncology practice, flavonoids demonstrated promising results applied in the combination with conventional chemotherapeutics in metastatic cancer disease. However, in-depth analyses of re-sensitizing cancer cells by flavonoids towards conventional chemotherapy and assessing the activities of flavonoids on cancer stem cells survival, affecting the relapse and multidrug resistance are needed [55].

There are only limited clinical data evaluating the effectiveness of flavonoids against advanced cancer disease. Curcumin has been documented to suppress cancer cells due to its anti-inflammatory and antioxidant effects. On the other hand, its effectiveness is limited by poor absorption after oral administration. A botanical drug APG-157 containing multiple polyphenols, including flavonoids, demonstrated improved bioavailability and clinical activity in patients with oral cancer. APG-157 was well absorbed, reduced parameters of inflammation, and upregulated expression of genes linked with differentiation and T-cell recruitment to the TME. These data shows the potential using of APG-157 in combination with anti-cancer therapies including advanced disease [137]. In another clinical study, fisetin reduced the plasma levels of IL-8, hs-CRP, and MMP-7 levels (p < 0.02) [138]. Green tea and its constituents, mainly EGCG, show anti-inflammatory activities associated with reduced VEGF and HGF levels in prostate cancer patients [139,159]. Oral administration of soy isoflavone enriched bread significantly suppressed proinflammatory cytokines and immunosuppressive cells in men with prostate cancer [140]. Finally, lifestyle modifications in BRCA1/2+ breast cancer survivors, including physical activity and diets rich in flavonoids (in fruits and vegetables) revealed decreased TNF-α levels [160]. All these mentioned clinical data suggest anti-metastatic potential of fisetin, green tea, soy, and fruit and vegetrables rich in flavonoids in cancer patients. Based on preclinical research, there are numerous studies demonstrating high effectiveness of flavonoids and flavonoid rich extracts as anti-inflammatory agents against tumor progression. These include: biochanin A, diosmetin, hesperetin, EGCG, synthetic flavonoids LFG-500 and GL-V9, myricetin, baicalein, chrysin, daidzein, tangeretin, 8-bromo-7-methoxychrysin, *Scutellariae* Radix extract, luteolin, silibinin, WCE, and limonin (see Table 3).

1. **Tertiary healthcare:** prediction in palliative care [204]; in this regard, flavonoids were described to block an activation of numerous cellular regulatory proteins such as cytokines and transcription factors involved in cellular inflammatory responses and pain. From the clinical point of view, it could be very beneficial to develop protective delivery formulations containing flavonoids to treat inflammation and pain. Flavonoids suppress the expression of wide spectrum of inflammatory molecules such as NO, TNF-α, IL-1β, and COX-2, down-regulate ICAM-1 and VEGF synthesis, moreover, activate STAT3, NF-kB, NLRP3 inflammasome, and finally MAPK cellular pathways. Based on mentioned multi-target activities of flavonoids, they have great potential in clinical sphere, including oncology practice, due to their anti-inflammatory and analgesic properties [205].

There are several examples of specific flavonoids that shold be beneficial in palliative care in cancer patients. In the case report, the breast cancer patient showed progressive liver failure despite several chemotherapy treatments, including paclitaxel, capecitabine, and vinorelbine. Silibinin application improved hepatic failure due to extensive liver infiltration in this patient. After the initiation of therapy, the patient showed clinical and liver improvements that permitted the continuation of palliative chemotherapy [206]. Harati et al. (2017) documented that EGCG and silibinin represent potential candidate molecules as mild therapeutic options for patients with solid sarcomas that require palliative treatment and are not suitable for doxorubicin‑based chemotherapy [207]. In another preclinical study, baicalelin and 6-gingerol, reduced 5-fluorouracil-induced overexpression of CXCL1 in the colon and prevented the development of neutrophil recruitment and weaken diarrhea development by the suppression of NF-κB activity [208]. Data from another preclinical study point out to protective role of quercetin coadministered with vitamin E in the prevention of doxorubicin-induced toxicity in uterine and ovarian tissues in rats [209]. The administration of casticin in male rats demonstrated a palliative effects against cisplatin-induced renal damage and recovered all renal parameters to normal levels [210].

With exception of cancer, there are also other medicine problems/diagnosis where flavonoids show palliative effects. Using mouse model, EGCG significantly reduced osteoarthritis disease progression and exerts palliative effects [211]. Kaswan et al. (2021) described that the serotonergic pathway (via the 5-HT1A receptor subtype in the central nervous system) is essential for cardamonin to suppress neuropathic pain in chronic constriction injury-induced neuropathic pain animal model [212]. EGCG administration modulating the Wnt/β-catenin signaling pathways reduced postoperative pain related to inflammatory and neurochemical alterations [213]. Most of the above data are from preclinical research; therefore well-controlled clinical studies are needed to validate the positive effects of flavonoids in palliative care in patients with cancer as well as other diseases.

**Strengths and limitations**

Phytochemicals represent naturally occuring anti-cancer agents affecting numerous cellular pathways. However, the utilization of flavonoids in clinical practice to prevent or treat cancer still faces difficulties associated with limitations of up-to-date studies. The efficacy of flavonoids as anti-inflammatory agents *in vivo* is highly associated with its bioavailability and bioactivity that is affected by various factors on the side of the individual recipient or properties of the flavonoid itself including low absorption, an extensive metabolization, rapid elimination, or structural complexity of flavonoids within subclasses [55]. For example, catechins appear to be absorbed in amount smaller than intake due to rapid metabolization [214]. Nevertheless, an increase in the bioavailablity of flavonoids is highly recommended interest of current research [215]. Moreover, complexity and chronicity as basic characteristcs of plenty of human diseases as well as sex, age, comorbidities, genetic similarity, and environmental factors are frequently not appropriately modeled in research [216].

The above-mentioned limitations associated with the evaluation of anti-cancer effects of phytochemicals *in vitro* and *in vivo* are, further, exemplified below. Preclinical *in vitro* studies demonstrate potent capacity of soy isoflavones to suppress prostate carcinogenesis [217]. However, as stated by Miltyk et al. (2003), despite the capacity of isoflavone to induce genetic damage of prostate cancer cells *in vitro*, similar effects were not observed in human subjects [218]. Similarly, isoflavones exerted no effects on markers of inflammation [219] and had no effect on prostate-specific antigen or hormone levels in prostate cancer patients [220]. However, the evaluation of other doses and duration of the administration in further research of anti-cancer efficacy of isoflavones in prostate cancer could bring different results [219].

On the contrary, the preventive efficacy of EGCG was supported by the detection of methylated and nonmethylated forms of EGCG in prostate tissue after short-term green tea intervention [221]. Also, potent capacity of a mixture of natural agents to protect human lymphocytes *in vitro* in comparison with single agents [222] was further emphasized by a proof-of-concept study in humans *in vivo* [223]. Moreover, phytochemicals, for example tannins, chelate metal ions that generate ROS and thus stabilize potential prooxidant activity [224]. Therefore, despite limitations, phytochemicals still seem to be efficient as anti-cancer agents in preclinical *in vivo* [225] and clinical research [159,225]. Nevertheless, a patient-tailored approach requires the choice of the right agent and dosage at the right time associated with individualization and standardization of healthcare [216]. In conclusion, insufficient number of human studies in comparison with preclinical evaluations highlights the need for intensive clinical research focused more on the patient as an individual’s, specific characteristics of the disease, and properties of the agent enabling the full utilization of the potential phytochemicals in the terms of 3PM.

**Authors’** **contribution**: P.K. and O.G. were responsible for the paper conception and final data interpretation and presentation. The manuscript was drafted by P.K., A.L., L.K., M.S. (Marek Samec), K.K., D.V. and critically revised by P.K., K.B., K.Z., A.B., R.I. Figures were prepared by M.S. (Marek Samec), L.K., and A.L. P.K., O.G., M.S. (Mehdi Shakibaei), and D.B. provided skilled assistance and supervised the overall preparation of the manuscript. All authors have read and agreed to the published version of the manuscript.

**Funding**: The present study was supported by the Scientific Grant Agency of the Ministry of Education, Science, Research and Sport of the Slovak Republic (Bratislava, Slovak Republic; grant no. VEGA 1/0136/19), Slovak Research and Development Agency under Contract No. APVV-16-0021, and by the LISPER project (grant Nr. 313011V446) in bilateral agreement with the European Association for Predictive, Preventive and Personalised Medicine. D. B. was supported by a National Priorities Research Program grant (NPRP 11S-1214-170101) from the Qatar National Research Fund (QNRF, a member of Qatar Foundation).

## **Data availability**

## Not applicable.

## **Code availability**

## Not applicable.

## **Declarations**

**Ethics approval**

Not applicable.

**Consent to participate**

Not applicable.

**Consent for publication**

Not applicable.

**Conflict of interest**

The authors declare no competing interests.

**References**

1. De Pasquale, C.; Campana, S.; Bonaccorsi, I.; Carrega, P.; Ferlazzo, G. ILC in Chronic Inflammation, Cancer and Targeting with Biologicals. *Molecular Aspects of Medicine* **2021**, *80*, 100963, doi:10.1016/j.mam.2021.100963.

2. Awad, R.M.; De Vlaeminck, Y.; Maebe, J.; Goyvaerts, C.; Breckpot, K. Turn Back the TIMe: Targeting Tumor Infiltrating Myeloid Cells to Revert Cancer Progression. *Front Immunol* **2018**, *9*, 1977, doi:10.3389/fimmu.2018.01977.

3. Koklesova, L.; Liskova, A.; Samec, M.; Qaradakhi, T.; Zulli, A.; Smejkal, K.; Kajo, K.; Jakubikova, J.; Behzadi, P.; Pec, M.; et al. Genoprotective Activities of Plant Natural Substances in Cancer and Chemopreventive Strategies in the Context of 3P Medicine. *EPMA Journal* **2020**, *11*, 261–287, doi:10.1007/s13167-020-00210-5.

4. Crigna, A.T.; Samec, M.; Koklesova, L.; Liskova, A.; Giordano, F.A.; Kubatka, P.; Golubnitschaja, O. Cell-Free Nucleic Acid Patterns in Disease Prediction and Monitoring-Hype or Hope? *EPMA J* **2020**, 1–25, doi:10.1007/s13167-020-00226-x.

5. Shimizu, K.; Iyoda, T.; Okada, M.; Yamasaki, S.; Fujii, S. Immune Suppression and Reversal of the Suppressive Tumor Microenvironment. *International Immunology* **2018**, *30*, 445–455, doi:10.1093/intimm/dxy042.

6. Rossi, J.-F.; Lu, Z.Y.; Massart, C.; Levon, K. Dynamic Immune/Inflammation Precision Medicine: The Good and the Bad Inflammation in Infection and Cancer. *Front Immunol* **2021**, *12*, 595722, doi:10.3389/fimmu.2021.595722.

7. Hussain, S.P.; Harris, C.C. Inflammation and Cancer: An Ancient Link with Novel Potentials. *Int. J. Cancer* **2007**, *121*, 2373–2380, doi:10.1002/ijc.23173.

8. Greten, F.R.; Grivennikov, S.I. Inflammation and Cancer: Triggers, Mechanisms, and Consequences. *Immunity* **2019**, *51*, 27–41, doi:10.1016/j.immuni.2019.06.025.

9. Gupta, S.C.; Kunnumakkara, A.B.; Aggarwal, S.; Aggarwal, B.B. Inflammation, a Double-Edge Sword for Cancer and Other Age-Related Diseases. *Front Immunol* **2018**, *9*, 2160, doi:10.3389/fimmu.2018.02160.

10. Petts, D.; Wren, M.; Nation, B.; Guthrie, G.; Kyle, B.; Peters, L.; Mortlock, S.; Clarke, S.; Burt, C. A SHORT HISTORY OF OCCUPATIONAL DISEASE: 2. ASBESTOS, CHEMICALS, RADIUM AND BEYOND. *Ulster Med J* **2021**, *90*, 32–34.

11. Stone, T.W.; McPherson, M.; Gail Darlington, L. Obesity and Cancer: Existing and New Hypotheses for a Causal Connection. *EBioMedicine* **2018**, *30*, 14–28, doi:10.1016/j.ebiom.2018.02.022.

12. Bousquenaud, M.; Fico, F.; Solinas, G.; Rüegg, C.; Santamaria-Martínez, A. Obesity Promotes the Expansion of Metastasis-Initiating Cells in Breast Cancer. *Breast Cancer Res* **2018**, *20*, 104, doi:10.1186/s13058-018-1029-4.

13. Ding, S.; Jiang, H.; Fang, J. Regulation of Immune Function by Polyphenols. *Journal of Immunology Research* **2018**, *2018*, e1264074, doi:10.1155/2018/1264074.

14. Yahfoufi, N.; Alsadi, N.; Jambi, M.; Matar, C. The Immunomodulatory and Anti-Inflammatory Role of Polyphenols. *Nutrients* **2018**, *10*, doi:10.3390/nu10111618.

15. Kopustinskiene, D.M.; Jakstas, V.; Savickas, A.; Bernatoniene, J. Flavonoids as Anticancer Agents. *Nutrients* **2020**, *12*, doi:10.3390/nu12020457.

16. Anuja, K.; Roy, S.; Ghosh, C.; Gupta, P.; Bhattacharjee, S.; Banerjee, B. Prolonged Inflammatory Microenvironment Is Crucial for Pro-Neoplastic Growth and Genome Instability: A Detailed Review. *Inflamm Res* **2017**, *66*, 119–128, doi:10.1007/s00011-016-0985-3.

17. Rozhok, A.; DeGregori, J. A Generalized Theory of Age-Dependent Carcinogenesis. *eLife* **2019**, *8*, e39950, doi:10.7554/eLife.39950.

18. To, E.E.; O’Leary, J.J.; O’Neill, L.A.J.; Vlahos, R.; Bozinovski, S.; Porter, C.J.H.; Brooks, R.D.; Brooks, D.A.; Selemidis, S. Spatial Properties of Reactive Oxygen Species Govern Pathogen-Specific Immune System Responses. *Antioxid Redox Signal* **2020**, *32*, 982–992, doi:10.1089/ars.2020.8027.

19. Liskova Alena; Samec Marek; Koklesova Lenka; Kudela Erik; Kubatka Peter; Golubnitschaja Olga Mitochondriopathies as a Clue to Systemic Disorders: “Vicious Circle” of Mitochondrial Injury, Analytical Tools and Mitigating Measures in Context of Predictive, Preventive, and Personalized (3P) Medicine. **2021**, *22*, 2007, doi:10.3390/ijms22042007.

20. Aceto, G.M.; Catalano, T.; Curia, M.C. Molecular Aspects of Colorectal Adenomas: The Interplay among Microenvironment, Oxidative Stress, and Predisposition. *Biomed Res Int* **2020**, *2020*, 1726309, doi:10.1155/2020/1726309.

21. de Barrios, O.; Sanchez-Moral, L.; Cortés, M.; Ninfali, C.; Profitós-Pelejà, N.; Martínez-Campanario, M.C.; Siles, L.; Del Campo, R.; Fernández-Aceñero, M.J.; Darling, D.S.; et al. ZEB1 Promotes Inflammation and Progression towards Inflammation-Driven Carcinoma through Repression of the DNA Repair Glycosylase MPG in Epithelial Cells. *Gut* **2019**, *68*, 2129–2141, doi:10.1136/gutjnl-2018-317294.

22. Gobert, A.P.; Wilson, K.T. Polyamine- and NADPH-Dependent Generation of ROS during Helicobacter Pylori Infection: A Blessing in Disguise. *Free Radic Biol Med* **2017**, *105*, 16–27, doi:10.1016/j.freeradbiomed.2016.09.024.

23. du Plessis, M.; Davis, T.; Loos, B.; Pretorius, E.; de Villiers, W.J.S.; Engelbrecht, A.M. Molecular Regulation of Autophagy in a Pro-Inflammatory Tumour Microenvironment: New Insight into the Role of Serum Amyloid A. *Cytokine Growth Factor Rev* **2021**, *59*, 71–83, doi:10.1016/j.cytogfr.2021.01.007.

24. Wang, K.; Kim, M.K.; Di Caro, G.; Wong, J.; Shalapour, S.; Wan, J.; Zhang, W.; Zhong, Z.; Sanchez-Lopez, E.; Wu, L.-W.; et al. Interleukin-17 Receptor A Signaling in Transformed Enterocytes Promotes Early Colorectal Tumorigenesis. *Immunity* **2014**, *41*, 1052–1063, doi:10.1016/j.immuni.2014.11.009.

25. Beneforti, L.; Dander, E.; Bresolin, S.; Bueno, C.; Acunzo, D.; Bertagna, M.; Ford, A.; Gentner, B.; Kronnie, G. te; Vergani, P.; et al. Pro-Inflammatory Cytokines Favor the Emergence of ETV6-RUNX1-Positive Pre-Leukemic Cells in a Model of Mesenchymal Niche. *British Journal of Haematology* **2020**, *190*, 262–273, doi:10.1111/bjh.16523.

26. Huang, X.; Tian, Y.; Shi, W.; Chen, J.; Yan, L.; Ren, L.; Zhang, X.; Zhu, J. Role of Inflammation in the Malignant Transformation of Pleural Mesothelial Cells Induced by Multi-Walled Carbon Nanotubes. *Nanotoxicology* **2020**, *14*, 947–967, doi:10.1080/17435390.2020.1777477.

27. Yoshida, G.J. Regulation of Heterogeneous Cancer-Associated Fibroblasts: The Molecular Pathology of Activated Signaling Pathways. *J Exp Clin Cancer Res* **2020**, *39*, 112, doi:10.1186/s13046-020-01611-0.

28. Voronov, E.; Apte, R.N. Targeting the Tumor Microenvironment by Intervention in Interleukin-1 Biology. *Curr Pharm Des* **2017**, *23*, 4893–4905, doi:10.2174/1381612823666170613080919.

29. D’Orazi, G.; Cordani, M.; Cirone, M. Oncogenic Pathways Activated by Pro-Inflammatory Cytokines Promote Mutant P53 Stability: Clue for Novel Anticancer Therapies. *Cell Mol Life Sci* **2021**, *78*, 1853–1860, doi:10.1007/s00018-020-03677-7.

30. Ju, X.; Zhang, H.; Zhou, Z.; Chen, M.; Wang, Q. Tumor-Associated Macrophages Induce PD-L1 Expression in Gastric Cancer Cells through IL-6 and TNF-ɑ Signaling. *Experimental Cell Research* **2020**, *396*, 112315, doi:10.1016/j.yexcr.2020.112315.

31. Tang, X.; Cao, T.; Zhu, Y.; Zhang, L.; Chen, J.; Liu, T.; Ming, X.; Fang, S.; Yuan, Y.; Jiang, L.; et al. PIM2 Promotes Hepatocellular Carcinoma Tumorigenesis and Progression through Activating NF-ΚB Signaling Pathway. *Cell Death Dis* **2020**, *11*, 1–14, doi:10.1038/s41419-020-2700-0.

32. Muoio, M.G.; Talia, M.; Lappano, R.; Sims, A.H.; Vella, V.; Cirillo, F.; Manzella, L.; Giuliano, M.; Maggiolini, M.; Belfiore, A.; et al. Activation of the S100A7/RAGE Pathway by IGF-1 Contributes to Angiogenesis in Breast Cancer. *Cancers (Basel)* **2021**, *13*, 621, doi:10.3390/cancers13040621.

33. Morris, J.; Gonzales, C.B.; De La Chapa, J.J.; Cabang, A.B.; Fountzilas, C.; Patel, M.; Orozco, S.; Wargovich, M.J. The Highly Pure Neem Leaf Extract, SCNE, Inhibits Tumorigenesis in Oral Squamous Cell Carcinoma via Disruption of Pro-Tumor Inflammatory Cytokines and Cell Signaling. *Front Oncol* **2019**, *9*, 890, doi:10.3389/fonc.2019.00890.

34. Deng, X.-X.; Jiao, Y.-N.; Hao, H.-F.; Xue, D.; Bai, C.-C.; Han, S.-Y. Taraxacum Mongolicum Extract Inhibited Malignant Phenotype of Triple-Negative Breast Cancer Cells in Tumor-Associated Macrophages Microenvironment through Suppressing IL-10 / STAT3 / PD-L1 Signaling Pathways. *J Ethnopharmacol* **2021**, *274*, 113978, doi:10.1016/j.jep.2021.113978.

35. Zhang, X.; Zeng, Y.; Qu, Q.; Zhu, J.; Liu, Z.; Ning, W.; Zeng, H.; Zhang, N.; Du, W.; Chen, C.; et al. PD-L1 Induced by IFN-γ from Tumor-Associated Macrophages via the JAK/STAT3 and PI3K/AKT Signaling Pathways Promoted Progression of Lung Cancer. *Int J Clin Oncol* **2017**, *22*, 1026–1033, doi:10.1007/s10147-017-1161-7.

36. Kim, E.K.; Choi, E.-J. Compromised MAPK Signaling in Human Diseases: An Update. *Arch Toxicol* **2015**, *89*, 867–882, doi:10.1007/s00204-015-1472-2.

37. Pópulo, H.; Lopes, J.M.; Soares, P. The MTOR Signalling Pathway in Human Cancer. *Int J Mol Sci* **2012**, *13*, 1886–1918, doi:10.3390/ijms13021886.

38. Zhao, X.; Lin, Y.; Jiang, B.; Yin, J.; Lu, C.; Wang, J.; Zeng, J. Icaritin Inhibits Lung Cancer-Induced Osteoclastogenesis by Suppressing the Expression of IL-6 and TNF-a and through AMPK/MTOR Signaling Pathway. *Anticancer Drugs* **2020**, *31*, 1004–1011, doi:10.1097/CAD.0000000000000976.

39. Varga, J.; Greten, F.R. Cell Plasticity in Epithelial Homeostasis and Tumorigenesis. *Nat Cell Biol* **2017**, *19*, 1133–1141, doi:10.1038/ncb3611.

40. Chen, X.-J.; Wei, W.-F.; Wang, Z.-C.; Wang, N.; Guo, C.-H.; Zhou, C.-F.; Liang, L.-J.; Wu, S.; Liang, L.; Wang, W. A Novel Lymphatic Pattern Promotes Metastasis of Cervical Cancer in a Hypoxic Tumour-Associated Macrophage-Dependent Manner. *Angiogenesis* **2021**, doi:10.1007/s10456-020-09766-2.

41. Joshi, R.S.; Kanugula, S.S.; Sudhir, S.; Pereira, M.P.; Jain, S.; Aghi, M.K. The Role of Cancer-Associated Fibroblasts in Tumor Progression. *Cancers (Basel)* **2021**, *13*, 1399, doi:10.3390/cancers13061399.

42. Calon, A.; Lonardo, E.; Berenguer-Llergo, A.; Espinet, E.; Hernando-Momblona, X.; Iglesias, M.; Sevillano, M.; Palomo-Ponce, S.; Tauriello, D.V.F.; Byrom, D.; et al. Stromal Gene Expression Defines Poor-Prognosis Subtypes in Colorectal Cancer. *Nat Genet* **2015**, *47*, 320–329, doi:10.1038/ng.3225.

43. Hong, D.; Fritz, A.J.; Zaidi, S.K.; van Wijnen, A.J.; Nickerson, J.A.; Imbalzano, A.N.; Lian, J.B.; Stein, J.L.; Stein, G.S. Epithelial-to-Mesenchymal Transition and Cancer Stem Cells Contribute to Breast Cancer Heterogeneity. *J Cell Physiol* **2018**, *233*, 9136–9144, doi:10.1002/jcp.26847.

44. Adekoya, T.O.; Richardson, R.M. Cytokines and Chemokines as Mediators of Prostate Cancer Metastasis. *Int J Mol Sci* **2020**, *21*, E4449, doi:10.3390/ijms21124449.

45. Powell, I.J.; Chinni, S.R.; Reddy, S.S.; Zaslavsky, A.; Gavande, N. Pro-Inflammatory Cytokines and Chemokines Initiate Multiple Prostate Cancer Biologic Pathways of Cellular Proliferation, Heterogeneity and Metastasis in a Racially Diverse Population and Underlie the Genetic/Biologic Mechanism of Racial Disparity: Update. *Urologic Oncology: Seminars and Original Investigations* **2021**, *39*, 34–40, doi:10.1016/j.urolonc.2020.08.019.

46. Liongue, C.; Ward, A.C.; Duan, W.; Shigdar, S. Cytokine Networks and Cancer Stem Cells. In *Cancer Stem Cells: Emerging Concepts and Future Perspectives in Translational Oncology*, 1st ed. Babashah, S., Ed.; Publisher: Springer International Publishing: Cham, 2015; pp. 67–87 ISBN 978-3-319-21030-8.

47. Zhang, S.; Yang, X.; Wang, L.; Zhang, C. Interplay between Inflammatory Tumor Microenvironment and Cancer Stem Cells. *Oncol Lett* **2018**, *16*, 679–686, doi:10.3892/ol.2018.8716.

48. Fu, L.-Q.; Du, W.-L.; Cai, M.-H.; Yao, J.-Y.; Zhao, Y.-Y.; Mou, X.-Z. The Roles of Tumor-Associated Macrophages in Tumor Angiogenesis and Metastasis. *Cellular Immunology* **2020**, *353*, 104119, doi:10.1016/j.cellimm.2020.104119.

49. Horiguchi, H.; Tsujimoto, H.; Shinomiya, N.; Matsumoto, Y.; Sugasawa, H.; Yamori, T.; Miyazaki, H.; Saitoh, D.; Kishi, Y.; Ueno, H. A Potential Role of Adhesion Molecules on Lung Metastasis Enhanced by Local Inflammation. *Anticancer Res* **2020**, *40*, 6171–6178, doi:10.21873/anticanres.14637.

50. Xiong, J.; Wang, H.; Wang, Q. Suppressive Myeloid Cells Shape the Tumor Immune Microenvironment. *Advanced Biology* **2021**, *5*, 1900311, doi:10.1002/adbi.201900311.

51. Parker, K.H.; Beury, D.W.; Ostrand-Rosenberg, S. Chapter Three - Myeloid-Derived Suppressor Cells: Critical Cells Driving Immune Suppression in the Tumor Microenvironment. In *Advances in Cancer Research*; Wang, X.-Y., Fisher, P.B., Eds.; Immunotherapy of Cancer; Academic Press, 2015; Vol. 128, pp. 95–139, doi: 10.1016/bs.acr.2015.04.002.

52. Samec, M.; Liskova, A.; Koklesova, L.; Mersakova, S.; Strnadel, J.; Kajo, K.; et al.; Zhai, K.; Smejkal, K.; Mirzaei, S.; et al. Flavonoids Targeting HIF-1: Implications on Cancer Metabolism. *Cancers* **2021**, *13*, 130, doi:10.3390/cancers13010130.

53. Liskova, A.; Koklesova, L.; Samec, M.; Varghese, E.; Abotaleb, M.; Samuel, S.M.; et al.; Biringer, K.; Petras, M.; Blahutova, D.; et al. Implications of Flavonoids as Potential Modulators of Cancer Neovascularity. *J Cancer Res Clin Oncol* **2020**, doi:10.1007/s00432-020-03383-8.

54. Samec, M.; Liskova, A.; Koklesova, L.; Samuel, S.M.; Zhai, K.; Buhrmann, C.; Varghese, E.; Abotaleb, M.; Qaradakhi, T.; Zulli, A.; et al. Flavonoids against the Warburg Phenotype—Concepts of Predictive, Preventive and Personalised Medicine to Cut the Gordian Knot of Cancer Cell Metabolism. *EPMA Journal* **2020**, *11*, 377–398, doi:10.1007/s13167-020-00217-y.

55. Liskova, A.; Koklesova, L.; Samec, M.; Smejkal, K.; Samuel, S.M.; Varghese, E.; Abotaleb, M.; Biringer, K.; Kudela, E.; Danko, J.; et al. Flavonoids in Cancer Metastasis. *Cancers (Basel)* **2020**, *12*, doi:10.3390/cancers12061498.

56. Siddiqui, M.; Abdellatif, B.; Zhai, K.; Liskova, A.; Kubatka, P.; Büsselberg, D. Flavonoids Alleviate Peripheral Neuropathy Induced by Anticancer Drugs. *Cancers (Basel)* **2021**, *13*, 1576, doi:10.3390/cancers13071576.

57. Liskova, A.; Samec, M.; Koklesova, L.; Samuel, S.M.; Zhai, K.; Al-Ishaq, R.K.; Abotaleb, M.; Nosal, V.; Kajo, K.; Ashrafizadeh, M.; et al. Flavonoids against the SARS-CoV-2 Induced Inflammatory Storm. *Biomedicine & Pharmacotherapy* **2021**, *138*, 111430, doi:10.1016/j.biopha.2021.111430.

58. Al-Ishaq, R.K.; Abotaleb, M.; Kubatka, P.; Kajo, K.; Büsselberg, D. Flavonoids and Their Anti-Diabetic Effects: Cellular Mechanisms and Effects to Improve Blood Sugar Levels. *Biomolecules* **2019**, *9*, E430, doi:10.3390/biom9090430.

59. Abotaleb, M.; Samuel, S.; Varghese, E.; Varghese, S.; Kubatka, P.; Liskova, A.; Büsselberg, D. Flavonoids in Cancer and Apoptosis. *Cancers* **2018**, *11*, 28, doi:10.3390/cancers11010028.

60. Kubatka, P.; Kello, M.; Kajo, K.; Kruzliak, P.; Výbohová, D.; Šmejkal, K.; et al.; Zulli, A.; Gönciová, G.; Mojžiš, J.; et al. Young Barley Indicates Antitumor Effects in Experimental Breast Cancer In Vivo and In Vitro. *Nutr Cancer* **2016**, *68*, 611–621, doi:10.1080/01635581.2016.1154577.

61. Kubatka, P.; Kapinová, A.; Kello, M.; Kruzliak, P.; Kajo, K.; Výbohová, D.; et al.; Murin, R.; Viera, T.; Mojžiš, J.; et al. Fruit Peel Polyphenols Demonstrate Substantial Anti-Tumour Effects in the Model of Breast Cancer. *Eur J Nutr* **2016**, *55*, 955–965, doi:10.1007/s00394-015-0910-5.

62. Wang, Q.; Xie, C.; Xi, S.; Qian, F.; Peng, X.; Huang, J.; Tang, F. Radioprotective Effect of Flavonoids on Ionizing Radiation-Induced Brain Damage. *Molecules* **2020**, *25*, E5719, doi:10.3390/molecules25235719.

63. Ahn-Jarvis, J.H.; Parihar, A.; Doseff, A.I. Dietary Flavonoids for Immunoregulation and Cancer: Food Design for Targeting Disease. *Antioxidants (Basel)* **2019**, *8*, 202, doi:10.3390/antiox8070202.

64. Leyva-López, N.; Gutierrez-Grijalva, E.P.; Ambriz-Perez, D.L.; Heredia, J.B. Flavonoids as Cytokine Modulators: A Possible Therapy for Inflammation-Related Diseases. *Int J Mol Sci* **2016**, *17*, 921, doi:10.3390/ijms17060921.

65. Bauer, D.; Redmon, N.; Mazzio, E.; Soliman, K.F. Apigenin Inhibits TNFα/IL-1α-Induced CCL2 Release through IKBK-Epsilon Signaling in MDA-MB-231 Human Breast Cancer Cells. *PLoS One* **2017**, *12*, e0175558, doi:10.1371/journal.pone.0175558.

66. Ting, H.; Deep, G.; Kumar, S.; Jain, A.K.; Agarwal, C.; Agarwal, R. Beneficial Effects of the Naturally Occurring Flavonoid Silibinin on the Prostate Cancer Microenvironment: Role of Monocyte Chemotactic Protein-1 and Immune Cell Recruitment. *Carcinogenesis* **2016**, *37*, 589–599, doi:10.1093/carcin/bgw039.

67. Ting, H.J.; Deep, G.; Jain, A.K.; Cimic, A.; Sirintrapun, J.; Romero, L.M.; Cramer, S.D.; Agarwal, C.; Agarwal, R. Silibinin Prevents Prostate Cancer Cell-Mediated Differentiation of Naïve Fibroblasts into Cancer-Associated Fibroblast Phenotype by Targeting TGF Β2. *Mol Carcinog* **2015**, *54*, 730–741, doi:10.1002/mc.22135.

68. Hou, S.; Yuan, Q.; Yu, N.; Liu, B.; Huang, G.; Yuan, X. Cardamonin Attenuates Chronic Inflammation and Tumorigenesis in Colon. *Cell Cycle* **2019**, *18*, 3275–3287, doi:10.1080/15384101.2019.1673620.

69. Khan, H.; Ullah, H.; Castilho, P.C.M.F.; Gomila, A.S.; D’Onofrio, G.; Filosa, R.; Wang, F.; Nabavi, S.M.; Daglia, M.; Silva, A.S.; et al. Targeting NF-ΚB Signaling Pathway in Cancer by Dietary Polyphenols. *Crit Rev Food Sci Nutr* **2020**, *60*, 2790–2800, doi:10.1080/10408398.2019.1661827.

70. Zhao, S.; Jiang, Y.; Zhao, J.; Li, H.; Yin, X.; Wang, Y.; Xie, Y.; Chen, X.; Lu, J.; Dong, Z.; et al. Quercetin-3-Methyl Ether Inhibits Esophageal Carcinogenesis by Targeting the AKT/MTOR/P70S6K and MAPK Pathways. *Mol Carcinog* **2018**, *57*, 1540–1552, doi:10.1002/mc.22876.

71. Jiang, M.; Zhou, L.-Y.; Xu, N.; An, Q. Hydroxysafflor Yellow A Inhibited Lipopolysaccharide-Induced Non-Small Cell Lung Cancer Cell Proliferation, Migration, and Invasion by Suppressing the PI3K/AKT/MTOR and ERK/MAPK Signaling Pathways. *Thorac Cancer* **2019**, *10*, 1319–1333, doi:10.1111/1759-7714.13019.

72. Vuong, T.; Mallet, J.-F.; Ouzounova, M.; Rahbar, S.; Hernandez-Vargas, H.; Herceg, Z.; Matar, C. Role of a Polyphenol-Enriched Preparation on Chemoprevention of Mammary Carcinoma through Cancer Stem Cells and Inflammatory Pathways Modulation. *J Transl Med* **2016**, *14*, 13, doi:10.1186/s12967-016-0770-7.

73. Zhang, X.; Zhu, J.; Yan, J.; Xiao, Y.; Yang, R.; Huang, R.; Zhou, J.; Wang, Z.; Xiao, W.; Zheng, C.; et al. Systems Pharmacology Unravels the Synergic Target Space and Therapeutic Potential of Rhodiola Rosea L. for Non-Small Cell Lung Cancer. *Phytomedicine* **2020**, *79*, 153326, doi:10.1016/j.phymed.2020.153326.

74. Bokhari, R.A.; Tantowi, N.A.C.A.; Lau, S.F.; Mohamed, S. Java Tea (Orthosiphon Stamineus) Protected against Osteoarthritis by Mitigating Inflammation and Cartilage Degradation: A Preclinical Study. *Inflammopharmacology* **2018**, *26*, 939–949, doi:10.1007/s10787-017-0432-2.

75. Kim, B.; Park, B. Baohuoside I Suppresses Invasion of Cervical and Breast Cancer Cells through the Downregulation of CXCR4 Chemokine Receptor Expression. *Biochemistry* **2014**, *53*, 7562–7569, doi:10.1021/bi5011927.

76. Li, C.; Li, J.; Gong, S.; Huang, M.; Li, R.; Xiong, G.; Wang, F.; Zou, Q.; Qi, Q.; Yin, X. Targeting the ILK/YAP Axis by LFG-500 Blocks Epithelial–Mesenchymal Transition and Metastasis. *Acta Pharmacol Sin* **2021**, 1–13, doi:10.1038/s41401-021-00655-y, in press.

77. Shukla, K.; Sonowal, H.; Saxena, A.; Ramana, K.V. Didymin by Suppressing NF-ΚB Activation Prevents VEGF-Induced Angiogenesis in Vitro and in Vivo. *Vascul Pharmacol* **2019**, *115*, 18–25, doi:10.1016/j.vph.2019.01.002.

78. Xu, P.; Yan, F.; Zhao, Y.; Chen, X.; Sun, S.; Wang, Y.; Ying, L. Green Tea Polyphenol EGCG Attenuates MDSCs-Mediated Immunosuppression through Canonical and Non-Canonical Pathways in a 4T1 Murine Breast Cancer Model. *Nutrients* **2020**, *12*, doi:10.3390/nu12041042.

79. Qin, S.-K.; Li, Q.; Xu, J.M.; Liang, J.; Cheng, Y.; Fan, Y.; Jiang, J.; Ye, H.; Tao, H.; Li, L.; et al. Icaritin-Induced Immunomodulatory Efficacy in Advanced Hepatitis B Virus-Related Hepatocellular Carcinoma: Immunodynamic Biomarkers and Overall Survival. *Cancer Science* **2020**, *111*, 4218–4231, doi:10.1111/cas.14641.

80. Kashyap, D.; Garg, V.K.; Tuli, H.S.; Yerer, M.B.; Sak, K.; Sharma, A.K.; Kumar, M.; Aggarwal, V.; Sandhu, S.S. Fisetin and Quercetin: Promising Flavonoids with Chemopreventive Potential. *Biomolecules* **2019**, *9*, doi:10.3390/biom9050174.

81. Colapietro, A.; Mancini, A.; D’Alessandro, A.M.; Festuccia, C. Crocetin and Crocin from Saffron in Cancer Chemotherapy and Chemoprevention. *Anticancer Agents Med Chem* **2019**, *19*, 38–47, doi:10.2174/1871520619666181231112453.

82. Delmas, D.; Xiao, J.; Vejux, A.; Aires, V. Silymarin and Cancer: A Dual Strategy in Both in Chemoprevention and Chemosensitivity. *Molecules* **2020**, *25*, doi:10.3390/molecules25092009.

83. George, V.C.; Dellaire, G.; Rupasinghe, H.P.V. Plant Flavonoids in Cancer Chemoprevention: Role in Genome Stability. *J Nutr Biochem* **2017**, *45*, 1–14, doi:10.1016/j.jnutbio.2016.11.007.

84. Kim, D.H.; Khan, H.; Ullah, H.; Hassan, S.T.S.; Šmejkal, K.; Efferth, T.; Mahomoodally, M.F.; Xu, S.; Habtemariam, S.; Filosa, R.; et al. MicroRNA Targeting by Quercetin in Cancer Treatment and Chemoprotection. *Pharmacol Res* **2019**, *147*, 104346, doi:10.1016/j.phrs.2019.104346.

85. Clere, N.; Faure, S.; Martinez, M.C.; Andriantsitohaina, R. Anticancer Properties of Flavonoids: Roles in Various Stages of Carcinogenesis. *Cardiovasc Hematol Agents Med Chem* **2011**, *9*, 62–77, doi:10.2174/187152511796196498.

86. Murata, M. Inflammation and Cancer. *Environ Health Prev Med* **2018**, *23*, 50, doi:10.1186/s12199-018-0740-1.

87. Ramos-Nino, M.E. The Role of Chronic Inflammation in Obesity-Associated Cancers. *ISRN Oncology* **2013**, *2013*, e697521, doi:10.1155/2013/697521.

88. Dunn, B.K.; Umar, A.; Richmond, E. Introduction: Cancer Chemoprevention and Its Context. *Seminars in Oncology* **2016**, *43*, 19–21, doi:10.1053/j.seminoncol.2015.11.002.

89. García-Lafuente, A.; Guillamón, E.; Villares, A.; Rostagno, M.A.; Martínez, J.A. Flavonoids as Anti-Inflammatory Agents: Implications in Cancer and Cardiovascular Disease. *Inflamm. Res.* **2009**, *58*, 537–552, doi:10.1007/s00011-009-0037-3.

90. Tao, Y.; Zhan, S.; Wang, Y.; Zhou, G.; Liang, H.; Chen, X.; Shen, H. Baicalin, the Major Component of Traditional Chinese Medicine Scutellaria Baicalensis Induces Colon Cancer Cell Apoptosis through Inhibition of OncomiRNAs. *Scientific Reports* **2018**, *8*, 14477, doi:10.1038/s41598-018-32734-2.

91. Zhou, T.; Zhang, A.; Kuang, G.; Gong, X.; Jiang, R.; Lin, D.; Li, J.; Li, H.; Zhang, X.; Wan, J.; et al. Baicalin Inhibits the Metastasis of Highly Aggressive Breast Cancer Cells by Reversing Epithelial-to-Mesenchymal Transition by Targeting β-Catenin Signaling. *Oncol Rep* **2017**, *38*, 3599–3607, doi:10.3892/or.2017.6011.

92. Halliday, G.M.; Byrne, S.N.; Damian, D.L. Ultraviolet A Radiation: Its Role in Immunosuppression and Carcinogenesis. *Semin Cutan Med Surg* **2011**, *30*, 214–221, doi:10.1016/j.sder.2011.08.002.

93. Sherwani, M.A.; Yang, K.; Jani, A.; Abed, R.A.; Taufique, A.K.; Dosunmu, T.G.; Yusuf, N. Protective Effect of Baicalin Against TLR4-Mediated UVA-Induced Skin Inflammation. *Photochemistry and Photobiology* **2019**, *95*, 605–611, doi:https://doi.org/10.1111/php.13021.

94. Wang, C.-Z.; Zhang, C.-F.; Luo, Y.; Yao, H.; Yu, C.; Chen, L.; Yuan, J.; Huang, W.-H.; Wan, J.-Y.; Zeng, J.; et al. Baicalein, an Enteric Microbial Metabolite, Suppresses Gut Inflammation and Cancer Progression in ApcMin/+ Mice. *Clin Transl Oncol* **2020**, *22*, 1013–1022, doi:10.1007/s12094-019-02225-5.

95. McDowell, C.; Farooq, U.; Haseeb, M. Inflammatory Bowel Disease. In *StatPearls*; Publisher: StatPearls Publishing: Treasure Island (FL), USA, 2021.

96. Axelrad, J.E.; Lichtiger, S.; Yajnik, V. Inflammatory Bowel Disease and Cancer: The Role of Inflammation, Immunosuppression, and Cancer Treatment. *World J Gastroenterol* **2016**, *22*, 4794–4801, doi:10.3748/wjg.v22.i20.4794.

97. Habtemariam, S. Rutin as a Natural Therapy for Alzheimer’s Disease: Insights into Its Mechanisms of Action. *Curr Med Chem* **2016**, *23*, 860–873, doi:10.2174/0929867323666160217124333.

98. Kwon, K.H.; Murakami, A.; Tanaka, T.; Ohigashi, H. Dietary Rutin, but Not Its Aglycone Quercetin, Ameliorates Dextran Sulfate Sodium-Induced Experimental Colitis in Mice: Attenuation of pro-Inflammatory Gene Expression. *Biochem Pharmacol* **2005**, *69*, 395–406, doi:10.1016/j.bcp.2004.10.015.

99. Zhang, M.-J.; Su, H.; Yan, J.-Y.; Li, N.; Song, Z.-Y.; Wang, H.-J.; Huo, L.-G.; Wang, F.; Ji, W.-S.; Qu, X.-J.; et al. Chemopreventive Effect of Myricetin, a Natural Occurring Compound, on Colonic Chronic Inflammation and Inflammation-Driven Tumorigenesis in Mice. *Biomedicine & Pharmacotherapy* **2018**, *97*, 1131–1137, doi:10.1016/j.biopha.2017.11.018.

100. Zhang, Y.-S.; Wang, F.; Cui, S.-X.; Qu, X.-J. Natural Dietary Compound Naringin Prevents Azoxymethane/Dextran Sodium Sulfate-Induced Chronic Colorectal Inflammation and Carcinogenesis in Mice. *Cancer Biol Ther* **2018**, *19*, 735–744, doi:10.1080/15384047.2018.1453971.

101. Vega-Millán, C.B.; Dévora-Figueroa, A.G.; Burgess, J.L.; Beamer, P.I.; Furlong, M.; Lantz, R.C.; Meza-Figueroa, D.; O´Rourke, M.K.; García-Rico, L.; Meza-Escalante, E.R.; et al. Inflammation Biomarkers Associated with Arsenic Exposure by Drinking Water and Respiratory Outcomes in Indigenous Children from Three Yaqui Villages in Southern Sonora, México. *Environ Sci Pollut Res* **2021**, doi:10.1007/s11356-021-13070-x.

102. Riegsecker, S.; Wiczynski, D.; Kaplan, M.J.; Ahmed, S. Potential Benefits of Green Tea Polyphenol EGCG in the Prevention and Treatment of Vascular Inflammation in Rheumatoid Arthritis. *Life Sci* **2013**, *93*, 307–312, doi:10.1016/j.lfs.2013.07.006.

103. Yu, N.-H.; Pei, H.; Huang, Y.-P.; Li, Y.-F. (-)-Epigallocatechin-3-Gallate Inhibits Arsenic-Induced Inflammation and Apoptosis through Suppression of Oxidative Stress in Mice. *Cell Physiol Biochem* **2017**, *41*, 1788–1800, doi:10.1159/000471911.

104. Meeran, S.M.; Akhtar, S.; Katiyar, S.K. Inhibition of UVB-Induced Skin Tumor Development by Drinking Green Tea Polyphenols Is Mediated through DNA Repair and Subsequent Inhibition of Inflammation. *J Invest Dermatol* **2009**, *129*, 1258–1270, doi:10.1038/jid.2008.354.

105. Lamb, A.; Chen, L.-F. Role of the Helicobacter Pylori-Induced Inflammatory Response in the Development of Gastric Cancer. *J Cell Biochem* **2013**, *114*, 491–497, doi:10.1002/jcb.24389.

106. Zhang, X.-Y.; Zhang, P.-Y.; Aboul-Soud, M.A.M. From Inflammation to Gastric Cancer: Role of Helicobacter Pylori. *Oncol Lett* **2017**, *13*, 543–548, doi:10.3892/ol.2016.5506.

107. Siriviriyakul, P.; Werawatganon, D.; Phetnoo, N.; Somanawat, K.; Chatsuwan, T.; Klaikeaw, N.; Chayanupatkul, M. Genistein Attenuated Gastric Inflammation and Apoptosis in Helicobacter Pylori-Induced Gastropathy in Rats. *BMC Gastroenterology* **2020**, *20*, 410, doi:10.1186/s12876-020-01555-x.

108. Hajialyani, M.; Hosein Farzaei, M.; Echeverría, J.; Nabavi, S.M.; Uriarte, E.; Sobarzo-Sánchez, E. Hesperidin as a Neuroprotective Agent: A Review of Animal and Clinical Evidence. *Molecules* **2019**, *24*, doi:10.3390/molecules24030648.

109. Francescone, R.; Hou, V.; Grivennikov, S.I. Cytokines, IBD and Colitis-Associated Cancer. *Inflamm Bowel Dis* **2015**, *21*, 409–418, doi:10.1097/MIB.0000000000000236.

110. Guazelli, C.F.S.; Fattori, V.; Ferraz, C.R.; Borghi, S.M.; Casagrande, R.; Baracat, M.M.; Verri, W.A. Antioxidant and Anti-Inflammatory Effects of Hesperidin Methyl Chalcone in Experimental Ulcerative Colitis. *Chem Biol Interact* **2021**, *333*, 109315, doi:10.1016/j.cbi.2020.109315.

111. Comalada, M.; Camuesco, D.; Sierra, S.; Ballester, I.; Xaus, J.; Gálvez, J.; Zarzuelo, A. In Vivo Quercitrin Anti-Inflammatory Effect Involves Release of Quercetin, Which Inhibits Inflammation through down-Regulation of the NF-KappaB Pathway. *Eur J Immunol* **2005**, *35*, 584–592, doi:10.1002/eji.200425778.

112. Lin, Y.; Shi, R.; Wang, X.; Shen, H.-M. Luteolin, a Flavonoid with Potentials for Cancer Prevention and Therapy. *Curr Cancer Drug Targets* **2008**, *8*, 634–646.

113. Luo, Y.; Shang, P.; Li, D. Luteolin: A Flavonoid That Has Multiple Cardio-Protective Effects and Its Molecular Mechanisms. *Front Pharmacol* **2017**, *8*, 692, doi:10.3389/fphar.2017.00692.

114. Yao, Z.-H.; Yao, X.-L.; Zhang, Y.; Zhang, S.-F.; Hu, J.-C. Luteolin Could Improve Cognitive Dysfunction by Inhibiting Neuroinflammation. *Neurochem Res* **2018**, *43*, 806–820, doi:10.1007/s11064-018-2482-2.

115. Beaver, L.M.; Stemmy, E.J.; Schwartz, A.M.; Damsker, J.M.; Constant, S.L.; Ceryak, S.M.; Patierno, S.R. Lung Inflammation, Injury, and Proliferative Response after Repetitive Particulate Hexavalent Chromium Exposure. *Environ Health Perspect* **2009**, *117*, 1896–1902, doi:10.1289/ehp.0900715.

116. Pratheeshkumar, P.; Son, Y.-O.; Divya, S.P.; Roy, R.V.; Hitron, J.A.; Wang, L.; Kim, D.; Dai, J.; Asha, P.; Zhang, Z.; et al. Luteolin Inhibits Cr(VI)-Induced Malignant Cell Transformation of Human Lung Epithelial Cells by Targeting ROS Mediated Multiple Cell Signaling Pathways. *Toxicol Appl Pharmacol* **2014**, *281*, 230–241, doi:10.1016/j.taap.2014.10.008.

117. Pace, E.; Di Vincenzo, S.; Di Salvo, E.; Genovese, S.; Dino, P.; Sangiorgi, C.; Ferraro, M.; Gangemi, S. MiR-21 Upregulation Increases IL-8 Expression and Tumorigenesis Program in Airway Epithelial Cells Exposed to Cigarette Smoke. *J Cell Physiol* **2019**, *234*, 22183–22194, doi:10.1002/jcp.28786.

118. Katiyar, S.K.; Agarwal, R.; Mukhtar, H. Inhibition of Both Stage I and Stage II Skin Tumor Promotion in SENCAR Mice by a Polyphenolic Fraction Isolated from Green Tea: Inhibition Depends on the Duration of Polyphenol Treatment. *Carcinogenesis* **1993**, *14*, 2641–2643, doi:10.1093/carcin/14.12.2641.

119. Lahiri-Chatterjee, M.; Katiyar, S.K.; Mohan, R.R.; Agarwal, R. A Flavonoid Antioxidant, Silymarin, Affords Exceptionally High Protection against Tumor Promotion in the SENCAR Mouse Skin Tumorigenesis Model. *Cancer Res* **1999**, *59*, 622–632.

120. Wei, H.; Tye, L.; Bresnick, E.; Birt, D.F. Inhibitory Effect of Apigenin, a Plant Flavonoid, on Epidermal Ornithine Decarboxylase and Skin Tumor Promotion in Mice. *Cancer Res* **1990**, *50*, 499–502.

121. Granato, M.; Rizzello, C.; Gilardini Montani, M.S.; Cuomo, L.; Vitillo, M.; Santarelli, R.; Gonnella, R.; D’Orazi, G.; Faggioni, A.; Cirone, M. Quercetin Induces Apoptosis and Autophagy in Primary Effusion Lymphoma Cells by Inhibiting PI3K/AKT/MTOR and STAT3 Signaling Pathways. *J Nutr Biochem* **2017**, *41*, 124–136, doi:10.1016/j.jnutbio.2016.12.011.

122. Zhu, J. feng; Li, Z. jian; Zhang, G. sen; Meng, K.; Kuang, W. yong; Li, J.; Zhou, X. fu; Li, R. juan; Peng, H. ling; Dai, C. wen; et al. Icaritin Shows Potent Anti-Leukemia Activity on Chronic Myeloid Leukemia In Vitro and In Vivo by Regulating MAPK/ERK/JNK and JAK2/STAT3 /AKT Signalings. *PLoS One* **2011**, *6*, e23720, doi:10.1371/journal.pone.0023720.

123. Peng, H.-L.; Huang, W.-C.; Cheng, S.-C.; Liou, C.-J. Fisetin Inhibits the Generation of Inflammatory Mediators in Interleukin-1β-Induced Human Lung Epithelial Cells by Suppressing the NF-ΚB and ERK1/2 Pathways. *Int Immunopharmacol* **2018**, *60*, 202–210, doi:10.1016/j.intimp.2018.05.004.

124. Zhang, X.-J.; Jia, S.-S. Fisetin Inhibits Laryngeal Carcinoma through Regulation of AKT/NF-ΚB/MTOR and ERK1/2 Signaling Pathways. *Biomed Pharmacother* **2016**, *83*, 1164–1174, doi:10.1016/j.biopha.2016.08.035.

125. Shi, B.; Wang, L.-F.; Meng, W.-S.; Chen, L.; Meng, Z.-L. Carnosic Acid and Fisetin Combination Therapy Enhances Inhibition of Lung Cancer through Apoptosis Induction. *Int J Oncol* **2017**, *50*, 2123–2135, doi:10.3892/ijo.2017.3970.

126. You, Y.; Wang, R.; Shao, N.; Zhi, F.; Yang, Y. Luteolin Suppresses Tumor Proliferation through Inducing Apoptosis and Autophagy via MAPK Activation in Glioma. *Onco Targets Ther* **2019**, *12*, 2383–2396, doi:10.2147/OTT.S191158.

127. Song, S.; Su, Z.; Xu, H.; Niu, M.; Chen, X.; Min, H.; Zhang, B.; Sun, G.; Xie, S.; Wang, H.; et al. Luteolin Selectively Kills STAT3 Highly Activated Gastric Cancer Cells through Enhancing the Binding of STAT3 to SHP-1. *Cell Death Dis* **2017**, *8*, e2612, doi:10.1038/cddis.2017.38.

128. Jiang, Z.-B.; Wang, W.-J.; Xu, C.; Xie, Y.-J.; Wang, X.-R.; Zhang, Y.-Z.; Huang, J.-M.; Huang, M.; Xie, C.; Liu, P.; et al. Luteolin and Its Derivative Apigenin Suppress the Inducible PD-L1 Expression to Improve Anti-Tumor Immunity in KRAS-Mutant Lung Cancer. *Cancer Lett* **2021**, *515*, 36–48, doi:10.1016/j.canlet.2021.05.019.

129. Yang, J.; Pi, C.; Wang, G. Inhibition of PI3K/Akt/MTOR Pathway by Apigenin Induces Apoptosis and Autophagy in Hepatocellular Carcinoma Cells. *Biomed Pharmacother* **2018**, *103*, 699–707, doi:10.1016/j.biopha.2018.04.072.

130. Park, C.-H.; Min, S.-Y.; Yu, H.-W.; Kim, K.; Kim, S.; Lee, H.-J.; Kim, J.-H.; Park, Y.-J. Effects of Apigenin on RBL-2H3, RAW264.7, and HaCaT Cells: Anti-Allergic, Anti-Inflammatory, and Skin-Protective Activities. *Int J Mol Sci* **2020**, *21*, E4620, doi:10.3390/ijms21134620.

131. Qiu, J.-G.; Wang, L.; Liu, W.-J.; Wang, J.-F.; Zhao, E.-J.; Zhou, F.-M.; Ji, X.-B.; Wang, L.-H.; Xia, Z.-K.; Wang, W.; et al. Apigenin Inhibits IL-6 Transcription and Suppresses Esophageal Carcinogenesis. *Front Pharmacol* **2019**, *10*, 1002, doi:10.3389/fphar.2019.01002.

132. Hara, S.; Morita, R.; Ogawa, T.; Segawa, R.; Takimoto, N.; Suzuki, K.; Hamadate, N.; Hayashi, S.-M.; Odachi, A.; Ogiwara, I.; et al. Tumor Suppression Effects of Bilberry Extracts and Enzymatically Modified Isoquercitrin in Early Preneoplastic Liver Cell Lesions Induced by Piperonyl Butoxide Promotion in a Two-Stage Rat Hepatocarcinogenesis Model. *Exp Toxicol Pathol* **2014**, *66*, 225–234, doi:10.1016/j.etp.2014.02.002.

133. Chen, L.; Guo, D. The Functions of Tumor Suppressor PTEN in Innate and Adaptive Immunity. *Cell Mol Immunol* **2017**, *14*, 581–589, doi:10.1038/cmi.2017.30.

134. Zhao, Z.; Liu, B.; Sun, J.; Lu, L.; Liu, L.; Qiu, J.; Li, Q.; Yan, C.; Jiang, S.; Mohammadtursun, N.; et al. Scutellaria Flavonoids Effectively Inhibit the Malignant Phenotypes of Non-Small Cell Lung Cancer in an Id1-Dependent Manner. *Int J Biol Sci* **2019**, *15*, 1500–1513, doi:10.7150/ijbs.33146.

135. Ke, M.; Zhang, Z.; Xu, B.; Zhao, S.; Ding, Y.; Wu, X.; Wu, R.; Lv, Y.; Dong, J. Baicalein and Baicalin Promote Antitumor Immunity by Suppressing PD-L1 Expression in Hepatocellular Carcinoma Cells. *Int Immunopharmacol* **2019**, *75*, 105824, doi:10.1016/j.intimp.2019.105824.

136. Bucio-Noble, D.; Kautto, L.; Krisp, C.; Ball, M.S.; Molloy, M.P. Polyphenol Extracts from Dried Sugarcane Inhibit Inflammatory Mediators in an in Vitro Colon Cancer Model. *J Proteomics* **2018**, *177*, 1–10, doi:10.1016/j.jprot.2018.02.009.

137. Basak, S.K.; Bera, A.; Yoon, A.J.; Morselli, M.; Jeong, C.; Tosevska, A.; Dong, T.S.; Eklund, M.; Russ, E.; Nasser, H.; et al. A Randomized, Phase 1, Placebo-Controlled Trial of APG-157 in Oral Cancer Demonstrates Systemic Absorption and an Inhibitory Effect on Cytokines and Tumor-Associated Microbes. *Cancer* **2020**, *126*, 1668–1682, doi:10.1002/cncr.32644.

138. Farsad-Naeimi, A.; Alizadeh, M.; Esfahani, A.; Darvish Aminabad, E. Effect of Fisetin Supplementation on Inflammatory Factors and Matrix Metalloproteinase Enzymes in Colorectal Cancer Patients. *Food Funct* **2018**, *9*, 2025–2031, doi:10.1039/c7fo01898c.

139. Henning, S.M.; Wang, P.; Said, J.W.; Huang, M.; Grogan, T.; Elashoff, D.; Carpenter, C.L.; Heber, D.; Aronson, W.J. Randomized Clinical Trial of Brewed Green and Black Tea in Men with Prostate Cancer Prior to Prostatectomy. *Prostate* **2015**, *75*, 550–559, doi:10.1002/pros.22943.

140. Lesinski, G.B.; Reville, P.K.; Mace, T.A.; Young, G.S.; Ahn-Jarvis, J.; Thomas-Ahner, J.; Vodovotz, Y.; Ameen, Z.; Grainger, E.; Riedl, K.; et al. Consumption of Soy Isoflavone Enriched Bread in Men with Prostate Cancer Is Associated with Reduced Proinflammatory Cytokines and Immunosuppressive Cells. *Cancer Prev Res (Phila)* **2015**, *8*, 1036–1044, doi:10.1158/1940-6207.CAPR-14-0464.

141. Shike, M.; Doane, A.S.; Russo, L.; Cabal, R.; Reis-Filho, J.S.; Gerald, W.; Cody, H.; Khanin, R.; Bromberg, J.; Norton, L. The Effects of Soy Supplementation on Gene Expression in Breast Cancer: A Randomized Placebo-Controlled Study. *J Natl Cancer Inst* **2014**, *106*, dju189, doi:10.1093/jnci/dju189.

142. Dong, W.; Chen, A.; Chao, X.; Li, X.; Cui, Y.; Xu, C.; Cao, J.; Ning, Y. Chrysin Inhibits Proinflammatory Factor-Induced EMT Phenotype and Cancer Stem Cell-Like Features in HeLa Cells by Blocking the NF-ΚB/Twist Axis. *Cell Physiol Biochem* **2019**, *52*, 1236–1250, doi:10.33594/000000084.

143. Yan, Y.; Liu, X.; Gao, J.; Wu, Y.; Li, Y. Inhibition of TGF-β Signaling in Gliomas by the Flavonoid Diosmetin Isolated from Dracocephalum Peregrinum L. *Molecules* **2020**, *25*, 192, doi:10.3390/molecules25010192.

144. Wang, Y.; Li, J.-J.; Chen, Y.-M. Biochanin A Extirpates the Epithelial-Mesenchymal Transition in a Human Lung Cancer. *Exp Ther Med* **2018**, *15*, 2830–2836, doi:10.3892/etm.2018.5731.

145. Li, J.; Wang, T.; Liu, P.; Yang, F.; Wang, X.; Zheng, W.; Sun, W. Hesperetin Ameliorates Hepatic Oxidative Stress and Inflammation via the PI3K/AKT-Nrf2-ARE Pathway in Oleic Acid-Induced HepG2 Cells and a Rat Model of High-Fat Diet-Induced NAFLD. *Food Funct* **2021**, *12*, 3898–3918, doi:10.1039/d0fo02736g.

146. Wei, R.; Cortez Penso, N.E.; Hackman, R.M.; Wang, Y.; Mackenzie, G.G. Epigallocatechin-3-Gallate (EGCG) Suppresses Pancreatic Cancer Cell Growth, Invasion, and Migration Partly through the Inhibition of Akt Pathway and Epithelial–Mesenchymal Transition: Enhanced Efficacy When Combined with Gemcitabine. *Nutrients* **2019**, *11*, 1856, doi:10.3390/nu11081856.

147. Kang, H.R.; Moon, J.Y.; Ediriweera, M.K.; Song, Y.W.; Cho, M.; Kasiviswanathan, D.; Cho, S.K. Dietary Flavonoid Myricetin Inhibits Invasion and Migration of Radioresistant Lung Cancer Cells (A549‐IR) by Suppressing MMP‐2 and MMP‐9 Expressions through Inhibition of the FAK‐ERK Signaling Pathway. *Food Sci Nutr* **2020**, *8*, 2059–2067, doi:10.1002/fsn3.1495.

148. Tuponchai, P.; Kukongviriyapan, V.; Prawan, A.; Kongpetch, S.; Senggunprai, L. Myricetin Ameliorates Cytokine-Induced Migration and Invasion of Cholangiocarcinoma Cells via Suppression of STAT3 Pathway. *J Cancer Res Ther* **2019**, *15*, 157–163, doi:10.4103/jcrt.JCRT_287_17.

149. Gu, Y.; Yu, J.; Ding, C.; Zhou, Y.; Yang, J.; Yu, W.; Zhang, X.; Huang, H. Flavonoid GL-V9 Suppresses Invasion and Migration of Human Colorectal Cancer Cells by Inhibiting PI3K/Akt and MMP-2/9 Signaling. *J Cancer* **2021**, *12*, 4542–4551, doi:10.7150/jca.58710.

150. Susmitha, G.D.; Miyazato, K.; Ogura, K.; Yokoyama, S.; Hayakawa, Y. Anti-Metastatic Effects of Baicalein by Targeting STAT3 Activity in Breast Cancer Cells. *Biol Pharm Bull* **2020**, *43*, 1899–1905, doi:10.1248/bpb.b20-00571.

151. Salama, A.A.A.; Allam, R.M. Promising Targets of Chrysin and Daidzein in Colorectal Cancer: Amphiregulin, CXCL1, and MMP-9. *Eur J Pharmacol* **2021**, *892*, 173763, doi:10.1016/j.ejphar.2020.173763.

152. Ko, Y.-C.; Choi, H.S.; Liu, R.; Kim, J.-H.; Kim, S.-L.; Yun, B.-S.; Lee, D.-S. Inhibitory Effects of Tangeretin, a Citrus Peel-Derived Flavonoid, on Breast Cancer Stem Cell Formation through Suppression of Stat3 Signaling. *Molecules* **2020**, *25*, 2599, doi:10.3390/molecules25112599.

153. Sun, S.; Cui, Y.; Ren, K.; Quan, M.; Song, Z.; Zou, H.; Li, D.; Zheng, Y.; Cao, J. 8-Bromo-7-Methoxychrysin Reversed M2 Polarization of Tumor-Associated Macrophages Induced by Liver Cancer Stem-like Cells. *Anticancer Agents Med Chem* **2017**, *17*, 286–293, doi:10.2174/1871520616666160204112556.

154. Gong, G.; Wang, H.; Kong, X.; Duan, R.; Dong, T.T.X.; Tsim, K.W.K. Flavonoids Are Identified from the Extract of Scutellariae Radix to Suppress Inflammatory-Induced Angiogenic Responses in Cultured RAW 264.7 Macrophages. *Sci Rep* **2018**, *8*, 17412, doi:10.1038/s41598-018-35817-2.

155. Li, C.; Wang, Q.; Shen, S.; Wei, X.; Li, G. HIF-1α/VEGF Signaling-Mediated Epithelial-Mesenchymal Transition and Angiogenesis Is Critically Involved in Anti-Metastasis Effect of Luteolin in Melanoma Cells. *Phytother Res* **2019**, *33*, 798–807, doi:10.1002/ptr.6273.

156. Mirzaaghaei, S.; Foroughmand, A.M.; Saki, G.; Shafiei, M. Combination of Epigallocatechin-3-Gallate and Silibinin: A Novel Approach for Targeting Both Tumor and Endothelial Cells. *ACS Omega* **2019**, *4*, 8421–8430, doi:10.1021/acsomega.9b00224.

157. Tsai, C.-H.; Tzeng, S.-F.; Hsieh, S.-C.; Yang, Y.-C.; Hsiao, Y.-W.; Tsai, M.-H.; Hsiao, P.-W. A Standardized Herbal Extract Mitigates Tumor Inflammation and Augments Chemotherapy Effect of Docetaxel in Prostate Cancer. *Sci Rep* **2017**, *7*, 15624, doi:10.1038/s41598-017-15934-0.

158. Ishak, N.I.M.; Mohamed, S.; Madzuki, I.N.; Mustapha, N.M.; Esa, N.M. Limonin Modulated Immune and Inflammatory Responses to Suppress Colorectal Adenocarcinoma in Mice Model. *Naunyn Schmiedebergs Arch Pharmacol* **2021**, doi:10.1007/s00210-021-02101-6.

159. McLarty, J.; Bigelow, R.L.H.; Smith, M.; Elmajian, D.; Ankem, M.; Cardelli, J.A. Tea Polyphenols Decrease Serum Levels of Prostate-Specific Antigen, Hepatocyte Growth Factor, and Vascular Endothelial Growth Factor in Prostate Cancer Patients and Inhibit Production of Hepatocyte Growth Factor and Vascular Endothelial Growth Factor in Vitro. *Cancer Prev Res (Phila)* **2009**, *2*, 673–682, doi:10.1158/1940-6207.CAPR-08-0167.

160. Sturgeon, K.M.; Foo, W.; Heroux, M.; Schmitz, K. Change in Inflammatory Biomarkers and Adipose Tissue in BRCA1/2 Breast Cancer Survivors Following a Yearlong Lifestyle Modification Program. *Cancer Prev Res* **2018**, *11*, 545–550.

161. Qian, S.; Golubnitschaja, O.; Zhan, X. Chronic Inflammation: Key Player and Biomarker-Set to Predict and Prevent Cancer Development and Progression Based on Individualized Patient Profiles. *EPMA J* **2019**, *10*, 365–381, doi:10.1007/s13167-019-00194-x.

162. U.S. Department of Agriculture, Agricultural Research Service. USDA National Nutrient Database for the Flavonoid Content of Selected Foods, Release 3.0. 2011. [(Accessed on 2 June 2013)]. Available Online: . Available online: http://www.ars.usda.gov/SP2UserFiles/Place/12354500/Data/Flav/Flav_R03.pdf (accessed on 5 June 2020).

163. Showing All Foods in Which the Polyphenol Myricetin Is Found - Phenol-Explorer Available online: http://phenol-explorer.eu/contents/polyphenol/309 (accessed on 9 September 2021).

164. Showing All Foods in Which the Polyphenol Biochanin A Is Found - Phenol-Explorer Available online: http://phenol-explorer.eu/contents/polyphenol/397 (accessed on 9 September 2021).

165. Showing Details for Content Value of Baicalein in Welsh Onion, Fresh - Phenol-Explorer Available online: http://phenol-explorer.eu/contents/show/2/266/477 (accessed on 9 September 2021).

166. U.S. Department of Agriculture, Agricultural Research Service. USDA National Nutrient Database for the Isoflavone Content of Selected Foods, Release 2.0. 2008. [(Accessed on 2 June 2013)]. Available Online: Available online: http://www.ars.usda.gov/SP2UserFiles/Place/12354500/Data/isoflav/Isoflav_R2.pdf. (accessed on 5 June 2020).

167. Showing All Foods in Which the Polyphenol Tangeretin Is Found - Phenol-Explorer Available online: http://phenol-explorer.eu/contents/polyphenol/238?fbclid=IwAR130SQvsBI-XtgGjasfPVGpmFb60_MqoJVyzVMbqR07vFQIoxTQwAwmIhk (accessed on 9 September 2021).

168. Showing All Foods in Which the Polyphenol Genistin Is Found - Phenol-Explorer Available online: http://phenol-explorer.eu/contents/polyphenol/404 (accessed on 9 September 2021).

169. Showing All Foods in Which the Polyphenol Diosmin Is Found - Phenol-Explorer Available online: http://phenol-explorer.eu/contents/polyphenol/232 (accessed on 9 September 2021).

170. Showing All Foods in Which the Polyphenol Luteolin Is Found - Phenol-Explorer Available online: http://phenol-explorer.eu/contents/polyphenol/229 (accessed on 9 September 2021).

171. Kucera, R.; Pecen, L.; Topolcan, O.; Dahal, A.R.; Costigliola, V.; Giordano, F.A.; Golubnitschaja, O. Prostate Cancer Management: Long-Term Beliefs, Epidemic Developments in the Early Twenty-First Century and 3PM Dimensional Solutions. *EPMA Journal* **2020**, doi:10.1007/s13167-020-00214-1.

172. Golubnitschaja, O.; Liskova, A.; Koklesova, L.; Samec, M.; Biringer, K.; Büsselberg, D.; Podbielska, H.; Kunin, A.A.; Evsevyeva, M.E.; Shapira, N.; et al. Caution, “Normal” BMI: Health Risks Associated with Potentially Masked Individual Underweight-EPMA Position Paper 2021. *EPMA J* **2021**, 1–22, doi:10.1007/s13167-021-00251-4.

173. Wang, W.; Yan, Y.; Guo, Z.; Hou, H.; Garcia, M.; Tan, X.; Anto, E.; et al. ALL AROUND SUBOPTIMAL HEALTH. A Joint Position Paper of the Suboptimal Health Study Consortium and European Association for Predictive, Preventive and Personalised Medicine. *EMPA J.* **2021**, accepted.

174. Torres Crigna, A.; Link, B.; Samec, M.; Giordano, F.A.; Kubatka, P.; Golubnitschaja, O. Endothelin-1 Axes in the Framework of Predictive, Preventive and Personalised (3P) Medicine. *EPMA J* **2021**, 1–41, doi:10.1007/s13167-021-00248-z.

175. *Flammer Syndrome: From Phenotype to Associated Pathologies, Prediction, Prevention and Personalisation*; Golubnitschaja, O., Ed.; Advances in Predictive, Preventive and Personalised Medicine; Springer International Publishing: Cham, 2019; Vol. 11; ISBN 978-3-030-13549-2.

176. Marranzano, M.; Ray, S.; Godos, J.; Galvano, F. Association between Dietary Flavonoids Intake and Obesity in a Cohort of Adults Living in the Mediterranean Area. *Int J Food Sci Nutr* **2018**, *69*, 1020–1029, doi:10.1080/09637486.2018.1452900.

177. Kapinova, A.; Stefanicka, P.; Kubatka, P.; Zubor, P.; Uramova, S.; Kello, M.; Mojzis, J.; Blahutova, D.; Qaradakhi, T.; Zulli, A.; et al. Are Plant-Based Functional Foods Better Choice against Cancer than Single Phytochemicals? A Critical Review of Current Breast Cancer Research. *Biomed Pharmacother* **2017**, *96*, 1465–1477, doi:10.1016/j.biopha.2017.11.134.

178. Akhlaghi, M.; Ghobadi, S.; Mohammad Hosseini, M.; Gholami, Z.; Mohammadian, F. Flavanols Are Potential Anti-Obesity Agents, a Systematic Review and Meta-Analysis of Controlled Clinical Trials. *Nutr Metab Cardiovasc Dis* **2018**, *28*, 675–690, doi:10.1016/j.numecd.2018.04.001.

179. García-Barrado, M.J.; Iglesias-Osma, M.C.; Pérez-García, E.; Carrero, S.; Blanco, E.J.; Carretero-Hernández, M.; Carretero, J. Role of Flavonoids in the Interactions among Obesity, Inflammation, and Autophagy. *Pharmaceuticals (Basel)* **2020**, *13*, 342, doi:10.3390/ph13110342.

180. Gentile, D.; Fornai, M.; Colucci, R.; Pellegrini, C.; Tirotta, E.; Benvenuti, L.; Segnani, C.; Ippolito, C.; Duranti, E.; Virdis, A.; et al. The Flavonoid Compound Apigenin Prevents Colonic Inflammation and Motor Dysfunctions Associated with High Fat Diet-Induced Obesity. *PLoS One* **2018**, *13*, e0195502, doi:10.1371/journal.pone.0195502.

181. Baek, Y.; Lee, M.N.; Wu, D.; Pae, M. Luteolin Reduces Adipose Tissue Macrophage Inflammation and Insulin Resistance in Postmenopausal Obese Mice. *J Nutr Biochem* **2019**, *71*, 72–81, doi:10.1016/j.jnutbio.2019.06.002.

182. Lee, M.; Sorn, S.R.; Park, Y.; Park, H.-K. Anthocyanin Rich-Black Soybean Testa Improved Visceral Fat and Plasma Lipid Profiles in Overweight/Obese Korean Adults: A Randomized Controlled Trial. *J Med Food* **2016**, *19*, 995–1003, doi:10.1089/jmf.2016.3762.

183. Li, D.; Zhang, Y.; Liu, Y.; Sun, R.; Xia, M. Purified Anthocyanin Supplementation Reduces Dyslipidemia, Enhances Antioxidant Capacity, and Prevents Insulin Resistance in Diabetic Patients. *J Nutr* **2015**, *145*, 742–748, doi:10.3945/jn.114.205674.

184. Arcidiacono, B.; Iiritano, S.; Nocera, A.; Possidente, K.; Nevolo, M.T.; Ventura, V.; Foti, D.; Chiefari, E.; Brunetti, A. Insulin Resistance and Cancer Risk: An Overview of the Pathogenetic Mechanisms. *Exp Diabetes Res* **2012**, *2012*, 789174, doi:10.1155/2012/789174.

185. Spencer, J.P.E. Flavonoids and Brain Health: Multiple Effects Underpinned by Common Mechanisms. *Genes Nutr* **2009**, *4*, 243–250, doi:10.1007/s12263-009-0136-3.

186. Wang, J.; Cheng, C.; Xin, C.; Wang, Z. The Antidepressant-like Effect of Flavonoids from Trigonella Foenum-Graecum Seeds in Chronic Restraint Stress Mice via Modulation of Monoamine Regulatory Pathways. *Molecules* **2019**, *24*, 1105, doi:10.3390/molecules24061105.

187. Sarriá, B.; Martínez-López, S.; Sierra-Cinos, J.L.; García-Diz, L.; Mateos, R.; Bravo, L. Regular Consumption of a Cocoa Product Improves the Cardiometabolic Profile in Healthy and Moderately Hypercholesterolaemic Adults. *Br J Nutr* **2014**, *111*, 122–134, doi:10.1017/S000711451300202X.

188. Mellor, D.D.; Madden, L.A.; Smith, K.A.; Kilpatrick, E.S.; Atkin, S.L. High-Polyphenol Chocolate Reduces Endothelial Dysfunction and Oxidative Stress during Acute Transient Hyperglycaemia in Type 2 Diabetes: A Pilot Randomized Controlled Trial. *Diabet Med* **2013**, *30*, 478–483, doi:10.1111/dme.12030.

189. Ciumărnean, L.; Milaciu, M.V.; Runcan, O.; Vesa, Ștefan C.; Răchișan, A.L.; Negrean, V.; Perné, M.-G.; Donca, V.I.; Alexescu, T.-G.; Para, I.; et al. The Effects of Flavonoids in Cardiovascular Diseases. *Molecules* **2020**, *25*, 4320, doi:10.3390/molecules25184320.

190. Mastantuono, T.; Battiloro, L.; Sabatino, L.; Chiurazzi, M.; Di Maro, M.; Muscariello, E.; Colantuoni, A.; Lapi, D. Effects of Citrus Flavonoids Against Microvascular Damage Induced by Hypoperfusion and Reperfusion in Rat Pial Circulation. *Microcirculation* **2015**, *22*, 378–390, doi:10.1111/micc.12207.

191. de Albuquerque, R.D.D.G.; Perini, J.A.; Machado, D.E.; Angeli-Gamba, T.; Esteves, R. dos S.; Santos, M.G.; Oliveira, A.P.; Rocha, L. Wound Healing Activity and Chemical Standardization of Eugenia Pruniformis Cambess. *Pharmacogn Mag* **2016**, *12*, 288–294, doi:10.4103/0973-1296.192206.

192. Antunes-Ricardo, M.; Gutiérrez-Uribe, J.; Serna-Saldívar, S.O. Anti-Inflammatory Glycosylated Flavonoids as Therapeutic Agents for Treatment of Diabetes-Impaired Wounds. *Curr Top Med Chem* **2015**, *15*, 2456–2463, doi:10.2174/1568026615666150619141702.

193. Olas, B. Honey and Its Phenolic Compounds as an Effective Natural Medicine for Cardiovascular Diseases in Humans? *Nutrients* **2020**, *12*, 283, doi:10.3390/nu12020283.

194. Efem, S.E. Clinical Observations on the Wound Healing Properties of Honey. *Br J Surg* **1988**, *75*, 679–681, doi:10.1002/bjs.1800750718.

195. Henriques, A.; Jackson, S.; Cooper, R.; Burton, N. Free Radical Production and Quenching in Honeys with Wound Healing Potential. *J Antimicrob Chemother* **2006**, *58*, 773–777, doi:10.1093/jac/dkl336.

196. Atrahimovich, D.; Avni, D.; Khatib, S. Flavonoids-Macromolecules Interactions in Human Diseases with Focus on Alzheimer, Atherosclerosis and Cancer. *Antioxidants (Basel)* **2021**, *10*, 423, doi:10.3390/antiox10030423.

197. Ginwala, R.; Bhavsar, R.; Chigbu, D.I.; Jain, P.; Khan, Z.K. Potential Role of Flavonoids in Treating Chronic Inflammatory Diseases with a Special Focus on the Anti-Inflammatory Activity of Apigenin. *Antioxidants (Basel)* **2019**, *8*, E35, doi:10.3390/antiox8020035.

198. Rodríguez-García, C.; Sánchez-Quesada, C.; Gaforio, J.J. Dietary Flavonoids as Cancer Chemopreventive Agents: An Updated Review of Human Studies. *Antioxidants (Basel)* **2019**, *8*, doi:10.3390/antiox8050137.

199. Jenkins, D.J.A.; Kendall, C.W.C.; Connelly, P.W.; Jackson, C.-J.C.; Parker, T.; Faulkner, D.; Vidgen, E. Effects of High- and Low-Isoflavone (Phytoestrogen) Soy Foods on Inflammatory Biomarkers and Proinflammatory Cytokines in Middle-Aged Men and Women. *Metabolism* **2002**, *51*, 919–924, doi:10.1053/meta.2002.33352.

200. Toledo, E.; Salas-Salvadó, J.; Donat-Vargas, C.; Buil-Cosiales, P.; Estruch, R.; Ros, E.; Corella, D.; Fitó, M.; Hu, F.B.; Arós, F.; et al. Mediterranean Diet and Invasive Breast Cancer Risk Among Women at High Cardiovascular Risk in the PREDIMED Trial: A Randomized Clinical Trial. *JAMA Intern Med* **2015**, *175*, 1752–1760, doi:10.1001/jamainternmed.2015.4838.

201. Castelló, A.; Pollán, M.; Buijsse, B.; Ruiz, A.; Casas, A.M.; Baena-Cañada, J.M.; Lope, V.; Antolýn, S.; Ramos, M.; Muñoz, M.; et al. Spanish Mediterranean Diet and Other Dietary Patterns and Breast Cancer Risk: Case-Control EpiGEICAM Study. *British Journal of Cancer* **2014**, *111*, 1454–1462, doi:10.1038/bjc.2014.434.

202. Gerner, C.; Costigliola, V.; Golubnitschaja, O. Multiomic Patterns in Body Fluids: Technological Challenge with a Great Potential to Implement the Advances Paradigm of 3P Medicine. *Mass Spectrom. Rev.* **2019**, doi:10.1002/mas.21612.

203. Bubnov, R.; Polivka, J.; Zubor, P.; Konieczka, K.; Golubnitschaja, O. “Pre-Metastatic Niches” in Breast Cancer: Are They Created by or Prior to the Tumour Onset? “Flammer Syndrome” Relevance to Address the Question. *EPMA J* **2017**, *8*, 141–157, doi:10.1007/s13167-017-0092-8.

204. Goldstein, E.; Yeghiazaryan, K.; Ahmad, A.; Giordano, F.A.; Fröhlich, H.; Golubnitschaja, O. Optimal Multiparametric Set-up Modelled for Best Survival Outcomes in Palliative Treatment of Liver Malignancies: Unsupervised Machine Learning and 3 PM Recommendations. *EPMA J* **2020**, *11*, 505–515, doi:10.1007/s13167-020-00221-2.

205. Ferraz, C.R.; Carvalho, T.T.; Manchope, M.F.; Artero, N.A.; Rasquel-Oliveira, F.S.; Fattori, V.; Casagrande, R.; Verri, W.A. Therapeutic Potential of Flavonoids in Pain and Inflammation: Mechanisms of Action, Pre-Clinical and Clinical Data, and Pharmaceutical Development. *Molecules* **2020**, *25*, 762, doi:10.3390/molecules25030762.

206. Bosch-Barrera, J.; Corominas-Faja, B.; Cuyàs, E.; Martin-Castillo, B.; Brunet, J.; Menendez, J.A. Silibinin Administration Improves Hepatic Failure Due to Extensive Liver Infiltration in a Breast Cancer Patient. *Anticancer Res* **2014**, *34*, 4323–4327.

207. Harati, K.; Behr, B.; Wallner, C.; Daigeler, A.; Hirsch, T.; Jacobsen, F.; Renner, M.; Harati, A.; Lehnhardt, M.; Becerikli, M. Anti‑proliferative Activity of Epigallocatechin‑3‑gallate and Silibinin on Soft Tissue Sarcoma Cells. *Mol Med Rep* **2017**, *15*, 103–110, doi:10.3892/mmr.2016.5969.

208. Sakai, H.; Tabata, S.; Kimura, M.; Yabe, S.; Isa, Y.; Kai, Y.; Sato, F.; Yumoto, T.; Miyano, K.; Narita, M.; et al. Active Ingredients of Hange-Shashin-to, Baicalelin and 6-Gingerol, Inhibit 5-Fluorouracil-Induced Upregulation of CXCL1 in the Colon to Attenuate Diarrhea Development. *Biol Pharm Bull* **2017**, *40*, 2134–2139, doi:10.1248/bpb.b17-00479.

209. Samare-Najaf, M.; Zal, F.; Safari, S.; Koohpeyma, F.; Jamali, N. Stereological and Histopathological Evaluation of Doxorubicin-Induced Toxicity in Female Rats’ Ovary and Uterus and Palliative Effects of Quercetin and Vitamin E. *Hum Exp Toxicol* **2020**, *39*, 1710–1724, doi:10.1177/0960327120937329.

210. Ehsan, N.; Ijaz, M.U.; Ashraf, A.; Sarwar, S.; Samad, A.; Afzal, G.; Andleeb, R.; Al-Misned, F.A.; Al-Ghanim, K.A.; Ahmed, Z.; et al. Mitigation of Cisplatin Induced Nephrotoxicity by Casticin in Male Albino Rats. *Braz J Biol* **2021**, *83*, e243438, doi:10.1590/1519-6984.243438.

211. Leong, D.J.; Choudhury, M.; Hanstein, R.; Hirsh, D.M.; Kim, S.J.; Majeska, R.J.; Schaffler, M.B.; Hardin, J.A.; Spray, D.C.; Goldring, M.B.; et al. Green Tea Polyphenol Treatment Is Chondroprotective, Anti-Inflammatory and Palliative in a Mouse Posttraumatic Osteoarthritis Model. *Arthritis Res Ther* **2014**, *16*, 508, doi:10.1186/s13075-014-0508-y.

212. Kaswan, N.K.; Mohammed Izham, N.A.B.; Tengku Mohamad, T.A.S.; Sulaiman, M.R.; Perimal, E.K. Cardamonin Modulates Neuropathic Pain through the Possible Involvement of Serotonergic 5-HT1A Receptor Pathway in CCI-Induced Neuropathic Pain Mice Model. *Molecules* **2021**, *26*, 3677, doi:10.3390/molecules26123677.

213. Siracusa, R.; Monaco, F.; D’Amico, R.; Genovese, T.; Cordaro, M.; Interdonato, L.; Gugliandolo, E.; Peritore, A.F.; Crupi, R.; Cuzzocrea, S.; et al. Epigallocatechin-3-Gallate Modulates Postoperative Pain by Regulating Biochemical and Molecular Pathways. *International Journal of Molecular Sciences* **2021**, *22*, 6879, doi:10.3390/ijms22136879.

214. Warden, B.A.; Smith, L.S.; Beecher, G.R.; Balentine, D.A.; Clevidence, B.A. Catechins Are Bioavailable in Men and Women Drinking Black Tea throughout the Day. *J Nutr* **2001**, *131*, 1731–1737, doi:10.1093/jn/131.6.1731.

215. Kim, J.K.; Park, S.U. Quercetin and Its Role in Biological Functions: An Updated Review. *EXCLI J* **2018**, *17*, 856–863, doi:10.17179/excli2018-1538.

216. Seifirad, S.; Haghpanah, V. Inappropriate Modeling of Chronic and Complex Disorders: How to Reconsider the Approach in the Context of Predictive, Preventive and Personalized Medicine, and Translational Medicine. *EPMA J* **2019**, *10*, 195–209, doi:10.1007/s13167-019-00176-z.

217. Wang, S.; DeGroff, V.L.; Clinton, S.K. Tomato and Soy Polyphenols Reduce Insulin-like Growth Factor-I-Stimulated Rat Prostate Cancer Cell Proliferation and Apoptotic Resistance in Vitro via Inhibition of Intracellular Signaling Pathways Involving Tyrosine Kinase. *J Nutr* **2003**, *133*, 2367–2376, doi:10.1093/jn/133.7.2367.

218. Miltyk, W.; Craciunescu, C.N.; Fischer, L.; Jeffcoat, R.A.; Koch, M.A.; Lopaczynski, W.; Mahoney, C.; Jeffcoat, R.A.; Crowell, J.; Paglieri, J.; et al. Lack of Significant Genotoxicity of Purified Soy Isoflavones (Genistein, Daidzein, and Glycitein) in 20 Patients with Prostate Cancer. *Am J Clin Nutr* **2003**, *77*, 875–882, doi:10.1093/ajcn/77.4.875.

219. Napora, J.K.; Short, R.G.; Muller, D.C.; Carlson, O.D.; Odetunde, J.O.; Xu, X.; Carducci, M.; Travison, T.G.; Maggio, M.; Egan, J.M.; et al. High Dose Isoflavones Do Not Improve Metabolic and Inflammatory Parameters in Androgen Deprived Men with Prostate Cancer. *J Androl* **2011**, *32*, 40–48, doi:10.2164/jandrol.110.010983.

220. Hamilton-Reeves, J.M.; Banerjee, S.; Banerjee, S.K.; Holzbeierlein, J.M.; Thrasher, J.B.; Kambhampati, S.; Keighley, J.; Van Veldhuizen, P. Short-Term Soy Isoflavone Intervention in Patients with Localized Prostate Cancer: A Randomized, Double-Blind, Placebo-Controlled Trial. *PLoS One* **2013**, *8*, e68331, doi:10.1371/journal.pone.0068331.

221. Wang, P.; Aronson, W.J.; Huang, M.; Zhang, Y.; Lee, R.-P.; Heber, D.; Henning, S.M. Green Tea Polyphenols and Metabolites in Prostatectomy Tissue: Implications for Cancer Prevention. *Cancer Prev Res (Phila)* **2010**, *3*, 985–993, doi:10.1158/1940-6207.CAPR-09-0210.

222. Wang, Z.; Desmoulin, S.; Banerjee, S.; Kong, D.; Li, Y.; Deraniyagala, R.L.; Abbruzzese, J.; Sarkar, F.H. Synergistic Effects of Multiple Natural Products in Pancreatic Cancer Cells. *Life Sci* **2008**, *83*, 293–300, doi:10.1016/j.lfs.2008.06.017.

223. Dominiak, K.; McKinney, J.; Heilbrun, L.K.; Sarkar, F.H. Critical Need for Clinical Trials: An Example of a Pilot Human Intervention Trial of a Mixture of Natural Agents Protecting Lymphocytes Against TNF-α Induced Activation of NF-ΚB. *Pharm Res* **2010**, *27*, 10.1007/s11095-010-0113-y, doi:10.1007/s11095-010-0113-y.

224. Karamać, M. Chelation of Cu(II), Zn(II), and Fe(II) by Tannin Constituents of Selected Edible Nuts. *International Journal of Molecular Sciences* **2009**, *10*, 5485–5497, doi:10.3390/ijms10125485.

225. Harper, C.E.; Patel, B.B.; Wang, J.; Lamartiniere, C.A. Epigallocatechin-3-Gallate (EGCG) down-Regulates the Androgen Receptor and the IGF Pathway in the Prostate of TRAMP Mice. *Cancer Res* **2006**, *66*, 1148–1148.
